# Statistical Approach for Biologically Relevant Gene Selection from High-Throughput Gene Expression Data

**DOI:** 10.3390/e22111205

**Published:** 2020-10-25

**Authors:** Samarendra Das, Shesh N. Rai

**Affiliations:** 1 Division of Statistical Genetics, Indian Council of Agricultural Research (ICAR)-Indian Agricultural Statistics Research Institute, PUSA, New Delhi 110012, India; samarendra.das@louisville.edu; 2 Netaji Subhas-Indian Council of Agricultural Research (ICAR) International Fellow, Indian Council of Agricultural Research, Krishi Bhawan, New Delhi 110001, India; 3 Biostatistics and Bioinformatics Facility, JG Brown Cancer Center, University of Louisville, Louisville, KY 40292, USA; 4 School of Interdisciplinary and Graduate Studies, University of Louisville, Louisville, KY 40292, USA; 5 Alcohol Research Center, University of Louisville, Louisville, KY 40292, USA; 6 Department of Hepatobiology and Toxicology, University of Louisville, Louisville, KY 40292, USA; 7 Department of Bioinformatics and Biostatistics, University of Louisville, Louisville, KY 40292, USA; 8 Wendell Cherry Chair in Clinical Trial Research, University of Louisville, Louisville, KY 40292, USA

**Keywords:** SVM, MRMR, bootstrap, gene expression, biological relevance, subject classification

## Abstract

Selection of biologically relevant genes from high-dimensional expression data is a key research problem in gene expression genomics. Most of the available gene selection methods are either based on relevancy or redundancy measure, which are usually adjudged through post selection classification accuracy. Through these methods the ranking of genes was conducted on a single high-dimensional expression data, which led to the selection of spuriously associated and redundant genes. Hence, we developed a statistical approach through combining a support vector machine with Maximum Relevance and Minimum Redundancy under a sound statistical setup for the selection of biologically relevant genes. Here, the genes were selected through statistical significance values and computed using a nonparametric test statistic under a bootstrap-based subject sampling model. Further, a systematic and rigorous evaluation of the proposed approach with nine existing competitive methods was carried on six different real crop gene expression datasets. This performance analysis was carried out under three comparison settings, i.e., subject classification, biological relevant criteria based on quantitative trait loci and gene ontology. Our analytical results showed that the proposed approach selects genes which are more biologically relevant as compared to the existing methods. Moreover, the proposed approach was also found to be better with respect to the competitive existing methods. The proposed statistical approach provides a framework for combining filter and wrapper methods of gene selection.

## 1. Background

The emergence of high-throughput sequencing technologies exponentially increase the size of output data in genome sciences with respect to a number of features [1]. For example, gene expression (GE) studies generate the expression measurements of several thousand(s) of genes for tissue samples over two contrasting conditions in a single study [2,3]. These huge amounts of expression data are being generated for complex traits, and are deposited in public domain databases, such as NCBI GEO, ArrayExpress, etc., over the years by researchers across the globe [4,5]. Further, these publicly available high-throughput data need to be analyzed in order to gain valid biological insights. One such aspect of this research is to select genes, which are highly relevant to the phenotype/trait under study, out of several thousands of genes in the data. This is called feature selection in machine learning in general and gene selection in genomics [5,6,7]. Gene selection has been the focused area of functional genomics research, and thus several statistical and machine learning approaches have been developed for this purpose [8,9]. Here, the main aim is to select relevant genes which are highly informative for the condition/trait (i.e., reduce the curse of high-dimensionality in GE data [5,6,10,11]), and use them as predictors for diagnosing a disease [7,8,12,13] or to understand the stress response mechanisms in plants [6,10]. Further, the selected genes can also be used as predictors for other predictive analysis, i.e., subjects classification [7,8,11], gene regulation modeling [14], gene network analysis [5,6], etc., which enhances the stability, power and feasibility of the developed models [15]. 

Gene selection methods can be grouped into: (i) filter; and (ii) wrapper methods [9,16]. Filter methods select individual genes or gene subset based on a performance measure computed from the data with respect to class variables regardless of the predictive modeling algorithm [17]. These methods include univariate approaches such as *t*-test [18,19], Fold change [19], F-score [20,21], Volcano plot [18], Wilcoxon’s statistic (Wilcox) [22,23], information gain (IG) [9,24], gain ratio (GR) [9,24], symmetric uncertainty [19], etc. These methods select genes by only considering their relevance within a level of the experimental condition/trait. However, these approaches may not be sufficient to discover some complex relationships among genes (i.e., gene-gene interactions) for certain conditions/traits, under which the data is generated [10]. Therefore multivariate filter approaches, such as Pearson’s Correlation (PCR), Spearman’s rank correlation [9,24], Maximum Relevance and Minimum Redundancy (MRMR) [20,25,26], etc. have been developed to select genes from GE data [9,16]. Recently, MRMR method was applied to single-cell transcriptomics data for selection of relevant transcripts responsible for colorectal cancer [27]. 

Wrapper methods select gene subsets by assessing the performance of the predictive modelling algorithm [28]. In other words, this class of gene selection methods are embedded in the classification process. For instance, a wrapper method evaluates the gene subsets based on the classifiers’ performance on GE data and selects the most relevant gene subset. However, the Wrapper methods have better performance over filter methods [9,16], but are more complex, and computationally expensive [28]. This class includes support vector machine-recursive feature elimination (SVM-RFE) [8,29], multiple SVM-RFE (MSVM-RFE) [30], Monte Carlo feature selection algorithm (with SVM classifier) [31] and random forest (RF) [11] to name a few. Further, hybrids of filter and wrapper methods are also reported in literature (known as embedded methods [9]) such as combination of SVM-RFE with MRMR weights (SVM-MRMR) [13], SVM with F-score and other methods [21] to select relevant genes from GE data. Moreover, the MRMR method [20] in conjunction with incremental feature selection and Dagging algorithms [32] were used for gene selection through integrating cross platforms data such as expression quantitative trait loci and genome-wide association study [33]. 

Besides hybrid gene selection methods through combining ReliefF with ant colony optimization [34] and particle swarm optimization [35], algorithms are also developed to select cancer-responsible genes from GE data. Moreover, the existing methods select genes through the weights (i.e., gene ranking criteria) computed from single high-dimensional GE data, which leads to the selection of spuriously associated and redundant genes (i.e., genes may not be informative but are correlated with other relevant genes) [5,6]. Therefore, the permutation procedures are used to compute statistical significance values for genes [6]. However, it has some serious limitations, such as being highly sensitive to a small permutation of experimental conditions (i.e., class labels) [5,6], computationally slow [36,37], cannot possibly give any significant *p*-values after multiple testing adjustments [37,38] and large permutations are required to get a significant *p*-value [37]. To address such issues, bootstrap procedures are used in gene selection which ably remove the spurious associations of genes with the classes and other genes [5,6,39].

Gene selection methods are mostly used to select cancer-responsible genes from GE datasets, and subsequently used for patient classification (e.g., with and without cancer) [6,7,8,13,15,34,35,36,37,38,39,40]. There are limited studies available in literature to systematically explore the performance of gene selection methods on crop GE datasets as there are typically limited experimental data available. Further, the performance of the existing methods were usually assessed through computation of post selection classification accuracy (CA) on cancer GE datasets [7,8,13,15,39,40]. In other words, these techniques are adjudged based on their ability to discriminate the GE samples between case and control groups through training classifiers like SVM [31]. Here, it is worthy to note, this traditional criterion is statistically sound but may not be biologically relevant for performance evaluation of gene selection methods [39,41]. For instance, a gene selection technique identified a set of genes which accurately predicted the class of GE samples for a salinity vs. control GE study in rice, but it fails to tell whether these selected genes are biologically relevant or not to the salinity stress. Hence, it is pertinent to evaluate the gene selection methods with respect to biology-based criteria. For this purpose, data related to traits, such as quantitative trait locus (QTLs) and gene ontology (GO) for model crop plants may be used, which are hugely available in public domains.

We, therefore, propose an improved statistical approach (BSM=Bootstrap-SVM-MRMR) that combines MRMR filter with SVM wrapper method to minimize the redundancy among genes and improve the relevancy of genes with the traits/phenotype under a sound statistical setup. Through this, relevant genes are selected from a high-dimensional GE data through the statistical significance values computed using a nonparametric (NP) test statistic under a bootstrap-based subject sampling model. Further, the comparative performance analysis of the proposed BSM approach is carried out with nine existing competitive methods (i.e., IG [9,24], GR [9,24], *t*-test [18,19], F-score [20,21], MRMR [12,20], SVM-RFE [8,29], SVM-MRMR [13], PCR [9,24] and Wilcox [22,23]). The comparative performance measures include CA along with its standard error computed through varying sliding windows size technique, and three biological criteria based on QTL [42] and GO [43] terms. We demonstrate these procedures on six publicly available, independent crop GE datasets, and find that the BSM approach outperforms in terms of classification and biological relevance criteria compared to the existing methods. 

## 2. Materials and Methods

### 2.1. Motivation

**Figure 1:**
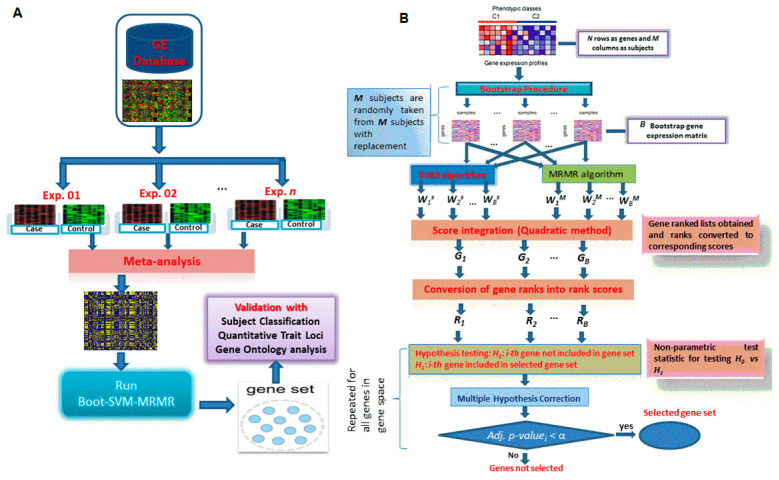
Operational procedure for data integration and the use of proposed BSM approach. (**A**) Outlines for the data integration used in this study for the application of BSM approach. The first step indicates the integration and meta-analysis of GE datasets obtained from various GE studies. Then gene selection methods are applied on the meta GE data. (**B**) Flowchart depicting the implemented algorithm of BSM approach. *W_i_*^(*S*)^’s and *W_i_*^(*M*)^’s are the *N*-dimensional vectors of weights computed through SVM and MRMR approach, respectively. *G_i_*’s and *R_i_*’s are the *N*-dimensional vectors of gene lists and corresponding gene rank scores. SVM and MRMR stand for Maximum Relevance and Minimum Redundancy and support vector machine algorithms. *p_i_*-value is statistical significance value for *i^th^* gene. *α* is the desired level of statistical significance.

The GE datasets, from various experiments conducted to understand the behavior of biological mechanisms, are hugely available in public domain databases. For example, GE datasets generated for 125,376 experiments over 19,893 Microarray platforms consisting of data on 3,406,218 samples are available in NCBI GEO database until the current date [4]. Usually, researchers use data from single experiments to test their methodology or select genes for further study. For instance, Wang et al. (2013) used the salinity stress GE samples from GSE14403 to test their methodology and select salinity responsive genes to understand salinity tolerance mechanism in rice [6]. Such a study is important but may not be enough to test the hypothesis of salinity tolerance in rice due to limited sample size. Hence, the real challenge is to integrate or combine the GE datasets generated for same or cross platforms over different experimental conditions and test the methodology(s) on the meta-data. Moreover, meta-analysis of data generated by GE experiments for the same or related stress(es) is essential to enhance the sensitivity of the hypothesis under consideration for drawing valid biological conclusions. Therefore, we performed meta-analysis on GE datasets corresponding to different stresses from multiple experiments and tested the performance of methods on these metadata, as shown in Table 1. The outlines of meta-analysis are given in Figure 1A.

### 2.2. Data Source

Rice GE experimental datasets were collected from the Gene Expression Omnibus database (GEO) of NCBI for platforms GPL2025 (www.ncbi.nlm.nih.gov/geo/query/acc.cgi?acc=GPL2025) [4]. Here, we used the rice data, as it is a model crop plant, has a large amount of GE, other related biological (QTL and GO) datasets are available publicly, and its genome is well annotated. The selected GE datasets were generated under biotic (bacterial (*Xanthomonas*), fungal (Blast), insect (Brown plant hopper) and abiotic (salinity, cold and drought) stresses in rice. The summary and details of these datasets are given in Table 1 and Supplementary Table S1, respectively. Initially, the raw CEL files of the collected samples were processed using Robust Multichip Average algorithm available in *affy* Bioconductor package of R [44]. This procedure involves background correction, quantile normalization and summarization by median polish approach. Further, the log2 scale transformed expression data for the collected experimental samples were used for meta-analysis to remove the outlier samples (Supplementary Document S1). The GE samples from 3, 4, 5, 3 and 2 independent studies for salinity, cold, drought, bacterial and fungal stresses, respectively, were integrated (Table 1) through the meta-analysis (under the parameters settings in Supplementary Table S2) to obtain the meta-data. For instance, the salinity stress dataset, originating from 3 independent studies, are available in GEO database under the accession numbers GSE14403, GSE16108 and GSE6901 and consist of expression measurements for over 45 samples. Then, these meta-datasets for the respective stresses were further used to remove the control and irrelevant features through the preliminary genes selection to reduce the computational complexity and dimensions of the datasets. For instance, out of 57,381 genes in drought stress, the control (123) and irrelevant (48180) genes were filtered out by setting the fold change and *p*-value (from *t*-test) parameters as 1 and 0.05, respectively, through the preliminary gene selection. The detail process of data collection, meta-analysis and preliminary gene selection for the datasets are given in Supplementary Document S1. Then, the processed datasets (Table 1) were used for further data analysis. Further, the QTL datasets for the stresses in rice, viz. salinity, drought, cold, insect, fungal and bacterial, were collected from the Gramene QTL database (http://www.gramene.org/qtl/) [45]. The lists of the respective stress responsive QTLs along with their mapped positions on the genome are given in Supplementary Document S2. The GO annotations data of the rice genome used in this study were collected from *AgriGO* database [46]. 

### 2.3. Methods

#### 2.3.1. Notations

Let X*_N_*_× *M*_ = [*x_im_*] be the GE data matrix, where *x_im_* represents the expression of *i^th^* (*i* = 1, 2, …, *N*) gene in *m^th^* (*m* = 1, 2, …, *M*) sample/subject; *x_m_* be the *N*-dimensional vector of expression values of genes for *m^th^* sample; $y_{m}$ be the outcome variable for target class label of *m^th^* sample and take values {+1, −1} for case and control conditions, respectively; *M*_1_ and *M*_2_ be the number of GE samples in case and control classes, respectively, ($M_{1}+M_{2}=M$); $\left({\bar{x}}_{i1},\;S_{i1}^{2})\;\mathrm{and}\;({\bar{x}}_{i2},\;S_{i2}^{2}\right)$ be the mean and variance of *i^th^* gene for case and control classes, respectively; ${\bar{x}}_{i}$ be the mean of *i^th^* gene across all *M* samples; $S_{ij}$ be the covariance between *i^th^* and *j^th^* genes.

#### 2.3.2. Maximum Relevance and Minimum Redundancy (MRMR) Filter

MRMR method aims at selecting maximally relevant and minimally redundant sets of genes for discriminating the tissue samples (e.g., case vs. control). This method is extensively used for selection of cancer-responsible genes from high-dimensional GE data for patient classification (i.e., with and without cancer) [12,20,26]. For continuous GE data (e.g., Microarrays), the relevance measure for *i^th^* gene over the given classes (i.e., case and control) is computed through F-statistic [12] and is expressed as:

(1) $$F\left(i\right)=\frac{M_{1}{\left({\bar{x}}_{i1}-{\bar{x}}_{i}\right)}^{2}+M_{2}{\left({\bar{x}}_{i2}-{\bar{x}}_{i}\right)}^{2}}{\{(M_{1}-1)S_{i1}^{2}+(M_{2}-1)S_{i2}^{2}\}/\left(M-2\right)}$$

Further, the redundancy measure in MRMR method is computed through Pearson’s correlation (ignoring the class information) for continuous GE data [12] and is given as

(2) $$R\left(i,j\right)=Corr\left(x_{i},x_{j}\right)=\frac{S_{i,j}}{S_{i}S_{j}}=\frac{\sum_{m=1}^{M}\left(x_{im}-{\bar{x}}_{i}\right)\left(x_{jm}-{\bar{x}}_{j}\right)}{\sqrt{\sum_{m=1}^{M}{\left(x_{im}-{\bar{x}}_{i}\right)}^{2}}\sqrt{\sum_{m=1}^{M}{\left(x_{jm}-{\bar{x}}_{j}\right)}^{2}}}$$

In MRMR method, genes are ranked by the combination of relevance, and redundancy measures under F-score with correlation quotient scheme for continuous GE data [12,20,26]. The weights computed through MRMR method for gene ranking can be expressed in terms of Equations (1) and (2) and is given as:

(3) $$w_{i}=F\left(i\right)/\{\frac{1}{N-1}\sum_{\begin{array}{c} j=1\\ j\neq i \end{array}}^{N}\left|R\left(i,j\right)\right|\}\;\;\;\;\;\;\;\;\;\;\;\;\;\;\forall \;\;i=1,\;2,\;\ldots ,\;N$$

where *w_i_* (≥0) is the weight associated with *i^th^* gene. The functions *F*(*i*) and *R*(*i*, *j*) in Equation (3) represent the F-statistic for *i^th^* gene and Pearson’s correlation coefficient between *i^th^* and *j^th^* genes. In other words, *i^th^* gene weight is F-statistic adjusted with average absolute correlation of *i^th^* gene with the remaining genes.

#### 2.3.3. Support Vector Machine (SVM)

SVM method is used for selection of genes (in a 2 group case) from high-dimensional GE data [29]. Let $\left\{x_{m},y_{m}\right\}\;\in \;R^{N}\;\times \;\left\{-1,\;1\right\}$ be the input given to SVM. Here, we wish to find out a hyperplane that divides the GE samples/subjects for case $(y_{m}=1){\bar{x}}_{i}$ from that of control class ($y_{m}=-1)$ in such a way that the distance between the hyperplane and the point, ${\bar{x}}_{i}$ $x_{m}$, is maximum. Then the hyperplane can be written as:

(4) $$\sum_{i=1}^{N}k_{i}x_{im}+b=0\;\;\;\;\;\;\;\;\;\;\;\forall \;m=1,\;2,\ldots ,\;M$$

where *k_i_* and *b* are the weight of *i^th^* gene and bias, respectively. Here, we assume that the GE samples for 2 classes are linearly separable. In other words, we can select 2 parallel hyperplanes that separate the case and control classes in such a way that the distance between them is maximum.

For case class, the hyperplane becomes:

(5) $$\sum_{i=1}^{N}k_{i}x_{ip}+b=1\;\;\;\;\;\;\;\;\;\;\;\mathrm{for}\;\mathrm{any}\;p=1,\;2,\ldots ,\;M_{1}$$

For control class, the hyperplane becomes:

(6) $$\sum_{i=1}^{N}k_{i}x_{iq}+b=-1\;\;\;\;\;\;\;\;\;\mathrm{for}\;\mathrm{any}\;q=1,\;2,\ldots ,\;M_{2}$$

The expressions in Equations (5) and (6) can be combined as:

(7) $$y_{m}\left(\sum_{i=1}^{N}k_{i}x_{im}+b\right)=1\;\;\;\;\;\;\;\;\forall \;m=1,\;2,\ldots ,\;M$$

Here, we wish to maximize the distance between the case, and control hyperplanes in Equation (5) and Equation (6), respectively, under the constraint that there will be no GE samples between these 2 hyperplanes given in Equation (7). Mathematically, it can be written as:

(8) $$\overset{N}{\underset{i=1}{\sum }}\frac{k_{i}}{{\left(\sum k_{i}\right)}^{2}}\left|x_{ip}-x_{iq}\right|=\frac{2}{{\left(\sum k_{i}\right)}^{2}}$$

So, to maximize the distance between the planes in Equation (8), we need to minimize $\frac{{\left(\sum_{i}k_{i}\right)}^{2}}{2}$ under the constraint of Equation (7). Mathematically, it can be written as:

(9) $$L_{p}=\underset{k_{i}}{\min}\frac{{\left(\sum_{i}k_{i}\right)}^{2}}{2}+\sum_{m=1}^{M}\varphi_{m}\left\{1-y_{m}\left(\sum_{i=1}^{N}k_{i}x_{im}+b\right)\right\}\;\;\;\forall \;m=1,\;2,\ldots ,\;M$$

where $\varphi_{m}$ (≥0): Lagrange multiplier. Here, *k_i_*’s are obtained by minimizing the objective function in Equation (9). Through the principle of maxima-minima, we have:

(10) $$\frac{\partial L_{p}}{\partial k_{i}}=\sum_{i}k_{i}-\sum_{i}(\sum_{m=1}^{M}\varphi_{m}y_{m}x_{im})=0\;\mathrm{and}\;\frac{\partial L_{p}}{\partial b}=\sum_{m=1}^{M}\varphi_{m}y_{m}=0$$

The value of $k_{i}$ can be obtained through solving the system of linear equations given in Equation (10) and is expressed as:

(11) $$k_{i}=\sum_{m=1}^{M}\varphi_{m}y_{m}x_{im}\;\;\;\mathrm{with}\;\sum_{m=1}^{M}\varphi_{m}y_{m}=0\;\mathrm{and}\;\varphi_{m}\geq \;0$$

Here, $\left|k_{i}\right|\;\left(\geq 0\right)$ in Equation (11) is used as a metric for the ranking of genes in the GE data [29]. Alternatively, $k_{i}^{2}$ as a gene ranking metric can also be derived by using Taylor series approximation [47], which is given in Supplementary Document S3.

#### 2.3.4. Proposed Hybrid Approach of Gene Selection

MRMR method may not yield optimal CA because it performs independently of the classifier and is only involved in selection of genes [13]. On the contrary, SVM method of gene selection does not consider the redundancy among genes (i.e., gene-gene correlations) while selecting genes [13]. Hence, Mundra and Rajapakse (2010) have developed a gene selection method by taking linear combination of weights computed through MRMR and SVM methods [13], and is given as:

(12) $$SL_{i}=\delta w_{i}+\left(1-\delta \right)|k_{i}|$$

where parameter δ∈[0, 1] decides the tradeoff between SVM and MRMR weights. The $SL_{i}$ in Equation (12) is highly dependent on the value of δ. In other words, the choice of δ may alter the order of genes by MRMR (*w_i_*) or by SVM (*k_i_*), especially when *w_i_* and *k_i_* are negatively correlated. Hence, we propose a statistical approach by combining SVM and MRMR weights under sound statistical framework, where genes are selected through *p*-values computed using the NP test statistic, which is described as follows.

First, we normalized the $w_{i}$ and *k_i_*’s through minimax normalization. Then $w_{i}$ and *k_i_* were ranked based on the ascending order of their magnitudes and assigned ranks $\gamma_{i}^{MR}$ and $\gamma_{i}^{SV}$ for *i^th^* gene, respectively. Then, we developed a technique, i.e., quadratic integration, for integrating the gene scores based on ranks, which automatically assigned more weights to the higher value of *w_i_* and *k_i_*. Now, the quadratic integration score can be expressed as:

(13) $$SD_{i}=\frac{\beta \gamma_{i}^{MR}w_{i}{}^{norm}+\left(1-\beta \right)\gamma_{i}^{SV}{\left|k_{i}\right|}^{norm}}{\beta \gamma_{i}^{MR}+\left(1-\beta \right)\gamma_{i}^{SV}}$$

where $w_{i}{}^{norm}$ and ${\left|k_{i}\right|}^{norm}$ are the normalized values, expressed in Equation (14) and Equation (15), respectively.

(14) $$w_{i}{}^{norm}=\left(w_{i}-\underset{i}{\min}w_{i})/(\underset{i}{\max}w_{i}-\underset{i}{\min}w_{i}\right)$$

(15) $${\left|k_{i}\right|}^{norm}=\left(\left|k_{i}\right|-\underset{i}{\min}\left|k_{i}\right|)/(\underset{i}{\max}\left|k_{i}\right|-\underset{i}{\min}\left|k_{i}\right|\right)$$

Further, β(∈(0, 1)) in Equation (13) is determined empirically from the data through a 5-fold cross validation technique. The detail procedure for determining the optimum value of β is given in Supplementary Document S4. If *SD_i_* in Equation (13) is used alone for ranking of genes, it will become a filter approach and lead to selection of spuriously associated genes. Hence, we used a bootstrap procedure under a subject sampling model setup to obtain the empirical distribution of *SD_i_* for computation of statistical significance value for *i^th^* (*i* = 1, 2, …, *N*) gene. Here, the used bootstrap procedure is described below.

The *M* samples (as columns) in the GE data matrix, either belonging to case or control, can be considered as subjects/units in a population model, as shown in Equation (16).

(16) $$\left(x_{1},y_{1}\right),\;\left(x_{2},y_{2}\right),\ldots ,\;\left(x_{m},y_{m}\right),\ldots ,\;\left(x_{M-1},y_{M-1}\right),\left(x_{M},y_{M}\right)\;$$

Here, we assume that the subjects are independent and identically distributed, but the genes within each subject may be correlated. In the bootstrap procedure, *M* units are randomly drawn from *M* population units in Equation (16) with a replacement to constitute a bootstrap GE data matrix, i.e., $X_{NXM}^{\left(b\right)}$ (*M* units serve as *M* columns of *X*). This process is repeated *B* times to get *B* bootstrap GE data matrices, i.e., $X_{NXM}^{\left(1\right)},\;X_{NXM}^{\left(2\right)},\ldots ,\;X_{NXM}^{\left(b\right)},\;\ldots ,\;X_{NXM}^{\left(B\right)}$. Here, *B* (i.e., number of bootstrap samples) depends on several factors, such as number of units in the population model in Equation (16) and must be sufficiently large. So, we set *B* = 200 as several empirical studies showed that the number of bootstrap samples required for an estimation procedure is ~200 [6,48].

Now, the *B* bootstrap GE data matrices are given as the input to Equations (3), (11) and (13) to compute the *SD* scores, and subsequently gene ranking was performed on each of the *B* bootstrap GE data matrices.

Let *P_ib_*, be a random variable (*rv*) that shows the position of *i^th^* gene in *b^th^* bootstrap GE matrix. Then, another *rv* can be defined based on *P_ib_* (without loss of generality), given as:

(17) $$R_{ib}=\frac{N+1-P_{ib}}{N};\;0\leq R_{ib}\leq 1$$

where $R_{ib}$ in Equation (17) is the rank score of *i^th^* (*i* = 1, 2, …, *N*) gene in *b^th^* (*b* = 1, 2, …, *B*) bootstrap GE matrix. Here, it may be noted that the distribution of the rank scores of genes, computed from a bootstrap GE data matrix, is symmetric around the median value (as rank scores are a function of ranks). The values of the median and the third quartile ($Q_{3}$) are given as 0.5 and 0.75, respectively.

To decide whether *i^th^* gene is biologically relevant or not to the condition/trait under study, the following null hypothesis can be tested.

$$H_{0}:R_{i}\leq Q_{3}\;\left(i-th\;\mathrm{gene}\;\mathrm{is}\;\mathrm{not}\;\mathrm{so}\;\mathrm{relevant}\;\mathrm{to}\;\mathrm{the}\;\mathrm{trait}\right)\;H_{1}:R_{i}>Q_{3}\;\left(i-th\;\mathrm{gene}\;\mathrm{is}\;\mathrm{relevant}\;\mathrm{to}\;\mathrm{the}\;\mathrm{trait}\right)$$

where $R_{i}$ is the rank score for *i^th^* gene over all possible bootstrap samples.

To obtain the distribution of test statistic under *H*_0_, we define another *rv* $Z_{ib}$, as:

(18) $$Z_{ib}=\{\begin{array}{c} 1\;\;\left|R_{ib}-Q_{3}\right|>0\\ 0\;\;\left|R_{ib}-Q_{3}\right|<0 \end{array}$$

Let $r_{ib}$ be another *rv* represents the rank assigned to $\left(R_{ib}-Q_{3}\right)$ (after arranging in ascending order of their magnitudes). To test *H*_0_ vs. *H*_1_ the test statistic for *i^th^* gene, $W_{i}$, was developed, and is given as:

(19) $$W_{i}=\sum_{b=1}^{B}Z_{ib}r_{ib}=\sum_{b=1}^{B}U_{ib\;}\left(say\right)$$

In other words, *W_i_* in Equation (19) is the sum of the ranks of positive signed scores for *i^th^* gene over *B* bootstrap samples. Further, $U_{ib}$ in Equation (19) is a Bernoulli *rv,* and its probability mass function can be given as:

(20) $$P\left[U_{ib}=u_{ib}\right]=\{\begin{array}{c} \frac{3}{4}\;\;\;\;\;if\;u_{ib}=0\\ \frac{1}{4}\;\;\;\;\;if\;u_{ib}=1 \end{array}$$

Here, the expected value and variance of *W_i_* in Equation (19) under *H*_0_ can be obtained as:

(21) $$E\left(W_{i}\right)=\sum_{b=1}^{B}E(U_{ib\;})=\sum_{b=1}^{B}(0.\frac{3}{4}+b.\frac{1}{4})=\frac{1}{4}\sum_{b=1}^{B}b=\frac{B\left(B+1\right)}{8}$$

The variance becomes:

(22) $$\begin{array}{c} V\left(W_{i}\right)=E\left(W_{i}^{2}\right)-{\left[E\left(W_{i}\right)\right]}^{2}\\ =\sum_{b=1}^{B}E\left(U_{ib}^{2}\right)-\sum_{b=1}^{B}E{\left(U_{ib\;}\right)}^{2}\\ =\sum_{b=1}^{B}\left(\frac{b^{2}}{4}-{\frac{b}{16}}^{2}\right)=\frac{B\left(B+1\right)\left(2B+1\right)}{32} \end{array}$$

As *B* is sufficiently large, then under central limit theorem, the distribution of *W_i_* in Equation (19) becomes:

(23) $$Z_{i}=\frac{W_{i}-E\left(W_{i}\right)}{\sqrt{V(W_{i)}}}\overset{d}{\to }\;N\left(0,\;1\right)$$

Through Equation (23), the *p*-value for *i^th^* (*i* = 1, 2, …, *N*) gene is computed and similarly this testing procedure is repeated for the remaining *N* − 1 genes. Let $p_{1},\;p_{2},\ldots ,\;p_{N}$ be the corresponding *p*-values for all the genes in GE data, and *α* be the level of significance. Here, we assume that all genes in the GE data are equally important for the trait development, hence, we employed Hochberg procedure [49] for correcting the multiple testing, and to compute the adjusted (*adj.*) *p*-values for genes. It is worthy to note that Hochberg’s procedure is computationally simple, quite popular in genomic data analysis [50] and more powerful than Holm’s procedure [51]. The algorithm for Hochberg’s procedure [49] is as follows.

Step 1. If $p_{\left(l\right)}>\alpha$, then retain corresponding null hypothesis ($H_{\left(l\right)}$) and go to the next step. Otherwise, reject it and stop.

Step i=2, 3, …,N−1. If $p_{\left(N-i+1\right)}>\alpha /i$, then retain $H_{\left(N-i+1\right)}$ and go to the next step. Otherwise, reject all remaining hypotheses and stop.

Step *N*. If $p_{\left(1\right)}>\alpha /N$, then retain ($H_{\left(1\right)}$). Else reject it.

Now, the *adj. p-values* are given recursively beginning with the largest *p*-value [49]:

(24) $$\tilde{p_{\left(i\right)}}=\{{\begin{array}{c} p_{\left(i\right)\;\;\;if\;i=N}\\ \min(\tilde{p_{\;}}\left(i+1\right),\;\left(N-i+1\right)p \end{array}}_{\left(i+1\right)}\;if\;i=N-1,\;\ldots ,1$$

Further, based on the computed *adj.* *p*-values, the relevant genes are selected from the high dimensional GE data. In other words, lesser value of *adj.* *p*-value may indicate more relevance of the gene for the target trait and vice-versa. The outlines and key analytical steps of the proposed BSM approach are shown in Figure 1B.

### 2.4. Comparative Performance Analysis of the Proposed Approach

The comparative performance analysis of the proposed BSM approach with respect to 9 competitive gene selection methods (Supplementary Document S5) was carried out on 6 different rice GE datasets (Table 1). For this purpose, different gene sets (***G***) of various sizes given in Supplementary Table S10 were selected through the 10 gene selection methods including the proposed BSM approach. Then, these gene sets were validated with respect to subject classification, QTL testing and GO analysis.

#### 2.4.1. Performance Analysis with Subject Classification

Under this comparison setting, the performance of the gene selection methods (Supplementary Document S5) including the proposed approach were assessed in terms of subject classification using mean CA and standard error (SE) in CA computed through a varying sliding window size technique [5,39]. Here, we used the varying window size technique to study the impact of gene ranking on classification of subjects. In other words, genes in ***G*** were validated with their ability to discriminate the class labels of subjects/samples between case (+1), and control (−1). Further, the gene set selected through a method which provides maximum discrimination between the subjects of 2 groups (i.e., case vs. control) through CA will be considered as highly relevant gene sets. The expressions for mean CA and SE in CA computed through varying window size technique are given in Equations (25) and (26).

Let *n* be the size of ***G***, *S* be the size of the windows (i.e., size refers to number of ranked genes) and *L* be the sliding length. Then, the total number of windows becomes K=(n−S)/L. The genes in ***G***, arranged in different windows along with their expression values, were then used in SVM classifiers with 4 basis-functions, i.e., linear (SVM-LBF), radial (SVM-RBF), polynomial (SVM-PBF) and Sigmoidal (SVM-SBF) to compute CA over a 5-fold cross validation. Let, *CA*_1_, *CA*_2_, …, *CA_K_* be the CA’s for each sliding windows, then the mean CA and SE in CA can be defined as:

(25) μCAG=(∑k=1KCAk)K

(26) SECAG=∑k=1K(CAk−μCAG)2K

Here, we took different combinations of *n*, *S* and *L*, as given in Supplementary Table S10, to compute $\mu_{CA}^{G}$ and $SE_{CA}^{G}$ for the comparative performance analysis of the gene selection methods (Supplementary Document S5). The higher value of $\mu_{CA}^{G}$ and a lower value of $SE_{CA}^{G}$ indicates the better performance of the gene selection method, and vice-versa.

#### 2.4.2. Performance Analysis with QTL Testing

The comparative criteria based on subject classification are popularly used for assessing the performance of gene selection methods [7,8,12,13,15,39,40]. However, these criteria fail to tell the biological relevancy of the genes selected through the gene selection methods [41]. Hence, under this comparative setting we assessed the performance of the proposed and existing methods through their ability to select genes which are associated with QTL regions. For this purpose, the criteria given in Equations (27) and (29) are developed.

(27) $$Qstat=\sum_{t=1}^{\left|Q\right|}\sum_{i=1}^{n}I_{q_{t}}\left(g_{i}\right)$$

where ***G***: gene set selected by a method, *Qstat: rv* whose values represent the number of genes covered by QTLs, *Q*: set of associated QTLs, and the indicator function present in Equation (27) is represented in Equation (28).

(28) $$I_{q_{t}}\left(g_{i}\right)=\{\begin{array}{c} 1\;\;\;\;\;\;\;if\;g_{i}^{c}\left[a,\;\right]\geq q_{t}^{c}\left[d,\;\right]\;and\;g_{i}^{c}\left[,b\;\right]\leq q_{t}^{c}\left[,e\;\right]\;\\ 0\;\;\;\;\;\;else \end{array}$$

where, *g_i_^c^* [*a*, *b*] ϵ ***G*** (*a* and *b* represent start and stop positions in terms of bp of the gene *g_i_* on chromosome *c*) and *q_t_^c^* [*d*, *e*] ϵ *Q* (*d* and *e* represents the start and stop positions of the QTL *q_t_* on chromosome *c*).

Here, the Qstat *rv* follows a hyper-geometric distribution and its distribution function is given in Equation (29).

(29) $$P\left[Qstat=v\right]=1-\left(\begin{array}{c} M\\ v \end{array}\right)\left(\begin{array}{c} N-V\\ n-v \end{array}\right)/\left(\begin{array}{c} N\\ n \end{array}\right)$$

where *V*: total number of genes covered by the QTLs in the whole GE data and *v*: number of genes in ***G*** that are covered by QTLs. Further, using the Equation (29), the statistical significance value (*p*-value) associated with the ***G*** can be computed. In other words, this *p*-value reveals the enrichment significance of ***G*** with trait specific QTLs. Here, the higher values of Qstat and $-log_{10}$(*p*-value) indicate the better performance of the gene selection method, and vice-versa.

#### 2.4.3. Performance Analysis with GO Enrichment

GO analysis involves the annotation of gene functions under 3 taxonomic categories, i.e., molecular function (MF), biological process (BP) and cellular component (CC) [43]. This analysis helps in evaluating the functional similarities among the genes in ***G*** [52], as there exists a direct relationship between semantic similarity of gene pairs with their structural (sequence) similarity [53,54]. Under this comparison setting, we assessed the performance of 10 gene selection methods including the proposed method using GO based biologically relevant criterion. In other words, first different gene sets were selected through these methods, then GO based criterion was computed for each selected gene set. For this purpose, we developed a GO based semantic distance measure to assess the GO based biologically relevancy of ***G*** selected thorough the proposed and existing gene selection methods. The GO based semantic distance measure (*d_ij_*) between *i^th^* and *j^th^* genes can be expressed in Equation (30), as:

(30) $$d_{ij\;\left(i\neq j\right)}^{GO}=1-\frac{\left|GO_{i}\;\cap \;GO_{j}\right|}{\left|GO_{i}\;\cup \;GO_{j}\right|}\;\;\;\;\;\;\;\;\;\;\;\forall \;i,\;j=1,\;2,\;\ldots ,\;n$$

where *GO_i_* = {*go*_*i*1_, *go*_*i*2_, …, *go_iI_*} and *GO_j_* = {*go*_*j*1_, *go*_*j*2_, …, *go_jJ_*} are the 2 sets of GO terms that annotate *i^th^* and *j^th^* genes in ***G***, respectively. Further, the GO based average biologically relevant score for ***G*** (for a gene selection method) can be developed based on Equation (30) and is shown in Equation (31).

(31) $$D_{G}^{avg}=\frac{2}{n\left(n-1\right)}\sum_{\begin{array}{c} i,j=1\\ i\neq j \end{array}}^{n}d_{ij}^{GO}$$

where $D_{G}^{avg}$ in Equation (31) represents the average biologically relevant score for ***G*** based on GO annotations. Using Equation (31), the $D_{G}^{avg}$ scores under MF, BP and CC taxonomies were computed for each of the gene sets selected through different methods. A lower value of $D_{G}^{avg}$ indicates better performance of the gene selection method and vice-versa.

## 3. Results and Discussion

### 3.1. Computation of Genes Selection Criteria through Proposed Approach

**Figure 2:**
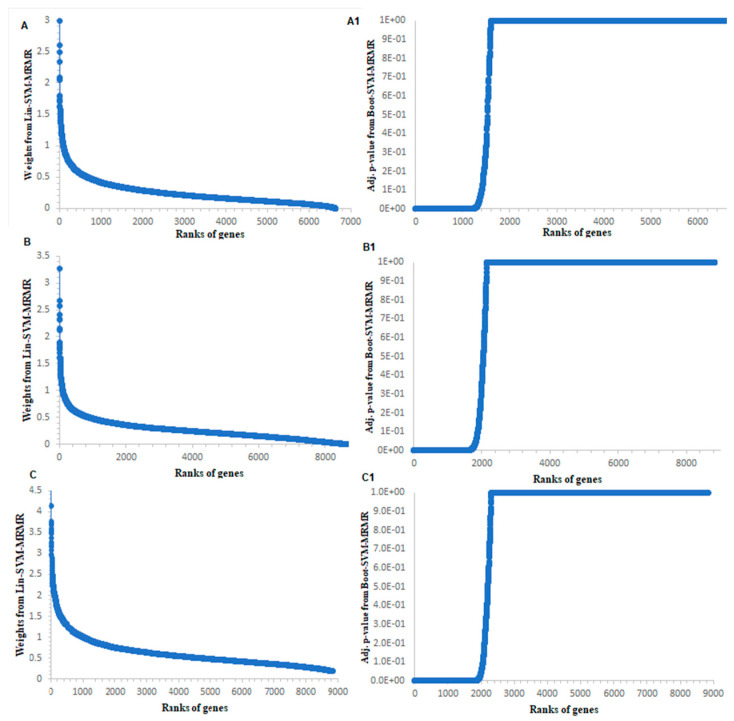
Graphical analysis of the proposed BSM approach with SVM-MRMR approach for abiotic stress datasets. Distribution of gene weights computed from SVM-MRMR approach for the abiotic stresses. The distributions of gene weights from the SVM-MRMR are shown for (**A**) salinity; (**B**) cold; and (**C**) drought stress datasets in rice. Distribution of adjusted *p*-values computed from the proposed BSM approach for the abiotic stresses. The distributions of the adjusted *p*-values are shown for (**A1**) salinity; (**B1**) cold; and (**C1**) drought stress datasets.

The distributions of weights computed from SVM-MRMR method [13] and adj. *p*-values for genes computed from the proposed BSM approach for abiotic and biotic stresses in rice are shown in Figure 2 and Figure S3, respectively. The distributions of SVM-MRMR weights of genes for salinity stress data contained values, which were not so clearly separated (i.e., higher values from lower values) (Figure 2A). In other words, the genes relevant to salinity stress condition were not well visualized from Figure 2A. However, from the distribution of adj. *p*-values computed through the proposed approach, it was observed that the relevant genes were well separated from the irrelevant genes, and a small number of genes found to be statistically significant (i.e., relevant to salinity stress) (Figure 2(A1)). In other words, for salinity stress data, the separation between relevant and irrelevant genes can be well visualized from Figure 2(A1) as compared to Figure 2A. Similar interpretations can be observed for other stress datasets, viz. cold, drought, bacterial, fungal and insect (Figure 2 and Figure S3). Hence, the comparative graphical analysis showed a clear distinction between relevant and irrelevant genes through the proposed BSM approach as compared to the existing SVM-MRMR approach. In other words, this comparative analysis showed the improvement of BSM approach over the SVM-MRMR method (Figure 2 and Figure S3), at least in terms TABLE of visualization. Further, the relevant genes selection using adj. *p*-values computed through the NP test statistic was more statistically sound as it is independent from the distribution of GE data, and corrected over multiple hypothesis testing. These metrics (values between 0 and 1) are scientifically well defined and statistically calculated biologically interpretable values to genome researchers and experimental biologists, as compared to gene ranks/weights. In BSM approach, a significant *p*-value gives confidence that the given gene is relevant for the target condition/trait.

### 3.2. Comparative Performance Analysis Based on Subject Classification

**Figure 3:**
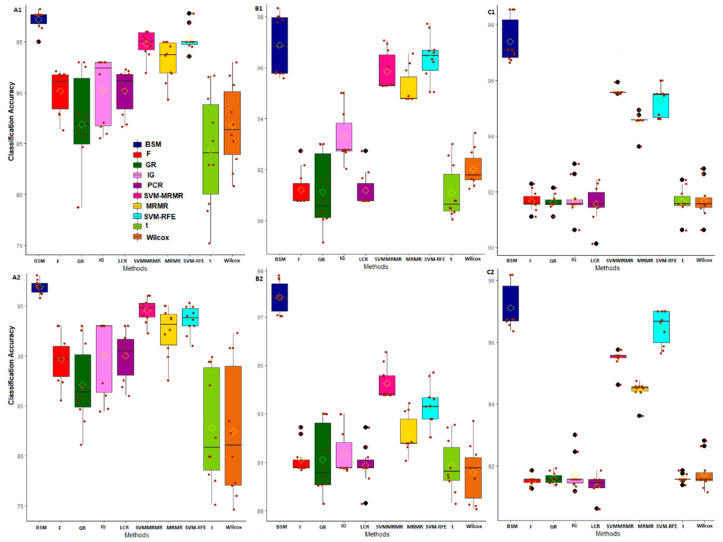
Classification-based comparative performance analysis of gene selection methods through SVM-LBF and SVM-PBF classifiers for abiotic stress datasets. The horizontal axis represents the gene selection methods. The vertical axis represents post selection classification accuracy obtained by using varying sliding window size technique. The classification accuracies over the window sizes are presented as boxes. The bars on the boxes represent the standard errors. The distributions of classification accuracies are shown for cold stress with SVM-LBF (**A1**), and SVM-PBF (**A2**) classifiers. The distributions of classification accuracies are shown for salinity stress with SVM-LBF (**B1**) and SVM-PBF (**B2**) classifiers. The distributions of classification accuracies are shown for drought stress with SVM-LBF (**C1**) and SVM-PBF (**C2**) classifiers.

**Figure 4:**
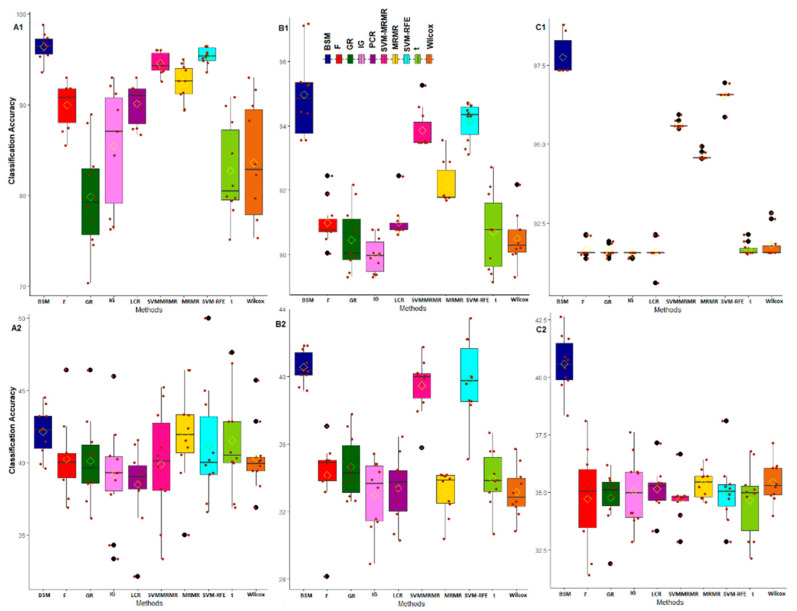
Classification-based comparative performance analysis of gene selection methods through SVM-RBF and SVM-SBF classifiers for abiotic stress datasets. The horizontal axis represents the gene selection methods. The vertical axis represents post selection classification accuracy obtained by using varying sliding window size technique. The classification accuracies over the window sizes are presented as boxes. The distributions of classification accuracies are shown for cold stress with SVM-RBF (**A1**) and SVM-SBF (**A2**) classifiers. The distributions of classification accuracies are shown for salinity stress with SVM-RBF (**B1**) and SVM-SBF (**B2**) classifiers. The distributions of classification accuracies are shown for drought stress with SVM-RBF (**C1**) and SVM-SBF (**C2**) classifiers.

We used $\mu_{CA}^{G}$ and $SE_{CA}^{G}$ computed through the varying sliding window size technique as statistically necessary criteria for performance analysis of the proposed BSM approach on six different GE datasets. Here, these measures were computed over five-fold cross validations through training the SVM-LBF, SVM-PBF, SVM-RBF and SVM-SBF classifiers. The results are shown in Figure 3 and Figure 4 for abiotic stresses and in Supplementary Figure S4 for biotic stresses. The values of CA and SE in CA are also given in Supplementary Document S6. For cold stress data in rice, the $\mu_{CA}^{G}$ computed through SVM-LBF classifier for the proposed BSM approach was observed to be higher than other gene selection methods followed by SVM-RFE and SVM-MRMR over all selected gene sets Figure 3. This indicated the better performance of the BSM approach in terms of its ability to classify the subjects/samples through selecting relevant genes from cold stress GE data. Further, the values of $SE_{CA}^{G}$ from SVM-LBF classifier for the BSM approach was found to be much less (over all the gene sets) than that of nine existing gene selection methods considered in this study (Supplementary Document S6). This shows that the genes selected through the proposed BSM approach is much more relevant (informative), and robust than other methods. For instance, the gene set of size 50 (i.e., optimum gene set) provided satisfactory results in terms of higher $\mu_{CA}^{G}$ and lower $SE_{CA}^{G}$ irrespective of the gene selection methods used (Table S12 of Document S6). For cold stress data, similar interpretations can be made for SVM-PBF, SVM-RBF and SVM-SBF classifiers from Figure 3 and Figure 4.

For salinity stress data, the $\mu_{CA}^{G}$ (except gene sets of sizes 500, 1000 and 1500) computed for the proposed BSM approach through the SVM-LBF classifier were found to be higher than other methods followed by SVM-RFE and SVM-MRMR (Figure 3, Document S6). This indicated the proposed approach was fairly better, and competitive with the popular methods, i.e., SVM-RFE, SVM-MRMR. However, for SVM-PBF classifier, the $\mu_{CA}^{G}$ over all the gene sets for the BSM approach was higher than all other considered gene selection methods followed by SVM-RFE (Figure 3, Document S6). Furthermore, the $SE_{CA}^{G}$ computed through SVM-LBF and SVM-PBF classifiers for the BSM approach was found to be the least followed, by SVM-RFE (Document S6), indicating the selection of robust and relevant gene sets. Similar interpretation can be made for SVM-RBF and SVM-SBF classifiers from Figure 4. It was observed that the $\mu_{CA}^{G}$ from SVM-SBF classifier was found to be least (with high $SE_{CA}^{G}$) among the four classifiers for all the datasets (Figure 4 and Figure S4, Document S6). Here, it is pertinent to note that the sigmoid basis function may not be recommended to use in SVM training for real crop GE datasets. Furthermore, similar interpretations can be made for other abiotic (i.e., drought) and biotic (i.e., bacterial, fungal and insect) stress GE datasets (Figure 3 and Figure 4, and Figure S4 and Document S6.

The classification-based performance metrics can be considered as statistically necessary to check the informativeness and robustness of the selected genes. Through such analysis, it was found that the BSM approach performed better in terms of selecting informative and robust genes from the high-dimensional GE data as compared to other competitive methods such as SVM-RFE, MRMR, SVM-MRMR and the information theoretic measures. The reason may be attributed to the inclusion of bootstrap-based subject sampling model along with the self-contained statistical tests, which reduces the spurious association of genes with the target trait as well as with other genes. Further, the performance of BSM approach, in terms of the ability to classify the GE samples, was found to be better as compared to multivariate approaches, i.e., MRMR, SVM-MRMR, univariate approaches, i.e., *t*-test, F-score, Wilcox and informative theoretic measures, i.e., IG and GR. Here, it is worthy to note that the multivariate approaches performed better as compared to the univariate approaches when assessed under classification-based criteria, as the former considers gene-gene associations.

### 3.3. Comparative Performance Analysis Based on QTL Testing

We used publicly available QTL data to statistically measure the biological relevancy of the genes selected through the proposed and existing gene selection method(s). The main rationale behind such analysis is that the genes selected for a stress condition (through a gene selection method) are expected to be overlapped with the stress-specific QTL regions. Therefore, the QTLs and genes selected through these 10 gene selection methods, including the proposed BSM, were mapped to the whole rice genome using an MSU rice genome browser [55]. Further, the list of mapped QTLs for different abiotic (i.e., salinity, cold and drought), and biotic (i.e., bacterial, fungal and insect) stresses in rice along with their chromosomal positions in the genome are given in Supplementary Document S2.

**Figure 5:**
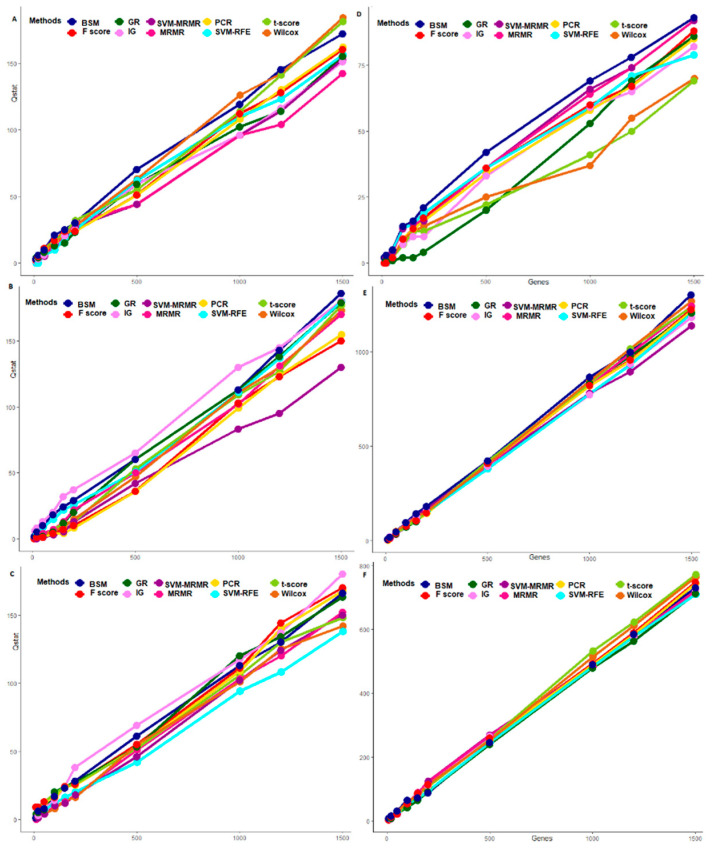
Comparative performance analysis of gene selection methods through distribution of *Qstat* statistic. The horizontal axis represents the informative gene sets obtained through gene selection methods. The vertical axis represents the value of *Qstat* statistic. The distribution of *Qstat* statistic are shown for (**A**) salinity; (**B**) cold; (**C**) drought; (**D**) bacterial; (**E**) fungal and (**F**) insect stress datasets in rice. The lines in different colors represent different gene selection methods.

**Figure 6:**
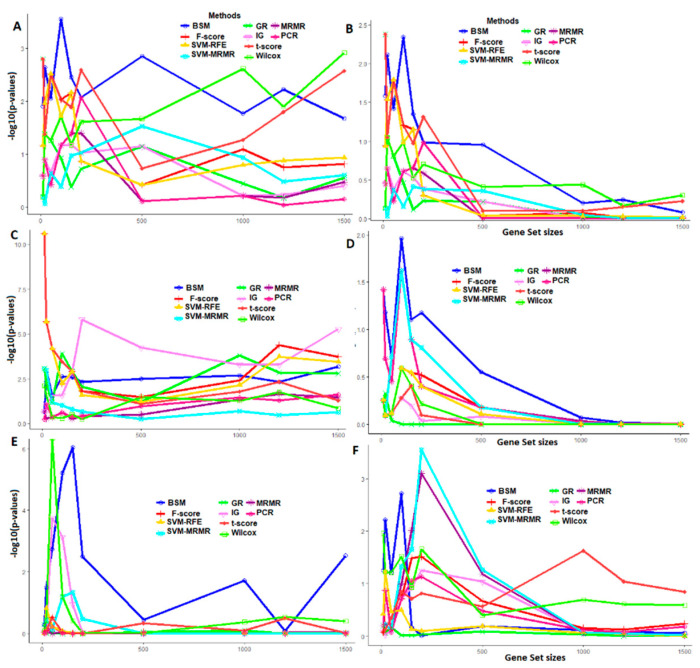
Comparative performance analysis of gene selection methods through distribution of *p*-values from QTL-hypergeometric test. The horizontal axis represents the gene sets obtained through gene selection methods. The vertical axis represents the value of −log10(*p*-value) from QTL-hypergeometric test. The distribution of −log10(*p*-value) are shown for (**A**) salinity; (**B**) cold; (**C**) drought; (**D**) bacterial; (**E**) fungal, and (**F**) insect stress datasets in rice. The lines in different colors represent different gene selection methods.

The biological relevance of the selected genes through both proposed and existing gene selection methods were measured through two criteria, i.e., *Qstat* and −*log*10(*p*-value). The distributions of *Qstat* and −*log*10(*p*-value) over the selected genes for the six different datasets in rice are given in Figure 5 and Figure 6, respectively. For salinity stress data, the values of *Qstat* over all the gene sets of sizes (<1000) selected through the proposed BSM approach were found to be higher than that of SVM-MRMR, SVM-RFE, MRMR, IG, F, Wilcox and PCR (Figure 5A). Further, it may be noted that the proposed approach was equally competitive with the univariate gene selection method such as a *t*-test, while they are assessed in terms of *Qstat* (Figure 5A). For higher gene set sizes (>1000), the value of *Qstat* for Wilcox method was found to be higher than that of proposed and existing approaches (Figure 5A) in the same data. This may be attributed to that the Wilcox method is nonparametric and does not depend on the distribution of GE data. For cold stress data, the values of *Qstat* statistic for all the selected gene sets through the BSM approach were higher than that of other existing methods (Figure 5B). This indicates that the performance of the proposed BSM approach is better in terms selecting cold stress related biologically relevant genes that are mostly overlapped with cold stress QTL regions in rice. Similar interpretations can be made for other abiotic (drought) and biotic (bacterial, fungal and insect) stress datasets in rice (Figure 5). Here, it is worthy to note that the *Qstat* is a linear function of the number of genes selected (through a gene selection approach), number of QTLs reported for that stress and length of the QTL regions (Figure 5).

For salinity stress data, the −*log*10(*p*-value) from hypergeometric test over all the selected gene sets for the proposed BSM approach was observed to be higher than other existing gene selection methods (except t and GR) (Figure 6). In other words, genes selected by the BSM approach were more enriched with the underlying salinity responsive QTLs as compared to other existing methods. Similar interpretations can be made for other abiotic (i.e., cold and drought), and biotic (i.e., bacterial, fungal and insect) stress datasets in rice (Figure 6). Moreover, it is interesting to note that the values of *Qstat* and −*log*10(*p*-value) for (univariate) methods, such as t, F, PCR, Wilcox, IG and GR were found to be higher than that of the existing (multivariate) methods, such as MRMR, SVM-MRMR (Figure 5 and Figure 6). This indicates the better and equally competitive performance of univariate over multivariate methods of gene selection when evaluated through QTL-based biological relevancy criteria. Such observations are not expected in statistics, but are well established in biology through previous studies [56].

Adjudging the performance of gene selection methods based on only classification might lead to the selection of biologically irrelevant genes. Therefore, we used criteria-based on QTLs to test the biological relevancy of the selected genes through proposed, and existing gene selection methods. Through this performance analysis, it was found that the BSM approach selects more biological relevant genes measured in terms of overlapping of the selected genes with given QTL regions as compared to multivariate approaches, i.e., MRMR, SVM-MRMR and machine learning approaches such as SVM-RFE. The proposed BSM approach was equally competitive (and better) with univariate approaches, i.e., *t*-test, F-score, Wilcox and information theoretic measures, i.e., IG and GR, when QTL-based criteria are considered. Through the QTLs-hypergeometric test analysis, it was evident that genes selected through the proposed BSM approach were more statistically enriched with the QTL regions.

### 3.4. Comparative Performance Analysis Based on GO Analysis

The comparative performance analysis of the proposed BSM approach with nine competitive gene selection methods (Document S5) was carried out through GO based distance analysis on six different rice GE datasets. Here, we set *n* (i.e., number of selected genes) as 10, 20, 50, 100, 150, 200 and 500. Then, the selected genes, through the 10 gene selection methods, including the proposed BSM, were annotated with the GO terms under MF, BP and CC categories using *AgriGO* database [46]. The results from this analysis for abiotic stresses under MF, BP and CC GO categories are given in Table 2, Table 3 and Table 4 respectively and for biotic stresses in Supplementary Document S7. For salinity stress data, under the MF category, the values of GO-based average distance scores for the proposed BSM approach were found to be less than that of nine existing methods over all the selected gene sets (Table 2). This indicated that the proposed approach selected more (molecular) functionally similar genes which were responsible salinity tolerance in rice. Similar results can be found for BP and CC GO-based distance analysis for the same stress data (Table 2). In other words, the proposed BSM approach selects more biologically relevant genes attributed to each GO category for salinity stress as compared to the other nine competitive methods (Table 2). For bacterial stress, the values of GO-based average distance score under MF, BP and CC GO categories for the proposed BSM approach were found to be the least among other gene selection methods (Supplementary Document S7). Similar interpretations can be made for other abiotic (i.e., cold and drought) and biotic (i.e., fungal and insect) datasets in rice (Figure 2, Figure 3 and Figure 4, Supplementary Document S7). Through this analysis, it was found that the proposed BSM approach performed better in terms of selecting more functionally relevant genes, which conferred biotic and abiotic stresses tolerance in rice.

The GO-based distance analysis showed that higher functional similarities (which may have biological functions important to stress tolerance) exist among the genes selected by the BSM, as compared to existing methods. The performance of the BSM was found to be better and equally competitive with the univariate approaches, viz. t-score, F-score, Wilcox and correlation-based approaches in terms of selecting genes which are biologically relevant (in terms of GO annotations) for the target trait/condition. It is worthy to note that the univariate approaches performed better as compared to the multivariate approaches under the biology-based criteria, but the former performed poorer than the latter under classification-based criteria. This indicates the real biological complexity for assessing the performance of gene selection approaches on real data. Therefore, we used the comprehensive framework of performance analysis of the gene selection methods under both statistical necessary and biologically relevant criteria. The comparative performance analysis revealed that the proposed BSM approach is better as well as competitive under the classification, QTL and GO-based criteria.

### 3.5. Comparative Performance Analysis Based on Runtime

The recursive feature elimination algorithms-based gene selection methods such as SVM-RFE and SVM-MRMR are computationally intensive and time consuming. So, we used the runtime criterion to evaluate the performance of these gene selection methods. Here, the runtime refers to the amount of computational time required to analyze the GE data through running the codes of the respective methods in R software (v. 4.0.1). The detail results from the runtime-based evaluation of gene selection methods is given in Supplementary Document S8. For bacterial stress GE data (with 8356 genes over 74 samples), SVM-RFE and SVM-MRMR took ~90 and 80 min respectively to analyze on a 2-core DELL PC with 8 GB RAM with Intel(R) Core (TM) i3-6100U CPU at 2.30GHz. On the contrary, the BSM approach took ~25 min to analyze the same GE data to obtain biologically informative genes (Table S20). The BSM method required less computational time than popular methods of gene selection such as SVM-RFE and with much better performance in terms of obtaining biologically informative gene sets. Similar interpretations can be made for the gene selection methods based on the runtime criterion to analyze the remaining five datasets (Tables S21–S23).

## 4. Developed R Software Package

To popularize the use of the proposed gene selection approach among the users, we developed an R software package which includes BSM R package and accompanying documentation with examples. This package is supplied with the manuscript as supplementary information and also available in https://github.com/sam-uofl/BSM. The guidelines for the use of BSM R package is given in Supplementary Document S8. This software is capable of computing weights for gene selection through MRMR, SVM and SVM-MRMR methods, and also provide functions for computing *p*-values and adjusted *p*-values through a BSM approach for different parameter options. Further, it also allows different functions for selecting relevant gene sets through MRMR, SVM, SVM-MRMR and BSM gene selection approaches.

## 5. Conclusions

In GE genomics, the main aim is to select relevant genes which can be used as predictors for the development of statistical/classification models to handle high dimensionality in GE data. Therefore, we proposed an improved BSM statistical approach for gene selection from GE data, which was both effective in reducing redundancy among the genes and improves biological relevancy of genes with the target trait. Here, the genes were selected based on the assessment of the statistical significance of the self-contained null hypothesis under a sound computational framework. Usually, thousand(s) of null hypotheses are tested simultaneously in GE data analysis which increases the chance of selection of false positive genes. Hence, through the proposed BSM approach an adjusted *p*-value was assigned to each gene after multiple test adjustments, and relevant genes were selected based on the adjusted *p*-values. The BSM approach was based on the NP test statistic(s) which does not depend on the distribution of the GE data unlike *t*-test. Further, the bootstrap procedure in the BSM can minimize the redundancy among genes as well as reduce the spurious association of genes with traits during gene selection. The proposed approach was also less computationally expensive compared to SVM-RFE and SVM-MRMR and can be implemented on a personal or workstation computer for analyzing large GE datasets. Furthermore, we used a comprehensive framework of performance analysis of the gene selection methods under statistically necessary and biologically relevant criteria. More specifically, the tested gene selection methods included SVM-RFE from Wrapper, SVM-MRMR and proposed BSM from hybrid (embedded) and the remaining seven from the filter categories. The comparative analysis revealed the proposed approach has the features of an ideal technique of gene selection, as it performed better under both statistically necessary and biologically relevant criteria. Moreover, this study provided a systematic and rigorous evaluation of the gene selection methods under a multi-criteria decision setup on multiple real datasets. It also provided a framework to researchers to comparatively study the available methods, which will guide genome researchers and experimental biologists to select the best method(s) objectively. The proposed approach may provide a statistical template for combing other filter and wrapper gene selection methods under a sound and effective computational environment.

## Figures and Tables

**Table 1:** Rice gene expression datasets used in the study.

Sl. No.	Descriptions	#Series	Series ID	#Genes	#Samples	Stress Type
1.	Salinity stress	3.	GSE14403, GSE16108, GSE6901.	6637	45 (23, 22)	Abiotic
2.	Cold stress	4.	GSE31077, GSE33204. GSE37940, GSE6901.	8840	28 (15, 13)	Abiotic
3.	Drought stress	5.	GSE6901, GSE26280. GSE21651, GSE23211. GSE24048.	9078	70 (35, 35)	Abiotic
4.	Bacterial (xanthomonas) stress	3.	GSE19239, GSE36093. GSE36272.	8356	74 (37, 37)	Biotic
5.	Fungal (blast) stress	2.	GSE41798, GSE7256.	7072	26 (13, 13)	Biotic
6.	Insect (brown plant hopper) stress	1.	GSE29967.	7241	18 (12, 6)	Biotic

**Table 2:** Comparative Performance analysis of the gene selection methods through MF GO-based biologically relevant score for abiotic stresses in rice.

Methods	MRMR	SVM	SVM-MRMR	IG	GR	Wilcox	t	PCR	F	BSM
Salt stress in rice										
10	0.98	0.95	0.97	0.92	0.89	0.93	0.93	0.96	0.96	0.88
20	0.97	0.89	0.93	0.92	0.86	0.89	0.89	0.91	0.91	0.86
50	0.92	0.91	0.92	0.90	0.90	0.87	0.87	0.92	0.92	0.85
100	0.92	0.90	0.89	0.90	0.88	0.87	0.88	0.92	0.91	0.83
150	0.90	0.89	0.90	0.89	0.88	0.87	0.87	0.90	0.91	0.83
200	0.90	0.89	0.88	0.89	0.87	0.88	0.88	0.90	0.90	0.84
500	0.90	0.90	0.89	0.90	0.90	0.89	0.90	0.89	0.89	0.83
Cold stress in rice										
10	0.82	0.84	0.82	0.92	0.99	0.92	0.87	0.77	0.77	0.75
20	0.93	0.88	0.93	0.95	0.93	0.88	0.90	0.91	0.88	0.71
50	0.91	0.88	0.91	0.93	0.90	0.91	0.91	0.92	0.92	0.73
100	0.91	0.90	0.91	0.90	0.88	0.91	0.91	0.91	0.91	0.74
150	0.90	0.89	0.90	0.89	0.89	0.89	0.90	0.91	0.91	0.72
200	0.90	0.89	0.90	0.89	0.88	0.89	0.90	0.90	0.90	0.73
500	0.90	0.88	0.90	0.90	0.89	0.88	0.89	0.88	0.89	0.73
Drought stress in rice										
10	0.82	0.86	0.81	0.90	0.93	0.65	0.76	0.76	0.76	0.71
20	0.79	0.86	0.78	0.91	0.90	0.80	0.81	0.81	0.81	0.75
50	0.88	0.84	0.87	0.88	0.90	0.84	0.88	0.89	0.89	0.75
100	0.89	0.89	0.88	0.89	0.89	0.88	0.88	0.88	0.88	0.76
150	0.88	0.88	0.87	0.89	0.88	0.88	0.88	0.88	0.88	0.76
200	0.88	0.88	0.87	0.88	0.89	0.89	0.88	0.88	0.88	0.74
500	0.88	0.88	0.87	0.88	0.88	0.89	0.88	0.87	0.87	0.73

Values in the last column represent dissimilarity scores obtained from proposed BSM approach.

**Table 3:** Comparative Performance analysis of the gene selection methods through BP GO-based biologically relevant score for abiotic stresses in rice.

Methods	MRMR	SVM	SVM-MRMR	IG	GR	Wilcox	t	PCR	F	BSM
Salt stress in rice										
10	0.86	0.94	0.86	0.92	0.97	0.90	0.90	0.88	0.88	0.83
20	0.90	0.91	0.90	0.89	0.91	0.92	0.92	0.84	0.85	0.84
50	0.89	0.90	0.88	0.88	0.90	0.88	0.89	0.88	0.88	0.82
100	0.88	0.89	0.86	0.89	0.89	0.85	0.86	0.89	0.87	0.82
150	0.87	0.89	0.90	0.88	0.89	0.85	0.85	0.89	0.89	0.83
200	0.87	0.89	0.86	0.88	0.89	0.84	0.85	0.89	0.88	0.82
500	0.87	0.89	0.87	0.87	0.89	0.86	0.86	0.86	0.86	0.82
Cold stress in rice										
10	0.79	0.82	0.79	0.86	0.94	0.91	0.90	0.79	0.79	0.79
20	0.93	0.89	0.93	0.90	0.88	0.86	0.88	0.90	0.86	0.82
50	0.88	0.89	0.88	0.90	0.88	0.88	0.87	0.89	0.90	0.71
100	0.88	0.89	0.88	0.89	0.87	0.90	0.88	0.89	0.89	0.74
150	0.89	0.88	0.89	0.88	0.88	0.88	0.87	0.88	0.88	0.73
200	0.89	0.87	0.89	0.87	0.87	0.87	0.87	0.88	0.84	0.73
500	0.88	0.86	0.88	0.86	0.86	0.84	0.86	0.87	0.83	0.71
Drought stress in rice										
10	0.86	0.79	0.85	0.81	0.89	0.62	0.83	0.83	0.83	0.73
20	0.84	0.79	0.83	0.89	0.90	0.80	0.84	0.84	0.84	0.72
50	0.88	0.81	0.87	0.88	0.88	0.81	0.88	0.88	0.88	0.72
100	0.87	0.84	0.86	0.88	0.88	0.84	0.86	0.87	0.87	0.72
150	0.86	0.84	0.85	0.88	0.88	0.84	0.87	0.87	0.87	0.71
200	0.86	0.84	0.85	0.87	0.87	0.85	0.86	0.86	0.86	0.72
500	0.87	0.85	0.86	0.86	0.87	0.87	0.86	0.85	0.83	0.72

Values in the last column represent dissimilarity scores obtained from proposed BSM approach.

**Table 4:** Comparative Performance analysis of the gene selection methods through CC GO-based biologically relevant score for abiotic stresses in rice.

	MRMR	SVM	SVM-MRMR	IG	GR	Wilcox	t	PCR	F	BSM
Salt stress in rice										
10	0.77	0.71	0.70	0.94	0.97	0.93	0.93	0.95	0.95	0.78
20	0.88	0.87	0.85	0.92	0.90	0.91	0.91	0.88	0.88	0.81
50	0.88	0.89	0.86	0.92	0.92	0.90	0.90	0.89	0.89	0.84
100	0.88	0.90	0.8	0.91	0.89	0.86	0.86	0.88	0.88	0.83
150	0.87	0.90	0.87	0.90	0.89	0.86	0.87	0.88	0.88	0.83
200	0.87	0.89	0.85	0.90	0.90	0.88	0.89	0.88	0.88	0.83
500	0.88	0.90	0.88	0.89	0.90	0.88	0.89	0.87	0.87	0.82
Cold stress in rice										
10	0.78	0.80	0.78	0.96	0.81	0.87	0.86	0.70	0.70	0.70
20	0.88	0.86	0.88	0.96	0.87	0.87	0.89	0.81	0.83	0.71
50	0.86	0.89	0.86	0.90	0.85	0.84	0.85	0.89	0.90	0.73
100	0.88	0.90	0.88	0.90	0.81	0.83	0.84	0.87	0.87	0.74
150	0.88	0.89	0.88	0.90	0.82	0.82	0.86	0.87	0.88	0.74
200	0.87	0.90	0.87	0.90	0.84	0.85	0.86	0.87	0.85	0.73
500	0.88	0.89	0.88	0.89	0.86	0.97	0.86	0.88	0.87	0.73
Drought stress in rice										
10	0.82	0.86	0.81	0.91	0.89	0.83	0.87	0.87	0.87	0.74
20	0.89	0.85	0.88	0.93	0.90	0.87	0.89	0.89	0.89	0.74
50	0.86	0.88	0.85	0.91	0.87	0.87	0.88	0.88	0.88	0.73
100	0.87	0.87	0.86	0.89	0.86	0.87	0.88	0.88	0.88	0.74
150	0.87	0.87	0.86	0.90	0.85	0.85	0.87	0.87	0.87	0.74
200	0.87	0.87	0.86	0.89	0.86	0.86	0.87	0.87	0.87	0.73
500	0.87	0.86	0.86	0.89	0.87	0.88	0.87	0.86	0.85	0.72

Values in the last column represent dissimilarity scores obtained from proposed BSM approach.

## Data Availability

All the secondary data used in this study are available in the NCBI database. The proposed methods are implemented in the developed R package and R codes are freely available at http://github/sam-uofl/BSM.

## Supplementary Materials

The following are available online at https://www.mdpi.com/1099-4300/22/11/1205/s1.
