# Supplementary material for: Statistical Approach for Biologically Relevant Gene Selection from High-Throughput Gene Expression Data
Source: Entropy (Basel). 2020 Oct 25;22(11):1205. doi: 10.3390/e22111205 (PMC7712650; doi:10.3390/e22111205)
Supplement: Supplementary file 1 [file entropy-22-01205-s001.zip › entropy-942531-supplementary/Supplemtary Information.docx]

Document S1. Data Collection, Pre-Processing, Meta-Analysis and Preliminary Gene Selection to Prepare the Data for Performance Analysis of Gene Selection Methods.

Data Selection

The six different gene expression datasets for rice related to a balanced set of three abiotic stresses (salinity, cold and drought) and three biotic stresses (*Xanthomonas* bacteria, fungal and insect) were obtained from Gene Expression Omnibus (GEO) database of NCBI ([http://www.ncbi.nlm.nih.gov](http://www.ncbi.nlm.nih.gov/geo)) with platform GPL2025, as this platform contains as much as 220 microarray experiments (series) comprising 3480 samples/subjects of Oryza sativa L. as compared to other platforms. Among these 3480 samples, 550 experimental samples related to these six different biotic and abiotic stresses for rice were taken in this study. Further, the raw .CEL files for these gene expression samples are downloaded for GEO database of NCBI.

Data Preprocessing

The preprocessing of the gene expression datasets was done to remove noises, including missing and mislabeled probes (Das et al., 2017). Here, the preprocessing of data was conducted by using Bioconductor platform of R (Gentleman et al., 2004). Initially, the raw CEL files of the collected samples were processed using Robust Multichip Average (RMA) algorithm available in *affy* Bioconductor package of R (Gautier et al., 2004; Bolstad et al., 2003). This RMA procedure involves background correction, quantile normalization and summarization by median polish approach. Further, the log2 scale transformed expression data from RMA for the collected experimental samples were used for meta-analysis to remove the outlier samples. After normalization of the data, we used the z-score method, a Location-Scale approach (Lazar et al. 2013) to remove the batch effects of the genes. Here, the main idea behind the use of z-score method is to transform the data from each batch to have similar (equal) z-score (i.e. function of mean and variance) for each gene. It is assumed that these transformations, while trivially making data more comparable, do not remove any biological signal of interest

Meta-analysis of gene expression datasets

Meta-analysis was performed on the collected gene expression samples to remove the unusual or outlier samples from the data. It was performed individually for each stress. Through this, the gene expression samples with mean ≥ *µ_0_* and standard deviation ≤ *σ_0_* are retained and other samples, which do not satisfy this condition are considered as outliers and removed. Further, we validated the homogeneity of the selected samples through correlation analysis, as they were generated over varying experimental conditions. The parameters for meta-analysis in each stress condition are given in Table S2.

**Table S2.** Parameters for gene expression samples selection.

| **Sl. No.** | **Stress condition** | **Mean (*µ_0_*)** | **Standard deviation (*σ_0_*)** | **Samples selected** |
| --- | --- | --- | --- | --- |
| 1 | Salinity stress | 5.7 | 2.6 | 45 |
| 2 | Cold stress | 6.5 | 2.7 | 28 |
| 3 | Drought stress | 6.2 | 2.6 | 70 |
| 4 | Bacterial stress | 6.8 | 2.5 | 74 |
| 5 | Fungal stress | 6.05 | 2.8 | 26 |
| 6 | Insect stress | 7.4 | 2.4 | 18 |


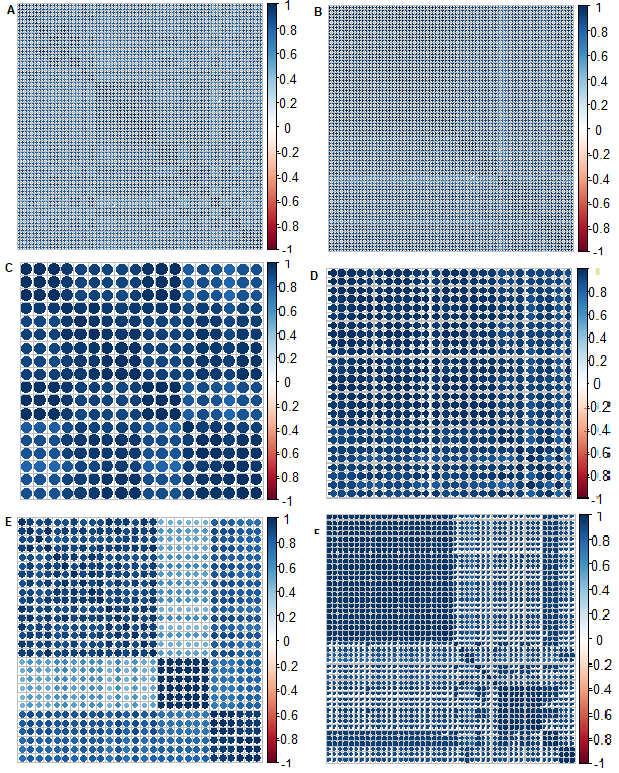


**Figure S1.** Correlation plot of the micro-array experimental samples under salinity stress for rice.

Through this meta-analysis, samples which satisfy the above condition are selected for each stress. These parameter combinations are chosen for each stress, as we observed uniformity of color in the correlation plot at these parameter settings, though they are generated over varying experimental conditions across the globe. For instance, in salinity stress, experimental samples whose mean (*µ*) ≥ 5.23 and standard deviation ≤ 2.51 were retained for further analysis as they are observed to be highly homogeneous at these parameters setting, irrespective of their experimental conditions (Figure S1). Similar interpretations can be made for cold, drought, bacterial, fungal and insect stresses in rice.

Preliminary Gene Selection for Dimension Reduction

The gene expression data generated from Affymetrix Rice Genome Array (GPL 2025 in GEO), which contains 57,381 probes and each probe is assumed to represent an individual gene. Further, there are 123 probe sets designed for control (in GPL 2025), so, we removed these probes from the analysis and the dataset on 57,258 valid probe sets were obtained for further analysis. It would be of high computational complex as well as statistically infeasible to use the gene set selection methods directly on expression data on 57,258 probes. Hence, we first employed t-test and Fold Change (FC) criteria to filter out unlikely genes to reduce the dimension of the GE datasets at the preliminary stage. In our preliminary selection, we assigned 1 and 0.05 as the |FC| and *p-value* thresholds respectively, resulting in selection of several thousands of genes (Table 1). Further, GE data on these selected genes (at the preliminary stage) were further used for performance analysis of proposed and existing gene selection techniques.

References

1. Das S, Meher PK, Rai A, Bhar LM, Mandal BN. Statistical approaches for gene selection, hub gene identification and module interaction in gene co-expression network analysis: An application to Aluminum stress in Soybean (*Glycine max* L.). PLoS ONE. 2017, 12(1): e0169605. doi:10.1371/journal.pone.0169605.
2. Gentleman RC, Carey VJ, Bates DM, Bolstad B, Dettling M, Dudoit S, Ellis B, Gautier L, Ge Y, Gentry J (2004) Bioconductor: open software development for computational biology and bioinformatics. Genome Biol 5: 80.
3. Gautier L, Cope L, Bolstad BM, Irizarry RA (2004) Affy—analysis of Affymetrix GeneChip data at the probe level. Bioinformatics 20: 307–315.
4. Irizarry RA, Hobbs B, Collin F, Beazer-Barclay,YD, Antonellis KJ, Scherf U, Speed TP (2003) Exploration, normalization, and summaries of high density oligonucleotide array probe level data. Biostatistics 4: 249–264.
5. Bolstad B, Irizarry R, Astrand M, Speed T (2003) A comparison of normalization methods for high density oligonucleotide array data based on bias and variance. Bioinformatics 19(2): 185–193.

Document S2: Stress(S) Specific Quantitative Trait Loci Information for Rice (*Oryza Sativa L.*)

The list of trait specific Quantitative Trait Loci (QTL) for the abiotic, *viz*. salinity, drought, and cold, and biotic stresses bacteria (*Xanthomonas*), fungal (blast) and insect (brown plant hopper) for rice were collected from the Gramene QTL database (<http://www.gramene.org/qtl/>) (Ni et al. 2009). Then, the genomic regions of these QTLs (for each stress) were mapped to rice genome using Gramene annotation of rice genome of MSU Rice Genome Annotation (Osa1) Release 6 (Ouyang et al. 2007). For a given QTL, there may be 25–30 genes per cM (∼270 kbp in rice) (Khurana and Gaikwad, 2005). Further, the lists of the QTLs for each of the six different stresses are given in Table S2-S6.

**Table S3.** List of salinity responsive QTLs in rice (*Oryza sativa L.*).

| **Sl. No.** | **QTL ID** | **Chr. No.** | **Start** | **End** | **Note** |
| --- | --- | --- | --- | --- | --- |
| 1 | AQEM001 | 1 | 33956950 | 37713775 |  |
| 2 | AQEM006 | 1 | 9820009 | 11232822 |  |
| 3 | AQGR001 | 1 | 38530957 | 38531467 |  |
| 4 | AQGR002 | 3 | 22798284 | 22830744 |  |
| 5 | AQCL001 | 3 | 484860 | 485333 |  |
| 6 | AQEM009 | 4 | 19928370 | 22355854 |  |
| 7 | AQCL002 | 4 | 33663984 | 33664487 |  |
| 8 | AQCL003 | 5 | 18874932 | 18875558 |  |
| 9 | AQCL004 | 6 | 22862400 | 22862821 |  |
| 10 | AQEM002 | 6 | 21605889 | 24919236 |  |
| 11 | AQEM003 | 7 | 4573316 | 7739951 |  |
| 12 | AQEM004 | 7 | 2633784 | 4575215 |  |
| 13 | AQEM007 | 9 | 14362062 | 17837010 |  |
| 14 | AQEM005 | 1 | 33956950 | 37713775 | Same as 1 |
| 15 | AQEM008 | 7 | 2633784 | 4575215 | Same as 12 |
| 16 | AQEM010 | 7 | 2633784 | 4575215 | Same as 12 |
| 17 | AQEM011 | 7 | 2633784 | 4575215 | Same as 12 |

Sl. No.: Serial number of the QTL; QTL ID: Published qtl id; Chr. No.: Chromosome number of the QTL; Start: start position of the QTL in terms of base pairs (bp); End: end position of the QTL in terms of length of bps.

**Table S4.** List of cold responsive QTLs for rice (*Oryza sativa L.*).

| **Sl. No.** | **QTL ID** | **Chr. No.** | **Start** | **End** | **Note** |
| --- | --- | --- | --- | --- | --- |
| 1 | CQAA8 | 4 | 688353 | 6574518 |  |
| 2 | AQDU008 | 4 | 688353 | 6574518 | Same as 1 |
| 3 | AQDU004 | 11 | 932068 | 932221 |  |
| 4 | CQP8 | 11 | 1491600 | 2523808 |  |
| 5 | AQDU009 | 6 | 5425408 | 5425631 |  |
| 6 | CQAA9 | 6 | 5425408 | 5425631 | Same as 5 |
| 7 | AQAV003 | 1 | 5558576 | 7445919 |  |
| 8 | AQDU014 | 12 | 8826555 | 8826855 |  |
| 9 | AQDU005 | 12 | 8826555 | 8826855 | Same as 8 |
| 10 | AQAV004 | 2 | 11389704 | 12216613 |  |
| 11 | AQAV001 | 2 | 11389704 | 12216613 | Same as 10 |
| 12 | AQAV002 | 9 | 17719660 | 18810331 |  |
| 13 | AQF129 | 8 | 19051713 | 22886866 |  |
| 14 | AQDU002 | 6 | 19499320 | 27252383 |  |
| 15 | AQDU003 | 8 | 20650060 | 21142502 |  |
| 16 | AQDU012 | 8 | 20650060 | 21142502 | Same as 15 |
| 17 | AQDU010 | 8 | 21142348 | 21142502 | Same as 15 |
| 18 | CQAA10 | 8 | 21142348 | 21142502 | Same as 15 |
| 19 | CQP7 | 7 | 22857717 | 22885543 |  |
| 20 | AQDU013 | 11 | 25153466 | 25153681 |  |
| 21 | AQDU015 | 4 | 26857374 | 29061127 |  |
| 22 | AQBO001 | 7 | 27159051 | 27159261 |  |
| 23 | AQAV006 | 5 | 27342022 | 27342124 |  |
| 24 | CQO3 | 4 | 29155838 | 30445683 |  |
| 25 | AQDU001 | 4 | 30772388 | 32650528 |  |
| 26 | CQO1 | 4 | 31276528 | 32772351 |  |
| 27 | CQAA6 | 1 | 32099566 | 33677892 |  |
| 28 | AQDU006 | 1 | 32099566 | 33677892 | Same as 27 |
| 29 | CQP1 | 1 | 34651088 | 39949610 |  |

Sl. No.: Serial number of the QTL; QTL ID: Published qtl id; Chr. No.: Chromosome number of the QTL; Start: start position of the QTL in terms of base pairs (bp); End: end position of the QTL in terms of length of bps.

**Table S5.** List of drought responsive QTLs in rice (*Oryza sativa L.*).

| **Sl. No.** | **QTL ID** | **Chr.** | **Start** | **End** | **Note** |
| --- | --- | --- | --- | --- | --- |
| 1 | CQAI48 | 4 | 13634515 | 13635012 |  |
| 2 | AQA046 | 12 | 26017140 | 27489485 |  |
| 3 | AQA045 | 4 | 13634515 | 13635012 | Same as 1 |
| 4 | CQAI49 | 12 | 26017140 | 27489485 | Same as 2 |
| 5 | AQHP062 | 8 | 21645663 | 21647445 |  |
| 6 | AQHP058 | 2 | 10503368 | 10503846 |  |
| 7 | AQHP059 | 4 | 31662839 | 31663326 |  |
| 8 | AQHP082 | 6 | 6718648 | 9537772 |  |
| 9 | AQHP083 | 7 | 13074864 | 13075056 |  |
| 10 | AQHP070 | 4 | 31662839 | 31663326 | Same as 7 |
| 11 | AQHP065 | 1 | 29184260 | 29184844 |  |
| 12 | AQHP081 | 4 | 8610617 | 8611256 |  |
| 13 | AQHP079 | 3 | 15469002 | 19412007 |  |
| 14 | AQAN005 | 8 | 20094533 | 20094695 |  |
| 15 | AQAN004 | 4 | 8610617 | 8611256 | Same as 12 |
| 16 | AQHP066 | 2 | 29761981 | 29762453 |  |
| 17 | AQHP080 | 4 | 31662839 | 31663326 | Same as 7 |
| 18 | AQHP084 | 8 | 21645663 | 21647445 | Same as 5 |
| 19 | AQHP069 | 3 | 22798284 | 35828040 |  |
| 20 | AQHP068 | 2 | 10503368 | 19866086 |  |
| 21 | AQAN001 | 5 | 27342022 | 28610866 |  |
| 22 | AQHP078 | 11 | 4413928 | 4415836 |  |
| 23 | AQHP067 | 2 | 27034342 | 27035328 |  |
| 24 | AQHP085 | 9 | 20481606 | 20482133 |  |
| 25 | AQHP061 | 6 | 2560318 | 2561213 |  |
| 26 | AQHP075 | 9 | 20481606 | 20482133 | Same as 24 |
| 27 | AQHP060 | 4 | 8610617 | 8611256 | Same as 12 |
| 28 | AQHP074 | 8 | 21645663 | 21647445 | Same as 5 |
| 29 | AQHP087 | 11 | 19565059 | 19565672 |  |

Sl. No.: Serial number of the QTL; QTL ID: Published qtl id; Chr.: Chromosome number of the QTL; Start: start position of the QTL in terms of base pairs (bp); End: end position of the QTL in terms of length of bps.

**Table S6.** List of bacterial responsive QTLs in rice (*Oryza sativa L.*).

| **Sl. No.** | **QTL ID** | **Chr.** | **Start** | **End** | **Note** |
| --- | --- | --- | --- | --- | --- |
| 1 | AQW001 | 2 | 10503368 | 10503846 |  |
| 2 | AQW002 | 3 | 15469002 | 15472481 |  |
| 3 | AQBT021 | 10 | 21098188 | 21099881 |  |
| 4 | AQBT020 | 10 | 21098188 | 21099881 | Same as 3 |
| 5 | AQBT022 | 10 | 21098188 | 21099881 | Same as 3 |
| 6 | AQBT019 | 9 | 22194746 | 22196064 |  |
| 7 | AQBT017 | 9 | 22194746 | 22196064 | Same as 6 |
| 8 | AQBT018 | 9 | 22194746 | 22196064 | Same as 6 |
| 9 | AQBT006 | 3 | 23088332 | 23088721 |  |
| 10 | AQBT007 | 3 | 23088332 | 23088721 | Same as 9 |
| 11 | AQBT005 | 3 | 23088332 | 23088721 | Same as 9 |
| 12 | AQW003 | 3 | 23088332 | 28232092 |  |
| 13 | AQW004 | 5 | 24261900 | 24262670 |  |
| 14 | AQW006 | 8 | 27822512 | 27825271 |  |
| 15 | AQBT023 | 11 | 28409788 | 28412347 |  |
| 16 | AQBT024 | 11 | 28409788 | 28412347 | Same as 16 |
| 17 | AQBT025 | 11 | 28409788 | 28412347 | Same as 16 |
| 18 | AQBT008 | 4 | 31662839 | 31663326 |  |
| 19 | AQBT010 | 4 | 31662839 | 31663326 | Same as 18 |
| 20 | AQBT009 | 4 | 31662839 | 31663326 | Same as 18 |
| 21 | AQBT001 | 2 | 33849901 | 35662199 |  |
| 22 | AQW005 | 7 | 6779215 | 6779821 |  |
| 23 | AQBT029 | 12 | 7729365 | 21041065 |  |
| 24 | AQBT030 | 12 | 7729365 | 21041065 | Same as 23 |
| 25 | AQBT002 | 3 | 718777 | 1450227 |  |
| 26 | AQBT003 | 3 | 718777 | 1450227 | Same as 26 |
| 27 | AQBT004 | 3 | 718777 | 1450227 | Same as 26 |
| 28 | AQBT013 | 4 | 8610617 | 11234543 |  |
| 29 | AQBT012 | 4 | 8610617 | 11234543 | Same as 28 |
| 30 | AQBT011 | 4 | 8610617 | 11234543 | Same as 28 |
| 31 | AQBT014 | 8 | 8923052 | 8924004 |  |
| 32 | AQBT016 | 8 | 8923052 | 8924004 | Same as 31 |
| 33 | AQBT015 | 8 | 8923052 | 8924004 | Same as 31 |

Sl. No.: Serial number of the QTL; QTL ID: Published qtl id; Chr.: Chromosome number of the QTL; Start: start position of the QTL in terms of base pairs (bp); End: end position of the QTL in terms of length of bps.

**Table S7.** List of blast (fungal) responsive unique QTLs in rice (*Oryza sativa L.*).

| **Sl. No.** | **QTL ID** | **Chr.** | **Start** | **Stop** |
| --- | --- | --- | --- | --- |
| 1 | AQAF003 | 1 | 1,98,822 | 18,91,260 |
| 2 | AQEN002 | 1 | 50,94,276 | 1,10,77,990 |
| 3 | AQAH002 | 1 | 2,41,86,290 | 2,91,84,844 |
| 4 | AQEN079 | 1 | 50,94,276 | 50,95,699 |
| 5 | AQGJ001 | 1 | 2,94,46,995 | 2,94,47,853 |
| 6 | AQAF011 | 1 | 3,10,46,003 | 3,10,47,458 |
| 7 | AQAF013 | 1 | 3,30,53,493 | 4,00,65,325 |
| 8 | AQAF015 | 1 | 3,44,70,620 | 4,00,65,325 |
| 9 | AQAF017 | 1 | 3,44,70,620 | 3,77,13,775 |
| 10 | AQAQ008 | 1 | 3,07,37,705 | 4,05,67,354 |
| 11 | AQAF006 | 1 | 74,45,627 | 7445919 |
| 12 | AQEN001 | 1 | 1,46,20,467 | 3,49,40,769 |
| 13 | AQAF007 | 1 | 79,70,722 | 79,70,839 |
| 14 | AQCT001 | 1 | 47,38,488 | 3,01,70,285 |
| 15 | AQEN011 | 1 | 1,46,20,467 | 2,17,01,719 |
| 16 | AQEN051 | 1 | 2,05,98,332 | 2,05,99,810 |
| 17 | AQCT002 | 2 | 3,56,61,689 | 3,56,62,199 |
| 18 | AQAQ001 | 2 | 3,56,61,689 | 3,56,62,199 |
| 19 | AQAF026 | 2 | 3,46,52,316 | 3,51,36,068 |
| 20 | AQEN069 | 2 | 2,74,82,581 | 3,11,07,173 |
| 21 | AQEN070 | 2 | 2,74,82,581 | 27,483,257 |
| 22 | AQEN039 | 3 | 4,84,860 | 4,85,333 |
| 23 | AQEN012 | 3 | 4,84,860 | 1,450,227 |
| 24 | AQEN003 | 3 | 4,84,860 | 34,96,275 |
| 25 | AQAQ020 | 3 | 2,51,28,239 | 25,128,864 |
| 26 | AQAF029 | 3 | 2,30,88,332 | 2,45,95,466 |
| 27 | AQGJ003 | 4 | 86,10,617 | 86,11,256 |
| 28 | AQEN063 | 4 | 3,16,62,839 | 3,16,63,326 |
| 29 | AQAQ015 | 4 | 86,10,617 | 1,12,34,543 |
| 30 | CQAC1 | 4 | 1,99,28,370 | 2,23,55,854 |
| 31 | AQEN061 | 4 | 2,00,87,103 | 2,00,87,362 |
| 32 | AQAQ024 | 5 | 2,25,79,390 | 2,25,80,355 |
| 33 | AQCT003 | 5 | 20,91,276 | 2,782,394 |
| 34 | AQEN041 | 6 | 23,63,670 | 23,63,704 |
| 35 | AQEN005 | 6 | 23,63,670 | 67,20,901 |
| 36 | AQAQ021 | 6 | 2,90,27,995 | 3,09,45,628 |
| 37 | AQEN014 | 6 | 95,36,259 | 2,44,55,212 |
| 38 | AQGJ023 | 6 | 25,60,318 | 62,84,636 |
| 39 | AQEN059 | 6 | 95,36,259 | 95,37,772 |
| 40 | AQGJ008 | 6 | 2,67,07,816 | 26,708,549 |
| 41 | AQCT004 | 6 | 62,83,401 | 6,928,661 |
| 42 | AQAH001 | 6 | 69,27,624 | 6,928,661 |
| 43 | AQEN044 | 7 | 2,94,66,368 | 2,94,67,498 |
| 44 | AQEN007 | 7 | 1,30,74,864 | 29,467,498 |
| 45 | AQAF030 | 7 | 15,36,133 | 2,317,976 |
| 46 | AQEN033 | 7 | 15,36,133 | 1,537,879 |
| 47 | AQAF033 | 7 | 2,54,72,688 | 2,65,29,185 |
| 48 | AQGJ010 | 7 | 2,67,04,922 | 29,467,498 |
| 49 | AQAQ016 | 7 | 1,75,25,817 | 18,686,761 |
| 50 | AQAF031 | 7 | 23,16,691 | 7,232,998 |
| 51 | AQGJ026 | 7 | 71,24,042 | 7,124,718 |
| 52 | AQAF035 | 8 | 41,05,519 | 53,27,118 |
| 53 | AQEN015 | 8 | 41,05,519 | 1,74,38,003 |
| 54 | AQAF034 | 8 | 41,05,519 | 41,06,001 |
| 55 | AQEN037 | 8 | 41,05,519 | 4,106,001 |
| 56 | AQAF038 | 9 | 1,46,48,372 | 20,174,430 |
| 57 | CQAC3 | 9 | 96,29,362 | 10,801,158 |
| 58 | AQAQ022 | 9 | 12,71,123 | 10,801,158 |
| 59 | AQAF040 | 9 | 1,77,19,660 | 18,810,331 |
| 60 | AQAF041 | 9 | 1,99,46,740 | 20,482,185 |
| 61 | AQGJ027 | 9 | 1,88,10,067 | 1,88,10,331 |
| 62 | AQAF042 | 9 | 2,11,89,110 | 22,196,064 |
| 63 | AQCT006 | 10 | 2,09,76,812 | 20,978,165 |
| 64 | AQEN067 | 11 | 2,03,36,572 | 20,337,612 |
| 65 | AQEN016 | 11 | 1,81,78,768 | 20,337,612 |
| 66 | AQAQ017 | 11 | 1,36,71,613 | 28,412,347 |
| 67 | AQEN081 | 11 | 1,81,78,768 | 18,179,510 |
| 68 | AQAO001 | 11 | 1,78,08,335 | 22,816,523 |
| 69 | AQGJ013 | 11 | 66,86,166 | 6,687,145 |
| 70 | AQCT007 | 11 | 46,24,598 | 46,26,888 |
| 71 | AQAQ009 | 12 | 5,32,909 | 1,595,325 |
| 72 | AQEN017 | 12 | 77,29,365 | 23,775,487 |
| 73 | AQEN047 | 12 | 77,29,365 | 7,729,855 |
| 74 | CQAC4 | 12 | 1,10,58,522 | 18,867,702 |
| 75 | AQCT008 | 12 | 77,29,365 | 13,429,507 |
| 76 | AQAQ011 | 12 | 77,29,365 | 77,29,855 |
| 77 | AQEN072 | 12 | 15,94,823 | 15,95,325 |

Sl. No.: Serial number of the unique QTL; QTL ID: Published qtl id; Chr. No.: Chromosome number of the QTL; Start: start position of the QTL in terms of base pairs (bp); End: end position of the QTL in terms of length of bps.

**Table S8.** List of brown plant hopper (insect) responsive unique QTLs in rice (*Oryza sativa L.*).

| **Sl. No.** | **QTL ID** | **Chr.** | **Start** | **Stop** |
| --- | --- | --- | --- | --- |
| 1 | AQAP053 | 2 | 7,44,663 | 7,45,178 |
| 2 | AQAP027 | 6 | 17,64,586 | 18,22,651 |
| 3 | CQAM3 | 1 | 4,29,53,262 | 42,955,596 |
| 4 | AQAP058 | 10 | 53,52,766 | 1,58,02,326 |
| 5 | AQAP009 | 8 | 1,39,27,893 | 2,28,86,866 |
| 6 | AQBA009 | 6 | 67,18,648 | 67,20,901 |
| 7 | AQBA003 | 8 | 2,78,22,512 | 2,78,25,271 |
| 8 | AQBA005 | 4 | 3,36,63,984 | 3,46,98,383 |
| 9 | AQW015 | 12 | 2,74,88,270 | 2,74,89,485 |
| 10 | AQW012 | 11 | 2,31,54,725 | 2,31,55,291 |
| 11 | AQBA002 | 6 | 67,18,648 | 6,720,901 |
| 12 | AQAP023 | 5 | 1,88,74,932 | 22,580,355 |
| 13 | AQAP040 | 6 | 1,70,54,655 | 17,055,184 |
| 14 | AQAP032 | 10 | 2,10,98,188 | 2,10,99,881 |
| 15 | AQAP054 | 3 | 32,36,247 | 3,236,745 |
| 16 | AQAP048 | 4 | 3,06,30,093 | 3,06,30,917 |
| 17 | AQAP036 | 12 | 2,61,07,904 | 26,992,979 |
| 18 | AQAP050 | 11 | 1,36,71,613 | 2,31,55,291 |
| 19 | AQAP051 | 2 | 2,58,65,334 | 27,610,063 |
| 20 | AQAP043 | 5 | 2,25,79,390 | 29,285,656 |
| 21 | AQAP018 | 8 | 2,28,85,196 | 26,282,308 |
| 22 | AQAP035 | 9 | 2,21,94,746 | 2,21,96,064 |
| 23 | AQAP042 | 9 | 2,21,94,746 | 22,196,064 |
| 24 | AQAP029 | 6 | 39,22,784 | 26,708,549 |
| 25 | AQW010 | 6 | 67,18,648 | 19,338,095 |
| 26 | AQBA020 | 6 | 67,18,648 | 1,93,38,095 |
| 27 | AQAP015 | 1 | 2,76,25,475 | 2,91,84,844 |
| 28 | AQAP028 | 8 | 89,23,052 | 8,924,004 |
| 29 | CQAM2 | 6 | 41,60,454 | 8,066,358 |
| 30 | AQW007 | 3 | 57,29,669 | 7,350,653 |
| 31 | AQAP004 | 3 | 3,03,13,472 | 3,03,15,075 |
| 32 | CQT1 | 4 | 35,46,753 | 14,707,274 |
| 33 | AQAP030 | 11 | 1,36,71,613 | 28,412,347 |
| 34 | AQAP045 | 1 | 3,96,87,395 | 4,05,67,354 |
| 35 | AQAP005 | 5 | 52,55,880 | 52,56,140 |
| 36 | AQBA017 | 1 | 1,46,20,467 | 14,626,881 |
| 37 | AQAP056 | 6 | 31,68,314 | 54,25,631 |
| 38 | AQAP057 | 7 | 1,75,25,817 | 2,57,75,868 |
| 39 | AQAP039 | 1 | 10,39,086 | 10,39,868 |
| 40 | AQBA007 | 2 | 1,98,65,083 | 2,45,66,182 |
| 41 | AQAU001 | 2 | 89,84,645 | 18,249,617 |
| 42 | CQT2 | 3 | 3,19,45,962 | 35,710,936 |
| 43 | AQW013 | 12 | 1,96,28,443 | 19,628,925 |
| 44 | AQAP034 | 11 | 57,06,417 | 57,06,935 |
| 45 | AQAP041 | 2 | 45,24,663 | 5,263,536 |
| 46 | AQAP055 | 4 | 2,01,71,917 | 2,01,73,040 |
| 47 | AQW014 | 12 | 1,96,28,443 | 19,628,925 |
| 48 | AQBA022 | 1 | 1,46,20,467 | 14,626,881 |
| 49 | AQW008 | 4 | 2,41,65,104 | 24,165,408 |
| 50 | AQAU003 | 12 | 5,32,909 | 5,33,313 |
| 51 | AQAP007 | 5 | 52,55,880 | 6,700,408 |
| 52 | AQAU002 | 10 | 2,14,02,080 | 23,031,714 |
| 53 | AQAP031 | 8 | 1,39,27,893 | 2,06,50,257 |
| 54 | AQAP052 | 2 | 52,62,891 | 69,16,662 |
| 55 | AQBA008 | 4 | 3,16,62,839 | 32,449,446 |
| 56 | AQAP046 | 10 | 1,77,94,267 | 1,98,23,295 |
| 57 | AQAP001 | 1 | 97,01,793 | 1,04,91,821 |

Sl. No.: Serial number of the unique QTL; QTL ID: Published qtl id; Chr. No.: Chromosome number of the QTL; Start: start position of the QTL in terms of base pairs (bp); End: end position of the QTL in terms of length of bps.

Document S3: Objective Function of Support Vector Machine

Consider $\left\{ \boldsymbol{x}_{m},y_{m} \right\}$ be the GE data for *m^th^* sample (*m =* 1*,* 2*, …, M*). $\left\{ \boldsymbol{x}_{m},y_{m} \right\} \epsilon R^{N}x \{-1, 1\}$ given as input to Support Vector Machine (SVM). Here, we wish to find out a hyperplane that divides the GE samples/subjects, *i.e.* $\boldsymbol{x}_{m}$ for case class ($y_{m}=1)${\displaystyle {\vec {x}}_{i}} from the GE samples/subjects for control class ($y_{m}=-1)$ in such a way that the distance between the hyperplane and the point, *i.e.*{\displaystyle {\vec {x}}_{i}} $\boldsymbol{x}_{m}$ is maximum. Then the hyperplane can be written as:

| $\boldsymbol{k}\boldsymbol{.x}_{\boldsymbol{m}}\boldsymbol{+}b=0$ $\forall m=1, 2,\ldots, M$ | (1) |
| --- | --- |

where, ***k*** and *b* are the weight vector and bias respectively.

Here, we assume that the GE samples for two classes are linearly separable. In other words, we can select two parallel hyperplanes that separate the case and control classes in such a way that the distance between them is maximum.

Now, for case, the hyperplane becomes:

| $\boldsymbol{k}\boldsymbol{.x}_{\boldsymbol{n}}\boldsymbol{+}b=1$ $\forall n=1, 2,\ldots, M_{1}$ | (2) |
| --- | --- |

For control, the hyperplane becomes:

| $\boldsymbol{k}\boldsymbol{.x}_{\boldsymbol{o}}\boldsymbol{+}b=-1$ $\forall o=1, 2,\ldots, M_{2}$ | (3) |
| --- | --- |

Here, weight vector is chosen in such a way that the distance between two the hyperplanes, i.e. Eq. (2) – Eq (3), is maximum. Mathematically:

| $\boldsymbol{k.}\left( \boldsymbol{x}_{\boldsymbol{n}}\boldsymbol{-}\boldsymbol{x}_{\boldsymbol{o}} \right)\boldsymbol{=}2$ | (4) |
| --- | --- |

*Dividing* $\left\| k \right\|^{2}$ in both sides of Eq. 4, it becomes

| $\frac{\boldsymbol{k}}{\left\Vert k \right\Vert^{2}}\boldsymbol{.}\left( \boldsymbol{x}_{\boldsymbol{n}}\boldsymbol{-}\boldsymbol{x}_{\boldsymbol{o}} \right)\boldsymbol{=}\frac{\boldsymbol{2}}{\left\Vert k \right\Vert^{2}}$ | (5) |
| --- | --- |

So, to maximize the distance between the planes in Eq. 5, we need to minimize $\frac{\left\| k \right\|^{2}}{\boldsymbol{2}}$. Hence, the objective function, *J*, for this case vs. control classification problem becomes (Guyon et al., 2003):

| $J =\left\Vert k \right\Vert^{2}/2$ | (6) |
| --- | --- |

Through Taylor series expansion, the objective function, $J,$can be approximated as (at *k*=c):

$$J=\frac{1}{2}\left\{ \left\| c \right\|^{2}+\frac{\partial}{\partial k}{\left\| k \right\|^{2}}_{k=c}\left( k-c \right)+\frac{1}{2!}\frac{\partial^{2}}{\partial k^{2}}{\left\| k \right\|^{2}}_{k=c}\left( k-c \right)^{2}+\frac{1}{3!}{\left\| k \right\|^{2}}_{k=c}\left( k-c \right)^{3}+\ldots\right\}$$

$$=\frac{1}{2}\left\{ \left\| c \right\|^{2}+{\frac{\partial J_{2}}{\partial k}}_{k=c}\left( k-c \right)+\frac{1}{2!}{\frac{\partial^{2}J_{2}}{\partial k^{2}}}_{k=c}\left( k-c \right)^{2}+{\frac{1}{3!}\frac{\partial^{3}J_{2}}{\partial k^{3}}}_{k=c}\left( k-c \right)^{3}+\ldots\right\}$$

Differentiating both sides with respect to *k,* and ignoring the second and higher order derivatives terms from the expression, we have

$$\frac{\partial J}{\partial k}=\frac{1}{2}\left\{ 0+{\frac{\partial J}{\partial k}(\frac{\partial J}{\partial k}}_{k=c}\left( k-c \right)) \right\}$$

$$\frac{\partial J}{\partial k}=\frac{1}{2}\left\{ \left( k-c \right)\frac{\partial^{2}J}{\partial k^{2}}+\frac{\partial J}{\partial k}.1) \right\}$$

$$\frac{\partial J}{\partial k}=\frac{1}{2}\frac{\partial^{2}J}{\partial k^{2}}\left( k-c \right)$$

Replace *k* with $\Delta k$ in above expression and ignoring the constant *c*. Now, the above expression becomes, as below.

$$\lim_{\Delta k} \frac{\Delta J}{\Delta k}=\frac{1}{2}\frac{\partial^{2}J}{\partial k^{2}}\left( \Delta k \right)$$

$$\Delta J=\frac{1}{2}\frac{\partial^{2}J}{\partial k^{2}}\left( \Delta k \right)^{2}$$

Further, $\Delta J$ attributed to *i*^th^ gene can be expressed as:

$$\Delta J(i)=\frac{1}{2}\frac{\partial^{2}J_{2}}{\partial{k_{i}}^{2}}\left( {\Delta k}_{i} \right)^{2}$$

Here, it may be noted that the *J* is directly proportional to ${k_{i}}^{2}$. Hence, measurement of either k_i_ or *J* provides equivalent information. Keeping this in view, ${k_{i}}^{2}$ is used as the ranking criterion for evaluating impact of i^th^ gene on this classification.

Reference

1. Guyon I, Weston J, Barnhill S, Vapnik V. Gene selection for cancer classification using support vector machines. Mach Learn. 2002. doi:10.1023/A:1012487302797

Document S4: Determination of ‘Beta’ for Quadratic Integration

The value of *beta* for computing the score for quadratic integration of SVM with MRMR method was determined empirically from the set {0.1, 0.2, 0.3, 0.4,0.5,0.6, 0.7, 0.8, 0.9}. The classification accuracies were computed based on the fivefold cross-validation performance. Further, the classification accuracies were plotted against beta values to find the optimum value of beta for integrating SVM and MRMR scores through quadratic integration. Here, the above procedure is repeated to obtain optimal beta value for different gene sets, *i.e.* 10, 20, 50, 100, 150 and 200.


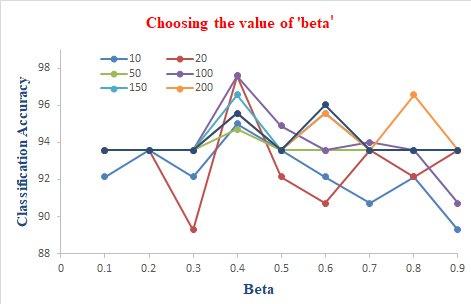


**Figure S2**. Choosing the optimal value of beta for quadratic integration of SVM and MRMR scores. The horizontal axis represents the value of beta. The vertical axis represents the classification accuracy computed over five-fold cross validation through Support Vector Machine-Linear Basis Function classifier. The line plots are shown for selected gene sets with size (A) 10, (B) 20, (C) 50, (D) 100, (E) 150 and (F) 200.

The classification accuracies obtained through training Support Vector Machine-Linear Basis Function classifier over fivefold cross-validations for different beta values are shown in Figure S1. It is found that for gene set of size 10, the maximum classification accuracy is achieved for the beta value of 0.4. Similar observations are found for other gene set of sizes, *e.g.* 20, 50, 100, 150 and 200. Therefore, we can conclude that the optimal value of beta for the quadratic integration of SVM and MRMR scores for the given dataset is 0.4. It may be worthy to note that, the value of beta is highly dependent and need to be estimated from the data itself prior going to final score integration.

Document S5. List of Gene Selection Methods for Performance Analysis.

The comparative performance analysis of the proposed Boot-SVM-MRMR gene selection approach was carried out with respect to nine existing gene selection techniques on multiple (six different) crop gene expression datasets based on different statistically necessary and biological relevant criteria. The list of the methods used for performance analysis are given in Table S9.

**Table S9.** Gene selection methods used in the comparative performance analysis.

| **Methods** | **Expression** | **R tools** | **References** |
| --- | --- | --- | --- |
| BSM | Adjusted *p-value* from BSM | BSM | Proposed |
| SVM-MRMR | $\delta w_{i}+\left( 1-\delta\right){\vert k}_{i}\vert$ | * | [11] |
| MRMR | $w_{i}=\max_{i\in\Omega}\{F\left( i, y \right)/(\frac{1}{N}\sum_{\begin{aligned} j=1 \\ j\neq i \end{aligned}}^{N} \vert C\left( i,j \right)\vert)\}$ | * | [1, 2] |
| SVM-RFE | $k=\sum_{m=1}^{M} \varphi_{m}{y_{m}x.}_{m}$ | e1071 | [3, 4] |
| t-score | $t=\frac{\bar{x_{1}}-\bar{x_{2}}}{\sqrt{S^{2}(\frac{1}{n_{1}}+\frac{1}{n_{2}})}}$ | Mass | [5, 6] |
| F-score | $F=\frac{{(\bar{x_{1}}-\bar{x})}^{2}+{(\bar{x_{2}}-\bar{x})}^{2}}{S_{1}^{2}+S_{2}^{2}}$ | stats | [7] |
| Pearson’s Correlation (PCR) | $r=\frac{S_{12}}{S_{1}S_{2}}$ | FSelector | [8, 9] |
| Information Gain (IG) | $H\left( c \right)+H\left( i \right)-H(c, i)$ | FSelector | [9, 10] |
| Gain Ratio (GR) | ${(H\left( c \right)+H\left( i \right)-H(c, i))}/{H(i)}$ | FSelector | [9, 10] |
| Wilcoxon Statistic (Wilcox) | $\frac{W-\frac{n(n+1)}{4}}{\sqrt{{n(n+1)(2n+1)}/{24}}}$ | Mass | [12] |

Codes: Codes for the methods; ***:** R-code written; BSM: proposed Bootstrap SVM-MRMR gene selection technique; SVM-MRMR: Linear SVM-MRMR; MRMR: Maximum Relevance Minimum Redundancy; SVM-RFE: Support Vector Machine Recursive Feature Elimination; t-score: t-statistic obtained from the t-test; F-score: Fisher’s score; S^2^: pooled variance; S^2^_1_: Sample variance for case group (with class labels as 1); S^2^_2_: Sample variance for control group (with class labels as -1); *H(.)*: Entropy measure

**References**

1. Ding C, Peng H. Minimum redundancy feature selection from microarray gene expression data. J. Bioinformatics Comput Biol. 2005, 3(2):185-205.
2. Peng H, Long F, Ding C. Feature selection based on mutual information: Criteria of max-dependency, max-relevance and min-redundancy. IEEE Trans Pattern Anal Mach Intell. 2005, 27 (8) 1226–1238.
3. Guyon I, Weston J, Barnhill S, Vapnik V. Gene selection for cancer classification using support vector machines. Machine Learning. 2002, 46: 389–422.
4. Liang Y, Zhang F, Wang J, Joshi T, Wang Y, Xu D. Prediction of drought-resistant genes in *Arabidopsis thaliana* using SVM-RFE. PLoS ONE. 2011, 6(7): e21750. doi:10.1371/journal.pone.0021750.
5. Cui X, Churchill G. Statistical tests for differential expression in cDNA microarray experiments. Genome Biol. 2003; 4 (4): 210.
6. Das S, Meher PK, Pradhan UK, Paul AK. Inferring gene regulatory networks using Kendall’s tau correlation coefficient and identification of salinity stress responsive genes in rice. Current Science. 2017b, 112(6): 1257-1262.
7. Lazar C, Taminau J, Meganck S, Steenhoff D, Coletta A, Molter C, et al. A survey on filter techniques for feature selection in gene expression microarray analysis. IEEE/ACM Trans on Comput Biol and Bioinfo. 2012, 9(4): 1106–1119.
8. Golub T, Slonim D, Tamayo P, Huard C, Gaasenbeek M, Mesirov J, et al. Molecular classification of cancer: class discovery and class prediction by gene expression monitoring. Science. 1999, 286: 531-537.
9. Cheng T, Wang Y, Bryant SH. FSelector: a Ruby gem for feature selection. Bioinformatics. 2012, 28(21): 2851–2852. doi: [10.1093/bioinformatics/bts528](http://dx.doi.org/10.1093%2Fbioinformatics%2Fbts528).
10. Liu X, Krishnan A, Mondry A. An entropy-based gene selection method for cancer classification using microarray data. BMC Bioinformatics. 2005, 6:76.
11. Mundra PA, Rajapakse JC. SVM-RFE With MRMR Filter for Gene Selection. IEEE Trans Nanobioscience. 2010;9: 31–37. doi:10.1109/TNB.2009.2035284
12. Hossain A, Willan AR, Beyene J. An Improved Method on Wilcoxon Rank Sum Test for Gene Selection from Microarray Experiments. Commun Stat Simul Comput. 2013, 42 (7): 1563-1577. doi:10.1080/03610918.2012.667479.
13. Lazar, C. et al. Batch effect removal methods for microarray gene expression data integration: a survey, Briefings in Bioinformatics 2013, 14 (4) 469–490. [doi.org/10.1093/bib/bbs037](https://doi.org/10.1093/bib/bbs037)

**Table S10.** Different parameters combination for computation of classification accuracies.

| **Sl. No.** | **Gene set size (*n*)** | **Window size (*S*)** | **Sliding length (*L*)** |
| --- | --- | --- | --- |
| 1 | 10 | 5 | 1 |
| 2 | 20 | 5 | 2 |
| 3 | 50 | 10 | 5 |
| 4 | 100 | 50 | 20 |
| 5 | 150 | 100 | 20 |
| 6 | 200 | 150 | 20 |
| 7 | 500 | 200 | 20 |
| 8 | 1000 | 250 | 20 |
| 9 | 1200 | 300 | 20 |
| 10 | 1500 | 400 | 50 |

Gene set size: size of the gene sets; Window size: number of ranked genes

taken from gene set at each step; Sliding length: length of sliding of

each window at each subsequent step

**Document S6.** Mean Accuracy and Standard Error in Classification Computed through Varying Sliding Windows Size Technique.

**Table S11.** Mean and standard error in classification accuracy computed through varying sliding windows technique for salinity stress in rice.

|  | **Gene sets** | | | | | | | | | |
| --- | --- | --- | --- | --- | --- | --- | --- | --- | --- | --- |
| **Methods** | **10** | **20** | **50** | **100** | **150** | **200** | **500** | **1000** | **1200** | **1500** |
| **SVM-LBF classifier** | | | | | | | | | | |
| **SVM-MRMR** | 97.06 | 96.94 | 96.67 | 96.02 | 95.28 | 95.28 | 95.28 | 95.34 | 95.28 | 95.28 |
|  | 0.44 | 0.36 | 0.41 | 0.74 | 0.56 | 0.54 | 0.67 | 0.85 | 0.56 | 0.46 |
| **MRMR** | 96.56 | 96.17 | 95.89 | 94.78 | 94.78 | 94.78 | 94.78 | 94.89 | 94.78 | 94.78 |
|  | 0.44 | 0.41 | 0.42 | 0.46 | 0.42 | 0.39 | 0.23 | 0.47 | 0.57 | 0.59 |
| **SVM-RFE** | 97.56 | 97.72 | 96.33 | 95.04 | 95.04 | 95.78 | 96.22 | 96.60 | 96.67 | 96.69 |
|  | 0.44 | 0.28 | 0.56 | 0.74 | 0.74 | 0.23 | 0.24 | 0.18 | 0.16 | 0.23 |
| **Inf. Gain** | 95.00 | 95.00 | 94.17 | 92.78 | 92.04 | 92.78 | 92.78 | 92.72 | 92.73 | 92.68 |
|  | 0.46 | 0.59 | 0.41 | 0.45 | 0.74 | 0.56 | 0.23 | 0.32 | 0.49 | 0.30 |
| **Wilcoxon test** | 92.67 | 93.44 | 92.89 | 91.78 | 91.78 | 91.78 | 91.63 | 91.25 | 91.58 | 91.37 |
|  | 0.54 | 0.36 | 0.42 | 0.45 | 0.57 | 0.32 | 0.48 | 0.43 | 0.49 | 0.44 |
| **t-test** | 92.56 | 93.00 | 92.17 | 90.04 | 90.78 | 90.78 | 90.48 | 90.49 | 90.33 | 90.27 |
|  | 0.61 | 0.45 | 0.41 | 0.74 | 0.48 | 0.55 | 0.89 | 0.87 | 0.71 | 0.57 |
| **PCR** | 91.67 | 92.72 | 91.89 | 90.78 | 90.78 | 90.78 | 90.78 | 90.78 | 90.78 | 90.78 |
|  | 0.54 | 0.28 | 0.56 | 0.56 | 0.66 | 0.48 | 0.66 | 0.81 | 0.47 | 0.57 |
| **F-Score** | 91.67 | 92.72 | 92.17 | 90.78 | 90.78 | 90.78 | 90.78 | 90.78 | 90.78 | 90.78 |
|  | 0.54 | 0.28 | 0.41 | 0.53 | 0.58 | 0.52 | 0.31 | 0.51 | 0.58 | 0.51 |
| **Gain Ratio** | 93.00 | 93.00 | 93.00 | 91.52 | 90.78 | 90.04 | 89.15 | 90.08 | 90.23 | 90.37 |
|  | 0.77 | 0.74 | 0.74 | 0.74 | 0.74 | 0.74 | 0.26 | 0.17 | 0.14 | 0.19 |
| **BSM** | 98.00 | 98.00 | 98.33 | 95.78 | 95.78 | 97.78 | 95.78 | 95.84 | 97.88 | 95.58 |
|  | 0.32 | 0.30 | 0.56 | 0.28 | 0.16 | 0.14 | 0.12 | 0.06 | 0.07 | 0.14 |
| **SVM-PBF classifier** | | | | | | | | | | |
| **SVM-MRMR** | 95.56 | 95.17 | 94.61 | 94.52 | 93.78 | 93.78 | 93.78 | 93.84 | 93.78 | 93.78 |
|  | 0.44 | 0.36 | 0.41 | 0.74 | 0.56 | 0.54 | 0.67 | 0.85 | 0.56 | 0.46 |
| **MRMR** | 93.11 | 93.17 | 93.44 | 91.78 | 91.78 | 91.78 | 91.78 | 91.84 | 91.63 | 91.07 |
|  | 0.44 | 0.41 | 0.42 | 0.46 | 0.42 | 0.39 | 0.23 | 0.47 | 0.57 | 0.59 |
| **SVM-RFE** | 94.56 | 94.72 | 93.61 | 92.04 | 92.04 | 92.78 | 92.78 | 93.30 | 93.32 | 93.69 |
|  | 0.44 | 0.28 | 0.56 | 0.74 | 0.74 | 0.23 | 0.24 | 0.18 | 0.16 | 0.23 |
| **Inf. Gain** | 93.00 | 93.00 | 92.17 | 90.78 | 90.78 | 90.78 | 90.78 | 90.66 | 90.73 | 90.78 |
|  | 0.46 | 0.59 | 0.41 | 0.45 | 0.74 | 0.56 | 0.23 | 0.32 | 0.49 | 0.30 |
| **Wilcoxon test** | 91.67 | 92.72 | 91.33 | 90.78 | 90.78 | 90.78 | 90.33 | 89.20 | 89.25 | 89.06 |
|  | 0.54 | 0.36 | 0.42 | 0.45 | 0.57 | 0.32 | 0.48 | 0.43 | 0.49 | 0.44 |
| **t-test** | 92.56 | 92.44 | 91.89 | 90.78 | 89.30 | 90.78 | 90.48 | 90.37 | 90.23 | 89.77 |
|  | 0.61 | 0.45 | 0.41 | 0.74 | 0.48 | 0.55 | 0.89 | 0.87 | 0.71 | 0.57 |
| **PCR** | 91.22 | 92.44 | 91.61 | 90.78 | 89.30 | 90.78 | 90.33 | 90.78 | 90.78 | 90.78 |
|  | 0.54 | 0.28 | 0.56 | 0.56 | 0.66 | 0.48 | 0.66 | 0.81 | 0.47 | 0.57 |
| **F-Score** | 91.22 | 92.44 | 92.17 | 90.78 | 90.78 | 90.78 | 90.78 | 90.78 | 90.78 | 90.68 |
|  | 0.54 | 0.28 | 0.41 | 0.53 | 0.58 | 0.52 | 0.31 | 0.51 | 0.58 | 0.51 |
| **Gain Ratio** | 93.00 | 93.00 | 93.00 | 91.52 | 90.78 | 90.04 | 89.30 | 90.08 | 90.09 | 90.37 |
|  | 0.77 | 0.74 | 0.74 | 0.74 | 0.74 | 0.74 | 0.26 | 0.17 | 0.14 | 0.19 |
| **BSM** | 98.56 | 98.72 | 98.61 | 97.04 | 97.78 | 97.04 | 97.78 | 97.84 | 97.73 | 97.07 |
|  | 0.32 | 0.30 | 0.56 | 0.28 | 0.16 | 0.14 | 0.12 | 0.06 | 0.07 | 0.14 |
| **SVM-RBF classifier** | | | | | | | | | | |
| **SVM-MRMR** | 95.26 | 94.59 | 94.31 | 93.48 | 93.48 | 93.48 | 93.48 | 93.54 | 93.48 | 93.48 |
|  | 0.44 | 0.42 | 0.41 | 0.66 | 0.45 | 0.52 | 0.40 | 0.37 | 0.26 | 0.20 |
| **MRMR** | 93.56 | 92.89 | 92.89 | 91.78 | 91.78 | 91.78 | 91.78 | 91.84 | 91.68 | 91.78 |
|  | 0.44 | 0.42 | 0.66 | 0.56 | 0.47 | 0.50 | 0.57 | 0.58 | 0.65 | 0.26 |
| **SVM-RFE** | 93.11 | 94.39 | 93.28 | 94.30 | 94.30 | 93.56 | 94.48 | 94.72 | 94.63 | 94.68 |
|  | 0.44 | 0.72 | 0.28 | 0.74 | 0.74 | 0.00 | 0.20 | 0.06 | 0.11 | 0.18 |
| **Inf. Gain** | 90.78 | 90.50 | 89.39 | 90.04 | 89.30 | 90.78 | 89.89 | 89.37 | 89.74 | 90.07 |
|  | 0.00 | 0.50 | 0.58 | 0.74 | 0.74 | 0.00 | 0.29 | 0.23 | 0.18 | 0.23 |
| **Wilcoxon test** | 91.22 | 92.17 | 90.22 | 90.78 | 90.04 | 90.78 | 89.30 | 90.02 | 90.19 | 90.37 |
|  | 0.44 | 0.41 | 0.56 | 0.00 | 0.74 | 0.00 | 0.35 | 0.17 | 0.15 | 0.19 |
| **t-test** | 92.11 | 92.72 | 91.89 | 90.78 | 90.78 | 90.78 | 89.89 | 89.55 | 89.40 | 89.16 |
|  | 0.54 | 0.28 | 0.42 | 0.00 | 0.00 | 0.00 | 0.29 | 0.18 | 0.16 | 0.26 |
| **PCR** | 91.22 | 92.44 | 91.06 | 90.78 | 90.78 | 90.78 | 90.63 | 90.78 | 90.78 | 90.78 |
|  | 0.44 | 0.36 | 0.66 | 0.00 | 0.00 | 0.00 | 0.15 | 0.00 | 0.00 | 0.00 |
| **F-Score** | 91.22 | 92.44 | 91.89 | 90.78 | 90.78 | 90.04 | 90.48 | 90.72 | 90.73 | 90.78 |
|  | 0.44 | 0.36 | 0.59 | 0.00 | 0.00 | 0.74 | 0.20 | 0.06 | 0.05 | 0.00 |
| **Gain Ratio** | 91.22 | 91.89 | 92.17 | 89.30 | 90.04 | 90.78 | 89.44 | 89.84 | 90.04 | 89.87 |
|  | 0.44 | 0.42 | 0.41 | 0.74 | 0.74 | 0.00 | 0.29 | 0.18 | 0.16 | 0.24 |
| **BSM** | 97.11 | 97.17 | 94.39 | 93.56 | 93.56 | 93.56 | 94.44 | 95.25 | 95.28 | 95.37 |
|  | 0.54 | 0.58 | 0.41 | 0.00 | 0.00 | 0.00 | 0.29 | 0.16 | 0.14 | 0.19 |
| **SVM-SBF classifier** | | | | | | | | | | |
| **SVM-MRMR** | 35.78 | 39.89 | 37.94 | 40.81 | 40.07 | 40.07 | 40.22 | 38.46 | 39.58 | 41.76 |
|  | 2.29 | 1.77 | 1.62 | 1.48 | 1.48 | 1.48 | 1.08 | 1.02 | 0.88 | 1.25 |
| **MRMR** | 31.56 | 34.17 | 34.17 | 30.37 | 32.59 | 34.07 | 32.44 | 34.15 | 33.78 | 33.94 |
|  | 0.83 | 2.29 | 1.39 | 1.28 | 1.28 | 4.12 | 1.29 | 0.76 | 0.61 | 0.96 |
| **SVM** | 35.11 | 38.33 | 40.00 | 42.22 | 42.22 | 38.52 | 39.56 | 43.45 | 39.95 | 38.59 |
|  | 2.57 | 1.45 | 2.59 | 1.48 | 3.23 | 0.74 | 1.48 | 0.63 | 0.88 | 0.95 |
| **Inf. Gain** | 31.56 | 31.11 | 31.39 | 28.89 | 34.81 | 34.81 | 35.41 | 34.33 | 33.88 | 33.43 |
|  | 2.37 | 1.51 | 1.75 | 1.28 | 3.92 | 2.96 | 1.00 | 0.87 | 0.87 | 1.51 |
| **Wilcoxon test** | 32.44 | 30.83 | 35.00 | 32.59 | 34.07 | 31.85 | 35.70 | 32.28 | 33.09 | 33.64 |
|  | 1.51 | 1.29 | 1.38 | 3.92 | 5.79 | 0.74 | 1.66 | 0.91 | 0.99 | 1.02 |
| **t-test** | 30.67 | 33.33 | 36.67 | 35.56 | 35.56 | 32.59 | 34.22 | 33.86 | 33.78 | 33.13 |
|  | 1.30 | 1.78 | 1.88 | 4.63 | 4.44 | 1.96 | 1.72 | 0.92 | 0.88 | 0.91 |
| **Lin. Correlation** | 30.67 | 30.28 | 35.56 | 31.85 | 32.59 | 33.33 | 36.44 | 34.15 | 34.47 | 34.34 |
|  | 1.78 | 1.92 | 1.57 | 4.12 | 0.74 | 2.57 | 1.88 | 0.98 | 0.84 | 1.27 |
| **F-Score** | 33.78 | 35.00 | 35.00 | 28.15 | 34.81 | 37.04 | 33.93 | 33.16 | 35.46 | 35.05 |
|  | 5.04 | 2.29 | 2.13 | 2.96 | 2.67 | 1.96 | 1.09 | 0.89 | 0.81 | 1.15 |
| **Gain Ratio** | 37.78 | 34.72 | 34.44 | 37.04 | 32.59 | 36.30 | 32.89 | 33.80 | 32.64 | 34.14 |
|  | 2.53 | 1.78 | 1.39 | 1.48 | 3.23 | 3.23 | 1.43 | 0.78 | 0.68 | 0.98 |
| **BSM** | 41.83 | 39.17 | 40.28 | 40.83 | 40.09 | 39.35 | 40.09 | 41.83 | 41.62 | 40.43 |
|  | 3.25 | 2.44 | 2.25 | 1.28 | 1.96 | 3.23 | 1.40 | 0.73 | 0.75 | 1.12 |

For each method, the first row represents the value of mean CA and second row represents the value of standard error in CA

**Table S12.** Mean and standard error in classification accuracy computed through varying sliding windows technique for cold stress in rice.

|  | **Gene sets** | | | | | | | | | |
| --- | --- | --- | --- | --- | --- | --- | --- | --- | --- | --- |
| **Methods** | **10** | **20** | **50** | **100** | **150** | **200** | **500** | **1000** | **1200** | **1500** |
| **SVM-LBF classifier** | | | | | | | | | | |
| **SVM-MRMR** | 95.29 | 96.00 | 95.55 | 94.81 | 96.00 | 96.00 | 94.10 | 94.59 | 93.78 | 91.94 |
|  | 0.71 | 0.86 | 0.81 | 1.19 | 1.05 | 1.15 | 0.59 | 0.32 | 0.46 | 0.82 |
| **MRMR** | 95.00 | 94.55 | 93.66 | 93.81 | 95.00 | 95.00 | 91.90 | 90.96 | 91.98 | 89.32 |
|  | 0.00 | 0.45 | 0.65 | 1.19 | 1.48 | 1.76 | 1.09 | 0.57 | 0.45 | 1.34 |
| **SVM-RFE** | 93.57 | 96.88 | 97.77 | 95.00 | 94.66 | 95.00 | 95.00 | 95.00 | 95.00 | 94.51 |
|  | 1.34 | 1.05 | 0.94 | 0.72 | 0.96 | 0.90 | 0.93 | 0.63 | 0.72 | 0.49 |
| **Inf. Gain** | 93.00 | 93.00 | 93.00 | 93.00 | 91.81 | 93.00 | 87.05 | 85.95 | 85.54 | 86.67 |
|  | 1.90 | 1.48 | 1.18 | 1.76 | 1.62 | 1.66 | 1.66 | 0.71 | 0.62 | 0.66 |
| **Wilcoxon test** | 93.00 | 91.66 | 90.77 | 85.86 | 88.10 | 86.90 | 85.24 | 83.46 | 82.06 | 80.84 |
|  | 0.00 | 0.65 | 0.94 | 2.06 | 2.38 | 1.19 | 0.77 | 0.76 | 0.76 | 0.98 |
| **t-test** | 91.57 | 91.66 | 89.43 | 87.05 | 85.29 | 82.90 | 82.90 | 79.08 | 78.38 | 75.22 |
|  | 0.87 | 0.65 | 1.17 | 3.15 | 2.06 | 1.19 | 0.57 | 0.61 | 0.53 | 1.19 |
| **PCR** | 92.29 | 92.11 | 91.66 | 90.62 | 91.81 | 91.81 | 90.14 | 87.83 | 86.89 | 86.67 |
|  | 0.71 | 0.58 | 0.94 | 2.38 | 1.19 | 1.19 | 0.71 | 0.73 | 0.75 | 0.91 |
| **F-Score** | 91.57 | 92.11 | 91.66 | 91.81 | 91.81 | 90.62 | 89.90 | 87.92 | 87.84 | 86.34 |
|  | 0.87 | 0.58 | 0.65 | 1.19 | 1.19 | 1.19 | 0.91 | 0.55 | 0.62 | 0.79 |
| **Gain Ratio** | 93.00 | 93.00 | 92.55 | 78.71 | 78.71 | 84.67 | 88.00 | 86.23 | 85.86 | 87.64 |
|  | 2.71 | 2.49 | 2.45 | 4.12 | 2.06 | 2.38 | 1.25 | 0.61 | 0.72 | 0.99 |
| **BSM** | 95.00 | 98.21 | 97.65 | 97.65 | 97.65 | 97.65 | 97.65 | 97.56 | 96.54 | 96.33 |
|  | 1.67 | 0.95 | 0.45 | 0.40 | 0.32 | 0.35 | 0.30 | 0.24 | 0.21 | 0.20 |
| **SVM-PBF classifier** | | | | | | | | | | |
| **SVM-MRMR** | 95.29 | 96.00 | 95.11 | 94.81 | 96.00 | 94.81 | 93.38 | 94.03 | 93.86 | 92.27 |
|  | 0.71 | 1.76 | 0.58 | 1.48 | 1.19 | 1.19 | 0.74 | 0.35 | 0.42 | 0.98 |
| **MRMR** | 94.29 | 95.00 | 93.66 | 92.62 | 93.81 | 95.00 | 92.14 | 90.77 | 89.92 | 87.53 |
|  | 0.71 | 1.76 | 0.65 | 1.19 | 1.19 | 1.59 | 0.87 | 0.63 | 0.69 | 1.43 |
| **SVM-RFE** | 94.29 | 93.98 | 95.32 | 93.00 | 93.00 | 93.62 | 92.00 | 94.91 | 94.92 | 91.00 |
|  | 0.87 | 1.42 | 1.75 | 2.38 | 2.38 | 2.38 | 0.98 | 0.94 | 0.79 | 0.74 |
| **Inf. Gain** | 93.00 | 93.00 | 93.00 | 93.00 | 93.00 | 93.00 | 87.29 | 86.05 | 84.43 | 84.72 |
|  | 1.86 | 1.86 | 1.86 | 1.86 | 1.86 | 1.86 | 1.43 | 0.76 | 0.77 | 1.01 |
| **Wilcoxon test** | 92.29 | 90.77 | 90.77 | 83.48 | 82.29 | 79.90 | 77.29 | 77.02 | 76.02 | 74.66 |
|  | 0.71 | 0.94 | 0.65 | 4.29 | 2.06 | 1.19 | 0.68 | 0.60 | 0.66 | 0.98 |
| **t-test** | 89.43 | 89.88 | 89.43 | 87.05 | 79.90 | 79.90 | 81.81 | 78.15 | 77.52 | 75.14 |
|  | 0.67 | 0.45 | 0.67 | 3.15 | 1.19 | 1.19 | 0.84 | 0.56 | 0.51 | 0.91 |
| **PCR** | 90.14 | 91.21 | 90.77 | 93.00 | 93.00 | 91.81 | 89.67 | 87.55 | 86.89 | 86.02 |
|  | 0.71 | 0.67 | 0.94 | 1.48 | 1.62 | 1.19 | 0.74 | 0.72 | 0.65 | 0.68 |
| **F-Score** | 90.14 | 91.21 | 89.88 | 93.00 | 93.00 | 89.43 | 89.19 | 87.55 | 87.37 | 85.53 |
|  | 0.71 | 0.67 | 1.05 | 1.36 | 1.14 | 3.57 | 0.55 | 0.70 | 0.58 | 1.12 |
| **Gain Ratio** | 93.00 | 92.55 | 91.21 | 81.10 | 84.67 | 83.48 | 86.81 | 85.95 | 85.54 | 86.83 |
|  | 1.98 | 0.95 | 0.99 | 4.29 | 1.19 | 1.19 | 0.81 | 0.77 | 0.76 | 0.75 |
| **BSM** | 97.07 | 97.61 | 98.05 | 97.31 | 97.00 | 95.81 | 96.29 | 96.81 | 96.21 | 96.68 |
|  | 1.29 | 0.58 | 0.45 | 1.19 | 0.75 | 0.90 | 0.38 | 0.13 | 0.30 | 0.22 |
| **SVM-RBF classifier** | | | | | | | | | | |
| **SVM-MRMR** | 96.00 | 96.00 | 94.21 | 94.81 | 93.62 | 96.00 | 94.33 | 94.31 | 93.70 | 92.59 |
|  | 1.90 | 1.48 | 0.95 | 1.19 | 1.19 | 0.78 | 0.59 | 0.32 | 0.43 | 0.95 |
| **MRMR** | 95.00 | 94.55 | 94.11 | 92.62 | 92.62 | 93.81 | 91.19 | 91.33 | 89.37 | 89.48 |
|  | 0.96 | 0.45 | 0.58 | 1.19 | 1.19 | 1.19 | 1.18 | 0.55 | 0.84 | 1.14 |
| **SVM-RFE** | 93.57 | 94.64 | 95.54 | 95.24 | 96.43 | 96.43 | 96.43 | 95.77 | 94.84 | 95.13 |
|  | 0.71 | 1.17 | 1.12 | 1.19 | 1.48 | 1.62 | 0.35 | 0.33 | 0.43 | 0.76 |
| **Inf. Gain** | 93.00 | 92.11 | 91.21 | 87.05 | 87.05 | 89.43 | 84.43 | 77.40 | 76.57 | 76.28 |
|  | 0.85 | 0.58 | 0.67 | 1.19 | 1.76 | 1.62 | 1.55 | 1.47 | 1.18 | 2.17 |
| **Wilcoxon test** | 93.00 | 91.66 | 89.88 | 88.24 | 83.48 | 82.29 | 79.67 | 77.30 | 75.30 | 75.31 |
|  | 0.94 | 0.94 | 1.05 | 1.19 | 1.19 | 1.48 | 0.89 | 0.76 | 0.86 | 1.38 |
| **t-test** | 90.86 | 89.88 | 88.09 | 84.67 | 81.10 | 79.90 | 79.43 | 79.75 | 78.40 | 75.14 |
|  | 0.87 | 0.45 | 0.94 | 1.19 | 1.19 | 1.19 | 0.94 | 0.40 | 0.59 | 1.29 |
| **PCR** | 91.57 | 90.77 | 91.21 | 91.81 | 93.00 | 91.81 | 89.67 | 87.36 | 87.37 | 86.67 |
|  | 0.87 | 0.65 | 0.95 | 1.19 | 1.48 | 1.19 | 0.95 | 0.58 | 0.50 | 0.88 |
| **F-Score** | 90.86 | 90.77 | 91.66 | 91.81 | 93.00 | 91.81 | 89.90 | 87.45 | 87.05 | 85.53 |
|  | 1.43 | 0.65 | 0.65 | 1.19 | 1.76 | 1.19 | 0.48 | 0.60 | 0.53 | 0.74 |
| **Gain Ratio** | 88.00 | 88.98 | 83.18 | 70.38 | 75.14 | 78.71 | 82.29 | 74.58 | 77.37 | 79.85 |
|  | 0.87 | 1.05 | 1.31 | 2.38 | 2.81 | 2.06 | 1.39 | 1.50 | 1.18 | 1.73 |
| **BSM** | 93.57 | 96.88 | 97.77 | 96.43 | 96.43 | 98.81 | 97.38 | 95.96 | 95.32 | 95.45 |
|  | 1.34 | 1.05 | 0.65 | 1.06 | 0.62 | 1.19 | 0.74 | 0.47 | 0.42 | 0.48 |
| **SVM-SBF classifier** | | | | | | | | | | |
| **SVM-MRMR** | 35.00 | 44.64 | 43.30 | 45.24 | 33.33 | 40.48 | 39.76 | 38.06 | 38.17 | 41.07 |
|  | 4.43 | 2.94 | 2.18 | 1.19 | 5.19 | 8.33 | 1.90 | 1.21 | 1.45 | 1.46 |
| **MRMR** | 35.00 | 43.30 | 41.07 | 46.43 | 46.43 | 39.29 | 42.38 | 41.54 | 40.56 | 43.34 |
|  | 2.08 | 2.65 | 3.51 | 7.14 | 3.57 | 3.57 | 1.77 | 1.26 | 0.88 | 1.70 |
| **SVM-RFE** | 45.00 | 36.61 | 40.18 | 50.00 | 39.29 | 44.05 | 40.71 | 39.19 | 39.84 | 37.18 |
|  | 4.01 | 3.22 | 3.15 | 3.57 | 3.57 | 5.19 | 1.77 | 1.47 | 1.06 | 2.20 |
| **Inf. Gain** | 34.29 | 45.98 | 41.96 | 39.29 | 40.48 | 33.33 | 38.81 | 39.29 | 40.24 | 37.82 |
|  | 2.14 | 2.38 | 2.50 | 3.57 | 4.29 | 6.30 | 2.00 | 1.35 | 1.43 | 1.93 |
| **Wilcoxon test** | 45.71 | 40.18 | 38.39 | 40.48 | 42.86 | 36.90 | 39.76 | 40.13 | 39.44 | 39.45 |
|  | 2.08 | 3.36 | 3.00 | 4.29 | 0.00 | 3.15 | 2.00 | 1.50 | 1.38 | 1.67 |
| **t-test** | 40.71 | 46.88 | 42.86 | 36.90 | 47.62 | 42.86 | 40.00 | 40.32 | 37.14 | 40.10 |
|  | 4.16 | 1.96 | 3.17 | 5.19 | 3.15 | 6.19 | 1.40 | 1.59 | 1.34 | 1.77 |
| **PCR** | 40.00 | 39.29 | 38.39 | 39.29 | 38.10 | 32.14 | 36.19 | 41.26 | 41.59 | 38.80 |
|  | 2.62 | 2.13 | 3.15 | 9.45 | 3.15 | 5.46 | 2.46 | 1.35 | 1.30 | 1.55 |
| **F-Score** | 40.71 | 38.84 | 37.50 | 40.48 | 39.29 | 46.43 | 36.90 | 39.94 | 40.08 | 42.53 |
|  | 4.01 | 3.47 | 1.79 | 4.29 | 7.14 | 3.57 | 1.58 | 1.56 | 1.28 | 1.39 |
| **Gain Ratio** | 41.43 | 40.63 | 36.16 | 46.43 | 40.48 | 42.86 | 38.57 | 38.63 | 38.81 | 37.34 |
|  | 3.68 | 2.52 | 2.82 | 3.57 | 4.29 | 6.19 | 1.78 | 1.39 | 1.33 | 1.50 |
| **BSM** | 40.86 | 44.52 | 39.61 | 42.29 | 39.90 | 42.29 | 43.24 | 44.07 | 43.24 | 41.47 |
|  | 3.50 | 2.43 | 3.00 | 3.57 | 3.15 | 3.57 | 2.27 | 1.43 | 1.11 | 1.41 |

For each method, the first row represents the value of mean CA and second row represents the value of standard error in CA

**Table S13.** Mean and standard error in classification accuracy computed through varying sliding windows technique for drought stress in rice.

|  | **Gene sets** | | | | | | | | | |
| --- | --- | --- | --- | --- | --- | --- | --- | --- | --- | --- |
| **Methods** | **10** | **20** | **50** | **100** | **150** | **200** | **500** | **1000** | **1200** | **1500** |
| **SVM-LBF classifier** | | | | | | | | | | |
| **SVM-MRMR** | 95.57 | 95.93 | 95.57 | 95.57 | 95.57 | 95.57 | 95.57 | 95.57 | 95.51 | 95.57 |
|  | 0.88 | 0.71 | 0.78 | 0.57 | 0.96 | 0.89 | 0.84 | 0.88 | 0.44 | 0.44 |
| **MRMR** | 94.57 | 94.93 | 94.75 | 94.57 | 93.62 | 94.57 | 94.57 | 94.57 | 94.57 | 94.57 |
|  | 1.06 | 0.95 | 1.00 | 0.91 | 0.95 | 0.98 | 0.91 | 0.92 | 0.95 | 0.95 |
| **SVM-RFE** | 96.00 | 96.00 | 95.46 | 95.52 | 95.52 | 95.52 | 94.76 | 94.61 | 94.60 | 94.64 |
|  | 0.61 | 0.68 | 0.68 | 0.76 | 0.48 | 0.49 | 0.79 | 0.76 | 0.46 | 0.65 |
| **Inf. Gain** | 93.00 | 92.64 | 91.75 | 91.57 | 91.57 | 90.62 | 91.57 | 91.53 | 91.57 | 91.44 |
|  | 0.98 | 0.96 | 0.86 | 0.81 | 0.88 | 0.81 | 0.95 | 0.52 | 0.95 | 0.90 |
| **Wilcoxon test** | 91.86 | 92.64 | 92.82 | 91.57 | 90.62 | 91.57 | 91.57 | 91.23 | 91.41 | 91.51 |
|  | 0.86 | 0.80 | 0.79 | 0.95 | 0.92 | 0.90 | 0.81 | 0.70 | 0.68 | 0.65 |
| **t-test** | 92.43 | 92.29 | 91.93 | 91.57 | 90.62 | 91.57 | 91.57 | 91.46 | 91.48 | 91.57 |
|  | 0.35 | 0.27 | 0.23 | 0.95 | 0.95 | 0.95 | 0.95 | 0.06 | 0.72 | 0.75 |
| **PCR** | 92.43 | 92.29 | 92.11 | 91.57 | 91.10 | 90.14 | 91.57 | 91.42 | 91.54 | 91.51 |
|  | 0.99 | 0.98 | 0.81 | 0.87 | 0.95 | 0.82 | 0.88 | 0.72 | 0.75 | 0.65 |
| **F-Score** | 92.14 | 92.29 | 91.93 | 91.57 | 91.10 | 91.57 | 91.57 | 91.35 | 91.54 | 91.57 |
|  | 0.99 | 1.00 | 0.88 | 0.49 | 0.48 | 0.62 | 0.70 | 0.36 | 0.75 | 0.60 |
| **Gain Ratio** | 92.14 | 91.93 | 91.75 | 91.57 | 91.10 | 91.57 | 91.57 | 91.53 | 91.48 | 91.57 |
|  | 0.99 | 0.88 | 0.79 | 0.79 | 0.76 | 0.90 | 0.62 | 0.76 | 0.72 | 0.75 |
| **BSM** | 98.54 | 98.54 | 98.54 | 97.11 | 97.11 | 96.64 | 96.73 | 96.78 | 96.92 | 96.98 |
|  | 0.49 | 0.48 | 0.42 | 0.40 | 0.38 | 0.19 | 0.17 | 0.13 | 0.11 | 0.13 |
| **SVM-PBF classifier** | | | | | | | | | | |
| **SVM-MRMR** | 95.57 | 95.75 | 95.75 | 95.57 | 94.62 | 95.57 | 95.38 | 95.53 | 95.48 | 95.57 |
|  | 0.95 | 0.92 | 0.81 | 0.52 | 0.95 | 0.81 | 0.76 | 0.76 | 0.54 | 0.55 |
| **MRMR** | 94.57 | 94.75 | 94.39 | 94.57 | 94.57 | 93.62 | 94.38 | 94.50 | 94.54 | 94.51 |
|  | 0.79 | 0.71 | 0.98 | 0.95 | 0.92 | 0.91 | 0.90 | 0.75 | 0.75 | 0.65 |
| **SVM-RFE** | 96.71 | 97.00 | 96.64 | 97.00 | 97.00 | 97.00 | 96.33 | 95.87 | 95.73 | 95.64 |
|  | 0.86 | 0.86 | 0.34 | 0.96 | 0.82 | 0.57 | 0.76 | 0.96 | 0.68 | 0.65 |
| **Inf. Gain** | 93.00 | 92.46 | 91.57 | 91.57 | 91.57 | 91.57 | 91.57 | 91.35 | 91.41 | 91.18 |
|  | 0.81 | 0.85 | 0.87 | 0.78 | 0.86 | 0.79 | 0.64 | 0.78 | 0.68 | 0.66 |
| **Wilcoxon test** | 91.86 | 92.64 | 92.82 | 91.57 | 91.57 | 91.57 | 91.57 | 91.16 | 91.29 | 91.51 |
|  | 0.86 | 0.80 | 0.79 | 0.77 | 0.77 | 0.79 | 0.79 | 0.65 | 0.97 | 0.65 |
| **t-test** | 91.86 | 91.57 | 91.75 | 91.57 | 91.57 | 91.57 | 91.57 | 91.38 | 91.51 | 91.57 |
|  | 0.86 | 0.89 | 0.79 | 0.79 | 0.68 | 0.68 | 0.43 | 0.79 | 0.83 | 0.83 |
| **PCR** | 91.86 | 91.57 | 91.57 | 90.62 | 90.62 | 91.57 | 91.29 | 91.35 | 91.41 | 91.51 |
|  | 0.86 | 0.89 | 0.71 | 0.95 | 0.98 | 0.91 | 0.85 | 0.86 | 0.77 | 0.65 |
| **F-Score** | 91.86 | 91.57 | 91.57 | 91.57 | 91.57 | 91.57 | 91.57 | 91.27 | 91.35 | 91.44 |
|  | 0.86 | 0.89 | 0.81 | 0.71 | 0.67 | 0.57 | 0.59 | 0.96 | 0.74 | 0.59 |
| **Gain Ratio** | 91.86 | 91.93 | 91.75 | 91.57 | 91.57 | 91.57 | 91.48 | 91.46 | 91.41 | 91.38 |
|  | 0.86 | 0.34 | 0.79 | 0.57 | 0.79 | 0.77 | 0.95 | 0.82 | 0.82 | 0.70 |
| **BSM** | 98.17 | 98.17 | 98.17 | 96.74 | 96.74 | 96.74 | 96.36 | 96.55 | 96.67 | 96.74 |
|  | 0.17 | 0.12 | 0.14 | 0.16 | 0.17 | 0.17 | 0.18 | 0.08 | 0.04 | 0.03 |
| **SVM-RBF classifier** | | | | | | | | | | |
| **SVM-MRMR** | 95.57 | 95.93 | 95.75 | 95.57 | 95.57 | 95.57 | 95.48 | 95.57 | 95.57 | 95.57 |
|  | 0.80 | 0.85 | 0.79 | 0.77 | 0.76 | 0.78 | 0.95 | 0.98 | 0.96 | 0.86 |
| **MRMR** | 94.57 | 94.93 | 94.75 | 94.57 | 94.57 | 94.57 | 94.57 | 94.57 | 94.54 | 94.57 |
|  | 0.79 | 0.79 | 0.77 | 0.57 | 0.86 | 0.71 | 0.79 | 0.79 | 0.75 | 0.75 |
| **SVM-RFE** | 96.57 | 95.86 | 96.93 | 96.57 | 96.57 | 96.57 | 96.57 | 96.57 | 96.57 | 96.57 |
|  | 0.82 | 0.88 | 0.71 | 0.77 | 0.67 | 0.66 | 0.66 | 0.76 | 0.57 | 0.71 |
| **Inf. Gain** | 91.57 | 91.57 | 91.39 | 91.57 | 91.57 | 91.57 | 91.57 | 91.42 | 91.57 | 91.57 |
|  | 0.86 | 0.79 | 0.71 | 0.57 | 0.57 | 0.43 | 0.88 | 0.79 | 0.91 | 0.82 |
| **Wilcoxon test** | 91.86 | 92.64 | 92.82 | 91.57 | 91.57 | 91.57 | 91.57 | 91.57 | 91.57 | 91.57 |
|  | 0.86 | 0.80 | 0.79 | 0.77 | 0.57 | 0.71 | 0.79 | 0.68 | 0.86 | 0.86 |
| **t-test** | 92.14 | 91.93 | 91.75 | 91.57 | 91.57 | 91.57 | 91.57 | 91.53 | 91.54 | 91.57 |
|  | 0.99 | 0.80 | 0.79 | 0.79 | 0.74 | 0.88 | 0.79 | 0.84 | 0.73 | 0.67 |
| **PCR** | 92.14 | 92.11 | 91.57 | 91.57 | 91.57 | 90.62 | 91.57 | 91.57 | 91.57 | 91.57 |
|  | 0.35 | 0.26 | 0.26 | 0.26 | 0.26 | 0.48 | 0.48 | 0.48 | 0.48 | 0.48 |
| **F-Score** | 92.14 | 92.11 | 91.57 | 91.57 | 91.57 | 91.57 | 91.38 | 91.57 | 91.57 | 91.51 |
|  | 0.35 | 0.26 | 0.26 | 0.26 | 0.26 | 0.26 | 0.13 | 0.13 | 0.13 | 0.65 |
| **Gain Ratio** | 91.86 | 91.93 | 91.39 | 91.57 | 91.57 | 91.57 | 91.57 | 91.57 | 91.48 | 91.57 |
|  | 0.86 | 0.80 | 0.79 | 0.86 | 0.71 | 0.86 | 0.86 | 0.79 | 0.95 | 0.90 |
| **BSM** | 98.77 | 98.77 | 98.59 | 97.34 | 97.34 | 97.34 | 97.34 | 97.34 | 97.34 | 97.34 |
|  | 0.18 | 0.16 | 0.14 | 0.11 | 0.13 | 0.17 | 0.11 | 0.16 | 0.18 | 0.11 |
| **SVM-SBF classifier** | | | | | | | | | | |
| **SVM-MRMR** | 34.00 | 34.82 | 34.82 | 36.67 | 32.86 | 34.76 | 34.86 | 34.77 | 34.86 | 34.61 |
|  | 1.46 | 1.26 | 0.85 | 2.65 | 1.65 | 0.48 | 0.99 | 0.62 | 0.56 | 0.56 |
| **MRMR** | 36.00 | 35.71 | 36.43 | 34.76 | 35.71 | 35.24 | 34.57 | 35.68 | 34.76 | 34.94 |
|  | 1.53 | 1.24 | 0.54 | 3.33 | 0.82 | 1.26 | 0.80 | 0.45 | 0.50 | 0.65 |
| **SVM-RFE** | 34.29 | 35.36 | 35.18 | 32.86 | 38.10 | 33.81 | 35.71 | 34.74 | 34.92 | 35.26 |
|  | 1.92 | 1.07 | 0.85 | 2.18 | 0.95 | 2.08 | 0.75 | 0.55 | 0.46 | 0.64 |
| **Inf. Gain** | 36.86 | 34.11 | 35.89 | 32.86 | 33.81 | 37.62 | 34.10 | 33.87 | 35.90 | 35.84 |
|  | 1.31 | 1.39 | 0.91 | 1.43 | 1.72 | 2.38 | 0.75 | 0.56 | 0.55 | 0.61 |
| **Wilcoxon test** | 34.00 | 36.07 | 36.07 | 37.14 | 35.24 | 34.76 | 34.95 | 35.38 | 36.16 | 34.87 |
|  | 1.94 | 0.59 | 1.07 | 0.82 | 0.48 | 1.26 | 0.98 | 0.60 | 0.46 | 0.70 |
| **t-test** | 35.14 | 32.14 | 36.79 | 32.86 | 36.67 | 32.86 | 35.33 | 34.96 | 35.02 | 34.81 |
|  | 1.16 | 1.66 | 0.84 | 0.82 | 1.72 | 2.18 | 0.94 | 0.53 | 0.50 | 0.68 |
| **PCR** | 35.43 | 34.64 | 35.18 | 37.14 | 34.76 | 33.33 | 34.57 | 35.41 | 35.71 | 35.39 |
|  | 0.95 | 1.00 | 0.60 | 2.86 | 1.26 | 0.48 | 0.89 | 0.67 | 0.36 | 0.71 |
| **F-Score** | 34.86 | 31.43 | 35.36 | 31.90 | 38.10 | 33.33 | 33.90 | 35.23 | 36.22 | 36.88 |
|  | 2.00 | 1.08 | 1.14 | 2.38 | 1.72 | 0.48 | 0.95 | 0.49 | 0.45 | 0.68 |
| **Gain Ratio** | 34.00 | 34.82 | 35.00 | 31.90 | 36.19 | 35.24 | 34.29 | 35.23 | 35.62 | 35.52 |
|  | 0.83 | 1.14 | 1.24 | 1.72 | 3.12 | 1.90 | 0.95 | 0.57 | 0.48 | 1.00 |
| **BSM** | 39.86 | 41.79 | 40.89 | 42.62 | 38.33 | 41.67 | 39.67 | 40.00 | 40.75 | 40.39 |
|  | 0.97 | 0.89 | 1.13 | 1.26 | 0.95 | 1.26 | 0.84 | 0.46 | 0.47 | 0.80 |

For each method, the first row represents the value of mean CA and second row represents the value of standard error in CA

**Table S14.** Mean and standard error in classification accuracy computed through varying sliding windows technique for bacterial stress in rice.

|  | **Gene sets** | | | | | | | | | |
| --- | --- | --- | --- | --- | --- | --- | --- | --- | --- | --- |
| **Methods** | **10** | **20** | **50** | **100** | **150** | **200** | **500** | **1000** | **1200** | **1500** |
| **SVM-LBF classifier** | | | | | | | | | | |
| **SVM-MRMR** | 96.54 | 96.54 | 96.54 | 96.54 | 96.54 | 96.54 | 96.45 | 96.44 | 96.00 | 95.74 |
|  | 1.90 | 1.60 | 1.13 | 1.19 | 1.09 | 1.11 | 0.90 | 0.60 | 0.13 | 0.19 |
| **MRMR** | 95.77 | 95.77 | 95.77 | 95.77 | 95.77 | 95.77 | 95.68 | 95.55 | 95.47 | 94.91 |
|  | 2.19 | 2.08 | 2.11 | 1.72 | 1.21 | 1.09 | 0.89 | 0.81 | 0.11 | 0.21 |
| **SVM-RFE** | 96.33 | 96.33 | 96.54 | 96.54 | 96.54 | 95.64 | 96.54 | 96.54 | 96.51 | 96.24 |
|  | 1.89 | 1.46 | 1.30 | 1.03 | 1.00 | 0.90 | 0.57 | 0.46 | 0.03 | 0.20 |
| **Inf. Gain** | 92.77 | 92.77 | 92.77 | 92.77 | 92.77 | 92.32 | 91.96 | 92.05 | 91.90 | 90.74 |
|  | 2.45 | 2.26 | 2.19 | 1.50 | 1.45 | 0.45 | 0.26 | 0.19 | 0.17 | 0.37 |
| **Wilcoxon test** | 92.88 | 92.88 | 92.88 | 92.88 | 92.88 | 92.88 | 92.61 | 92.77 | 92.55 | 92.26 |
|  | 2.14 | 1.99 | 1.75 | 1.32 | 1.14 | 0.99 | 0.14 | 0.06 | 0.10 | 0.32 |
| **t-test** | 92.88 | 92.88 | 92.88 | 92.88 | 92.88 | 92.88 | 92.61 | 92.31 | 92.25 | 91.03 |
|  | 2.44 | 1.97 | 1.88 | 1.72 | 1.66 | 0.86 | 0.14 | 0.17 | 0.18 | 0.42 |
| **PCR** | 92.77 | 92.77 | 92.77 | 92.77 | 92.77 | 92.32 | 92.59 | 92.45 | 92.25 | 91.66 |
|  | 2.66 | 1.54 | 1.16 | 1.20 | 1.36 | 0.77 | 0.12 | 0.11 | 0.14 | 0.35 |
| **F-Score** | 92.88 | 92.88 | 92.88 | 92.88 | 92.88 | 92.88 | 92.88 | 92.31 | 92.01 | 90.97 |
|  | 2.57 | 1.39 | 1.13 | 1.09 | 1.03 | 0.99 | 0.85 | 0.15 | 0.17 | 0.34 |
| **Gain Ratio** | 92.17 | 92.17 | 92.17 | 92.17 | 92.17 | 92.17 | 92.08 | 91.99 | 91.38 | 89.59 |
|  | 2.12 | 2.59 | 1.59 | 1.32 | 1.12 | 1.07 | 0.90 | 0.22 | 0.25 | 0.44 |
| **BSM** | 97.88 | 97.88 | 96.88 | 96.88 | 96.88 | 96.43 | 96.61 | 96.52 | 96.13 | 95.53 |
|  | 1.11 | 1.85 | 1.18 | 1.11 | 0.67 | 0.45 | 0.20 | 0.11 | 0.11 | 0.27 |
| **SVM-PBF classifier** | | | | | | | | | | |
| **SVM-MRMR** | 96.11 | 96.11 | 96.11 | 96.11 | 96.11 | 96.11 | 96.02 | 95.93 | 95.75 | 94.75 |
|  | 1.90 | 1.75 | 1.12 | 1.29 | 1.02 | 1.01 | 0.09 | 0.08 | 0.12 | 0.29 |
| **MRMR** | 94.55 | 94.55 | 94.55 | 94.55 | 94.10 | 94.55 | 94.55 | 94.37 | 94.22 | 93.20 |
|  | 2.45 | 2.05 | 2.11 | 1.75 | 1.22 | 0.98 | 0.45 | 0.08 | 0.10 | 0.28 |
| **SVM-RFE** | 96.04 | 96.04 | 96.04 | 95.59 | 96.04 | 96.04 | 96.04 | 96.00 | 96.04 | 95.42 |
|  | 1.25 | 1.05 | 1.15 | 0.95 | 0.84 | 1.04 | 0.94 | 0.74 | 0.04 | 0.21 |
| **Inf. Gain** | 91.88 | 91.88 | 91.88 | 91.88 | 91.88 | 91.55 | 91.01 | 91.29 | 90.83 | 89.67 |
|  | 2.45 | 1.95 | 1.50 | 1.45 | 0.95 | 0.75 | 0.41 | 0.17 | 0.22 | 0.49 |
| **Wilcoxon test** | 91.77 | 91.77 | 91.77 | 91.77 | 91.77 | 91.77 | 91.41 | 91.59 | 91.56 | 91.15 |
|  | 2.21 | 1.92 | 1.82 | 1.71 | 1.55 | 1.33 | 0.81 | 0.51 | 0.09 | 0.25 |
| **t-test** | 91.43 | 91.43 | 91.43 | 91.43 | 91.43 | 91.43 | 91.16 | 91.01 | 90.77 | 90.14 |
|  | 2.14 | 1.81 | 1.14 | 1.66 | 0.94 | 0.74 | 0.44 | 0.15 | 0.19 | 0.27 |
| **PCR** | 91.11 | 91.11 | 91.11 | 91.11 | 91.11 | 91.11 | 90.66 | 91.11 | 90.87 | 90.00 |
|  | 0.28 | 0.28 | 0.28 | 0.28 | 0.28 | 0.28 | 0.28 | 0.19 | 0.09 | 0.35 |
| **F-Score** | 91.43 | 91.43 | 91.43 | 91.43 | 91.43 | 91.43 | 91.43 | 90.79 | 90.62 | 88.98 |
|  | 1.73 | 1.73 | 1.73 | 1.73 | 1.73 | 1.73 | 1.73 | 0.14 | 0.18 | 0.52 |
| **Gain Ratio** | 92.10 | 92.10 | 92.10 | 92.10 | 92.10 | 92.10 | 92.10 | 91.75 | 91.41 | 88.73 |
|  | 1.76 | 1.76 | 1.76 | 1.76 | 1.76 | 1.76 | 1.76 | 0.13 | 0.14 | 0.56 |
| **BSM** | 97.65 | 97.65 | 97.65 | 97.65 | 97.20 | 96.75 | 97.56 | 97.26 | 96.81 | 96.24 |
|  | 1.42 | 1.12 | 1.12 | 1.12 | 0.45 | 0.45 | 0.09 | 0.10 | 0.14 | 0.27 |
| **SVM-RBF classifier** | | | | | | | | | | |
| **SVM-MRMR** | 96.54 | 96.54 | 96.54 | 96.54 | 96.54 | 96.09 | 95.55 | 94.69 | 93.63 | 90.03 |
|  | 1.65 | 1.60 | 0.96 | 0.90 | 0.84 | 0.45 | 0.36 | 0.24 | 0.23 | 0.35 |
| **MRMR** | 95.77 | 95.77 | 95.77 | 95.77 | 95.77 | 95.77 | 94.50 | 94.09 | 93.00 | 89.19 |
|  | 1.52 | 0.86 | 0.65 | 0.52 | 0.86 | 0.71 | 0.52 | 0.20 | 0.30 | 0.39 |
| **SVM-RFE** | 96.38 | 95.47 | 95.13 | 93.95 | 93.50 | 92.60 | 94.85 | 94.66 | 94.76 | 94.01 |
|  | 1.27 | 0.70 | 0.83 | 2.06 | 0.90 | 0.80 | 0.22 | 0.22 | 0.16 | 0.29 |
| **Inf. Gain** | 92.55 | 92.38 | 92.55 | 92.10 | 91.20 | 88.94 | 89.12 | 87.57 | 85.82 | 81.98 |
|  | 2.17 | 1.89 | 1.69 | 1.45 | 0.75 | 1.62 | 0.56 | 0.48 | 0.39 | 0.71 |
| **Wilcoxon test** | 92.33 | 92.33 | 92.33 | 91.88 | 90.97 | 90.52 | 90.16 | 90.41 | 89.62 | 87.23 |
|  | 2.45 | 1.75 | 1.50 | 1.11 | 0.95 | 0.85 | 0.52 | 0.30 | 0.31 | 0.49 |
| **t-test** | 92.55 | 92.55 | 92.55 | 92.55 | 91.20 | 90.75 | 89.57 | 89.24 | 87.89 | 83.58 |
|  | 1.59 | 1.93 | 1.72 | 1.55 | 1.12 | 0.45 | 0.59 | 0.37 | 0.46 | 0.75 |
| **PCR** | 92.46 | 92.46 | 92.46 | 92.01 | 91.11 | 91.11 | 90.84 | 90.15 | 89.84 | 87.24 |
|  | 1.78 | 1.20 | 0.88 | 0.45 | 0.78 | 0.78 | 0.38 | 0.39 | 0.28 | 0.48 |
| **F-Score** | 93.87 | 93.87 | 93.87 | 93.87 | 92.96 | 90.26 | 91.25 | 88.42 | 86.63 | 83.05 |
|  | 1.24 | 1.37 | 1.29 | 1.12 | 0.90 | 0.90 | 0.28 | 0.44 | 0.55 | 0.62 |
| **Gain Ratio** | 93.34 | 93.88 | 93.88 | 91.17 | 92.53 | 91.17 | 88.92 | 87.26 | 85.65 | 81.78 |
|  | 1.96 | 1.60 | 1.35 | 1.94 | 1.35 | 0.78 | 0.41 | 0.41 | 0.33 | 0.64 |
| **BSM** | 97.16 | 97.26 | 97.26 | 95.18 | 94.73 | 94.95 | 94.77 | 94.26 | 93.54 | 90.79 |
|  | 0.27 | 0.17 | 0.17 | 0.90 | 0.78 | 0.78 | 0.39 | 0.23 | 0.27 | 0.66 |
| **SVM-SBF classifier** | | | | | | | | | | |
| **SVM-MRMR** | 36.18 | 36.99 | 35.13 | 36.54 | 31.13 | 37.44 | 36.18 | 36.95 | 36.72 | 37.91 |
|  | 1.18 | 1.25 | 1.69 | 2.51 | 1.19 | 0.45 | 0.68 | 0.56 | 0.60 | 0.76 |
| **MRMR** | 34.59 | 34.80 | 34.29 | 35.14 | 36.94 | 36.49 | 36.85 | 36.02 | 36.01 | 36.00 |
|  | 1.01 | 0.91 | 0.99 | 2.81 | 0.45 | 1.35 | 0.83 | 0.48 | 0.48 | 0.91 |
| **SVM-RFE** | 35.95 | 34.63 | 36.99 | 35.14 | 39.64 | 35.59 | 35.05 | 36.88 | 35.74 | 36.86 |
|  | 0.92 | 0.67 | 0.88 | 1.35 | 0.45 | 2.51 | 1.15 | 0.48 | 0.62 | 0.78 |
| **Inf. Gain** | 33.78 | 34.80 | 37.84 | 34.23 | 36.94 | 36.04 | 35.86 | 34.89 | 37.33 | 35.75 |
|  | 1.42 | 1.84 | 1.05 | 2.74 | 1.19 | 1.96 | 0.81 | 0.55 | 0.41 | 0.85 |
| **Wilcoxon test** | 33.78 | 35.14 | 36.99 | 36.49 | 38.29 | 41.44 | 36.31 | 34.99 | 36.58 | 35.81 |
|  | 0.60 | 1.25 | 1.02 | 1.56 | 2.38 | 0.45 | 0.76 | 0.59 | 0.47 | 0.77 |
| **t-test** | 36.76 | 36.66 | 35.30 | 36.94 | 37.39 | 36.49 | 36.04 | 35.95 | 35.65 | 36.98 |
|  | 1.16 | 0.74 | 1.04 | 0.45 | 1.96 | 1.35 | 0.87 | 0.51 | 0.44 | 0.66 |
| **PCR** | 35.68 | 33.78 | 35.81 | 35.59 | 33.78 | 36.94 | 35.05 | 35.35 | 36.13 | 37.29 |
|  | 1.58 | 1.40 | 1.53 | 1.62 | 0.78 | 3.52 | 0.90 | 0.50 | 0.47 | 0.63 |
| **F-Score** | 37.03 | 34.80 | 35.47 | 39.64 | 34.23 | 36.04 | 36.13 | 35.95 | 35.68 | 35.63 |
|  | 1.46 | 1.19 | 1.71 | 0.45 | 0.45 | 1.62 | 1.02 | 0.71 | 0.46 | 0.77 |
| **Gain Ratio** | 35.41 | 35.98 | 36.49 | 36.49 | 34.23 | 34.23 | 35.14 | 36.06 | 35.59 | 37.04 |
|  | 1.16 | 1.08 | 0.88 | 1.35 | 1.62 | 1.96 | 0.96 | 0.55 | 0.55 | 0.77 |
| **BSM** | 38.76 | 37.64 | 40.85 | 35.33 | 37.14 | 36.68 | 37.95 | 37.95 | 38.07 | 40.21 |
|  | 0.70 | 1.15 | 0.75 | 1.76 | 1.15 | 2.54 | 0.78 | 0.40 | 0.35 | 0.51 |

For each method, the first row represents the value of mean CA and second row represents the value of standard error in CA

**Table S15.** Mean and standard error in classification accuracy computed through varying sliding windows technique for fungal stress in rice.

|  | **Gene sets** | | | | | | | | | |
| --- | --- | --- | --- | --- | --- | --- | --- | --- | --- | --- |
| **Methods** | **10** | **20** | **50** | **100** | **150** | **200** | **500** | **1000** | **1200** | **1500** |
| **SVM-LBF classifier** | | | | | | | | | | |
| **SVM-MRMR** | 96.21 | 96.21 | 96.21 | 96.21 | 96.21 | 96.21 | 96.21 | 95.70 | 95.87 | 95.69 |
|  | 1.26 | 0.83 | 0.83 | 0.83 | 0.83 | 0.83 | 0.83 | 0.26 | 0.21 | 0.38 |
| **MRMR** | 95.21 | 95.21 | 95.21 | 95.21 | 95.21 | 95.21 | 95.21 | 94.70 | 94.53 | 94.69 |
|  | 1.45 | 1.45 | 1.45 | 1.45 | 1.45 | 1.45 | 1.45 | 0.33 | 0.28 | 0.38 |
| **SVM-RFE** | 96.01 | 96.01 | 96.01 | 96.01 | 96.01 | 96.01 | 96.01 | 96.01 | 96.01 | 95.01 |
|  | 1.28 | 1.28 | 1.28 | 1.28 | 1.28 | 1.28 | 1.28 | 1.28 | 1.28 | 0.83 |
| **Inf. Gain** | 92.43 | 92.43 | 92.43 | 92.43 | 92.43 | 92.43 | 92.18 | 91.22 | 90.89 | 89.98 |
|  | 1.64 | 1.64 | 1.64 | 1.64 | 1.64 | 1.64 | 0.26 | 0.29 | 0.31 | 0.65 |
| **Wilcoxon test** | 92.65 | 92.65 | 92.65 | 92.65 | 92.65 | 92.65 | 92.65 | 92.25 | 91.71 | 91.96 |
|  | 1.85 | 1.85 | 1.85 | 1.85 | 1.85 | 1.85 | 1.85 | 0.24 | 0.33 | 0.54 |
| **t-test** | 93.10 | 93.10 | 93.10 | 93.10 | 93.10 | 93.10 | 92.59 | 91.28 | 91.31 | 90.13 |
|  | 1.94 | 1.94 | 1.94 | 1.94 | 1.94 | 1.94 | 0.35 | 0.40 | 0.31 | 0.67 |
| **PCR** | 93.44 | 93.44 | 93.44 | 93.44 | 93.44 | 93.44 | 92.67 | 92.83 | 93.18 | 93.09 |
|  | 1.71 | 1.71 | 1.71 | 1.71 | 1.71 | 1.71 | 0.41 | 0.27 | 0.14 | 0.24 |
| **F-Score** | 93.63 | 93.63 | 93.63 | 93.63 | 93.63 | 93.63 | 93.63 | 93.23 | 93.46 | 93.28 |
|  | 1.94 | 1.94 | 1.94 | 1.94 | 1.94 | 1.94 | 1.94 | 1.19 | 0.92 | 0.35 |
| **Gain Ratio** | 93.43 | 93.43 | 93.43 | 93.43 | 93.43 | 93.43 | 93.18 | 93.13 | 93.26 | 93.43 |
|  | 1.83 | 1.83 | 1.83 | 1.83 | 1.83 | 1.83 | 1.26 | 0.22 | 0.17 | 0.21 |
| **BSM** | 97.14 | 97.14 | 97.14 | 97.14 | 97.14 | 97.14 | 97.14 | 97.14 | 97.14 | 97.14 |
|  | 0.83 | 0.83 | 0.83 | 0.83 | 0.83 | 0.83 | 0.83 | 0.83 | 0.83 | 0.83 |
| **SVM-PBF classifier** | | | | | | | | | | |
| **SVM-MRMR** | 96.04 | 96.04 | 95.56 | 96.04 | 96.04 | 96.04 | 95.79 | 94.73 | 94.50 | 94.64 |
|  | 1.48 | 1.48 | 1.48 | 0.86 | 0.86 | 0.86 | 0.26 | 0.33 | 0.28 | 0.40 |
| **MRMR** | 94.65 | 94.65 | 94.17 | 94.65 | 94.65 | 94.65 | 94.14 | 93.14 | 92.86 | 92.38 |
|  | 1.81 | 1.81 | 1.48 | 0.81 | 0.81 | 0.81 | 0.35 | 0.34 | 0.29 | 0.70 |
| **SVM-RFE** | 96.77 | 96.77 | 94.84 | 96.77 | 96.77 | 96.77 | 96.77 | 96.77 | 96.77 | 96.24 |
|  | 1.26 | 1.26 | 1.89 | 0.89 | 0.89 | 0.89 | 0.89 | 0.89 | 0.89 | 0.38 |
| **Inf. Gain** | 92.65 | 92.65 | 92.17 | 92.65 | 92.65 | 92.65 | 90.60 | 89.52 | 88.81 | 88.28 |
|  | 1.81 | 1.81 | 0.81 | 0.77 | 0.77 | 0.77 | 0.51 | 0.32 | 0.32 | 0.46 |
| **Wilcoxon test** | 92.88 | 92.88 | 92.88 | 92.88 | 92.88 | 92.88 | 91.85 | 90.75 | 90.57 | 90.25 |
|  | 1.80 | 1.80 | 1.80 | 1.80 | 1.80 | 1.80 | 0.45 | 0.35 | 0.31 | 0.47 |
| **t-test** | 92.33 | 92.33 | 92.33 | 92.33 | 92.33 | 92.33 | 91.04 | 89.59 | 88.22 | 88.31 |
|  | 1.85 | 1.85 | 1.85 | 1.85 | 1.85 | 1.85 | 1.48 | 0.43 | 0.43 | 0.54 |
| **PCR** | 92.65 | 92.65 | 92.17 | 92.65 | 92.65 | 92.65 | 91.63 | 90.83 | 90.35 | 89.86 |
|  | 1.48 | 1.48 | 1.77 | 0.85 | 0.85 | 0.85 | 0.45 | 0.32 | 0.35 | 0.52 |
| **F-Score** | 93.28 | 93.28 | 93.28 | 93.28 | 93.28 | 93.28 | 93.28 | 92.16 | 92.34 | 92.58 |
|  | 0.99 | 0.99 | 0.99 | 0.99 | 0.99 | 0.99 | 0.99 | 0.29 | 0.28 | 0.32 |
| **Gain Ratio** | 92.65 | 92.65 | 92.65 | 92.65 | 91.37 | 91.37 | 91.12 | 91.64 | 91.54 | 92.65 |
|  | 1.82 | 1.82 | 1.82 | 1.82 | 1.28 | 1.28 | 0.50 | 0.38 | 0.31 | 0.46 |
| **BSM** | 97.14 | 96.66 | 97.14 | 97.14 | 97.14 | 97.14 | 97.14 | 96.94 | 97.14 | 96.97 |
|  | 0.81 | 0.48 | 0.48 | 0.48 | 0.48 | 0.48 | 0.48 | 0.14 | 0.11 | 0.17 |
| **SVM-RBF classifier** | | | | | | | | | | |
| **SVM-MRMR** | 96.12 | 96.12 | 96.12 | 96.12 | 96.12 | 96.12 | 96.12 | 95.51 | 95.61 | 95.77 |
|  | 1.56 | 1.56 | 1.56 | 1.56 | 1.56 | 1.56 | 1.56 | 0.23 | 0.20 | 0.24 |
| **MRMR** | 94.65 | 94.17 | 94.65 | 94.65 | 94.65 | 94.65 | 94.65 | 94.05 | 94.31 | 94.48 |
|  | 1.48 | 0.48 | 0.27 | 0.27 | 0.27 | 0.27 | 0.27 | 0.27 | 0.17 | 0.17 |
| **SVM-RFE** | 96.05 | 96.05 | 94.13 | 96.05 | 93.49 | 96.05 | 95.29 | 96.05 | 96.05 | 96.05 |
|  | 1.26 | 1.26 | 1.59 | 2.56 | 2.41 | 2.56 | 0.77 | 0.77 | 0.77 | 0.77 |
| **Inf. Gain** | 92.65 | 91.69 | 92.65 | 92.65 | 92.65 | 92.65 | 92.14 | 90.43 | 89.83 | 89.33 |
|  | 1.96 | 0.96 | 0.35 | 0.35 | 0.35 | 0.35 | 0.49 | 0.40 | 0.39 | 0.29 |
| **Wilcoxon test** | 92.43 | 92.43 | 92.43 | 92.43 | 92.43 | 92.43 | 92.43 | 92.23 | 92.09 | 92.26 |
|  | 1.91 | 1.91 | 1.91 | 1.91 | 1.91 | 1.91 | 1.91 | 1.14 | 0.17 | 0.17 |
| **t-test** | 92.65 | 92.65 | 92.65 | 92.65 | 92.65 | 92.65 | 91.89 | 90.53 | 90.69 | 90.03 |
|  | 1.41 | 1.41 | 1.41 | 1.41 | 1.41 | 1.41 | 0.81 | 0.40 | 0.29 | 0.47 |
| **PCR** | 92.43 | 92.43 | 92.43 | 92.43 | 92.43 | 92.43 | 92.18 | 92.03 | 92.43 | 91.73 |
|  | 1.64 | 1.64 | 1.64 | 1.64 | 1.64 | 1.64 | 0.83 | 0.19 | 0.94 | 0.32 |
| **F-Score** | 93.54 | 93.54 | 93.54 | 93.54 | 93.54 | 92.26 | 93.54 | 92.43 | 92.86 | 93.54 |
|  | 1.82 | 1.82 | 1.82 | 1.82 | 1.82 | 1.28 | 1.82 | 0.35 | 0.25 | 0.00 |
| **Gain Ratio** | 93.43 | 92.47 | 93.43 | 90.87 | 93.43 | 93.43 | 92.66 | 93.13 | 93.35 | 93.26 |
|  | 1.96 | 0.96 | 2.56 | 1.56 | 0.73 | 0.73 | 0.41 | 0.22 | 0.09 | 0.17 |
| **BSM** | 97.02 | 96.54 | 95.09 | 97.02 | 97.02 | 97.02 | 97.02 | 97.02 | 97.02 | 97.02 |
|  | 0.48 | 0.48 | 1.26 | 0.26 | 0.26 | 0.26 | 0.26 | 0.26 | 0.06 | 0.26 |
| **SVM-SBF classifier** | | | | | | | | | | |
| **SVM-MRMR** | 30.90 | 25.96 | 27.40 | 23.08 | 24.36 | 25.64 | 28.46 | 25.10 | 26.84 | 25.35 |
|  | 3.08 | 2.27 | 1.69 | 2.22 | 3.39 | 1.28 | 1.44 | 1.04 | 0.89 | 1.18 |
| **MRMR** | 30.77 | 24.52 | 27.88 | 28.21 | 26.92 | 23.08 | 25.64 | 26.01 | 25.21 | 28.15 |
|  | 2.72 | 1.91 | 1.89 | 1.28 | 4.44 | 5.88 | 1.66 | 1.02 | 0.89 | 1.02 |
| **SVM-RFE** | 26.92 | 26.44 | 23.56 | 28.21 | 23.08 | 24.36 | 25.90 | 24.80 | 25.73 | 25.52 |
|  | 2.72 | 2.35 | 1.53 | 1.28 | 5.88 | 3.39 | 1.43 | 0.88 | 0.95 | 1.17 |
| **Inf. Gain** | 30.77 | 25.48 | 25.00 | 33.33 | 25.64 | 26.92 | 26.67 | 24.19 | 26.07 | 26.75 |
|  | 1.22 | 2.17 | 1.63 | 1.28 | 1.28 | 0.00 | 0.95 | 1.09 | 0.69 | 0.93 |
| **Wilcoxon test** | 26.92 | 27.40 | 26.44 | 24.36 | 26.92 | 28.21 | 23.33 | 26.21 | 26.84 | 26.40 |
|  | 1.72 | 0.87 | 2.56 | 2.56 | 2.22 | 4.62 | 1.48 | 0.88 | 0.80 | 0.99 |
| **t-test** | 23.08 | 26.44 | 24.52 | 26.92 | 26.92 | 23.08 | 25.13 | 25.30 | 26.67 | 26.40 |
|  | 2.43 | 1.35 | 2.99 | 2.22 | 2.22 | 4.44 | 1.54 | 0.73 | 0.77 | 1.08 |
| **PCR** | 26.15 | 26.44 | 24.04 | 21.79 | 26.92 | 29.49 | 24.87 | 26.42 | 26.58 | 25.70 |
|  | 2.24 | 2.23 | 2.02 | 1.28 | 3.85 | 5.13 | 1.24 | 1.01 | 0.78 | 1.20 |
| **F-Score** | 28.46 | 28.85 | 26.44 | 29.49 | 26.92 | 21.79 | 25.13 | 26.92 | 25.47 | 27.97 |
|  | 3.96 | 2.18 | 2.11 | 3.39 | 0.12 | 3.39 | 1.50 | 0.98 | 0.80 | 1.11 |
| **Gain Ratio** | 23.85 | 27.88 | 25.00 | 21.79 | 24.36 | 23.08 | 26.67 | 26.21 | 26.41 | 24.30 |
|  | 2.83 | 2.70 | 1.26 | 5.59 | 6.78 | 2.22 | 1.38 | 0.81 | 0.80 | 1.55 |
| **BSM** | 30.92 | 27.08 | 28.04 | 30.92 | 24.51 | 29.64 | 30.41 | 29.51 | 29.56 | 27.10 |
|  | 2.20 | 1.78 | 2.02 | 1.22 | 1.56 | 1.28 | 1.59 | 0.72 | 0.92 | 0.69 |

For each method, the first row represents the value of mean CA and second row represents the value of standard error in CA

**Table S16.** Mean and standard error in classification accuracy computed through varying sliding windows technique for insect stress in rice.

|  | **Gene sets** | | | | | | | | | |
| --- | --- | --- | --- | --- | --- | --- | --- | --- | --- | --- |
| **Methods** | **10** | **20** | **50** | **100** | **150** | **200** | **500** | **1000** | **1200** | **1500** |
| **SVM-LBF classifier** | | | | | | | | | | |
| **SVM-MRMR** | 96.54 | 96.54 | 96.54 | 96.54 | 96.54 | 95.62 | 96.54 | 96.54 | 96.48 | 96.54 |
|  | 1.26 | 1.26 | 1.26 | 1.26 | 1.26 | 1.59 | 1.17 | 1.06 | 0.62 | 0.73 |
| **MRMR** | 94.77 | 94.77 | 94.42 | 94.77 | 94.77 | 94.77 | 94.77 | 94.77 | 94.77 | 94.77 |
|  | 1.17 | 1.17 | 1.22 | 1.35 | 1.35 | 1.35 | 1.35 | 1.35 | 1.35 | 1.35 |
| **SVM-RFE** | 95.21 | 95.70 | 96.04 | 97.43 | 97.43 | 97.43 | 97.43 | 97.43 | 97.43 | 97.43 |
|  | 0.56 | 1.04 | 1.52 | 0.95 | 0.95 | 0.95 | 0.95 | 0.95 | 0.95 | 0.95 |
| **Inf. Gain** | 92.65 | 92.65 | 92.65 | 91.73 | 89.88 | 89.88 | 92.28 | 92.22 | 92.10 | 92.15 |
|  | 1.59 | 1.59 | 1.59 | 0.93 | 0.89 | 0.89 | 0.25 | 0.17 | 0.17 | 0.23 |
| **Wilcoxon test** | 92.77 | 92.77 | 92.77 | 91.84 | 92.77 | 92.77 | 92.58 | 91.45 | 91.90 | 91.88 |
|  | 1.93 | 1.93 | 1.93 | 1.25 | 1.93 | 1.93 | 0.19 | 0.34 | 0.19 | 0.28 |
| **t-test** | 92.65 | 92.65 | 92.65 | 92.65 | 92.65 | 92.65 | 92.47 | 92.14 | 92.16 | 92.28 |
|  | 1.85 | 1.85 | 1.85 | 1.85 | 1.85 | 1.85 | 0.85 | 0.77 | 0.60 | 0.80 |
| **PCR** | 92.34 | 92.34 | 92.34 | 90.49 | 92.34 | 92.34 | 92.34 | 91.03 | 91.54 | 91.08 |
|  | 1.93 | 1.93 | 1.93 | 1.26 | 0.93 | 0.93 | 0.93 | 0.31 | 0.19 | 0.30 |
| **F-Score** | 93.65 | 93.65 | 93.65 | 93.65 | 93.65 | 93.65 | 93.28 | 93.22 | 93.53 | 93.28 |
|  | 1.65 | 1.65 | 1.65 | 1.65 | 1.65 | 1.65 | 0.52 | 0.46 | 0.19 | 0.80 |
| **Gain Ratio** | 93.42 | 93.42 | 93.42 | 93.42 | 93.42 | 93.42 | 92.86 | 93.34 | 93.29 | 93.16 |
|  | 0.30 | 0.30 | 0.30 | 0.30 | 0.30 | 0.30 | 0.30 | 0.07 | 0.09 | 0.17 |
| **BSM** | 96.84 | 96.84 | 96.50 | 96.84 | 96.84 | 96.84 | 96.84 | 96.84 | 96.84 | 96.59 |
|  | 0.72 | 0.72 | 0.35 | 0.72 | 0.72 | 0.72 | 0.72 | 0.72 | 0.72 | 0.17 |
| **SVM-PBF classifier** | | | | | | | | | | |
| **SVM-MRMR** | 95.99 | 96.54 | 96.54 | 96.54 | 95.62 | 95.62 | 96.54 | 96.32 | 96.48 | 96.54 |
|  | 1.56 | 1.46 | 1.46 | 1.46 | 0.93 | 0.93 | 1.46 | 0.12 | 0.06 | 0.46 |
| **MRMR** | 94.54 | 95.31 | 95.65 | 95.65 | 95.65 | 95.65 | 95.65 | 95.65 | 95.59 | 95.65 |
|  | 1.68 | 1.55 | 1.35 | 1.35 | 1.35 | 1.35 | 1.35 | 1.35 | 0.07 | 0.66 |
| **SVM-RFE** | 94.11 | 95.92 | 95.57 | 96.76 | 96.76 | 96.76 | 96.76 | 96.76 | 96.70 | 95.76 |
|  | 1.42 | 1.14 | 0.94 | 0.73 | 0.73 | 0.73 | 0.73 | 0.73 | 0.06 | 0.51 |
| **Inf. Gain** | 92.65 | 92.65 | 92.65 | 90.80 | 89.88 | 89.88 | 92.10 | 91.12 | 91.30 | 91.64 |
|  | 1.93 | 1.93 | 1.93 | 1.59 | 0.96 | 0.96 | 0.70 | 0.71 | 0.21 | 0.29 |
| **Wilcoxon test** | 92.27 | 92.48 | 92.48 | 90.97 | 92.83 | 92.83 | 92.83 | 91.80 | 91.84 | 91.94 |
|  | 1.56 | 1.35 | 1.35 | 0.93 | 1.26 | 1.26 | 1.26 | 0.82 | 0.20 | 0.28 |
| **t-test** | 92.76 | 92.76 | 91.37 | 90.91 | 91.84 | 91.84 | 91.84 | 91.37 | 91.47 | 91.75 |
|  | 1.74 | 1.74 | 1.24 | 0.93 | 0.93 | 0.93 | 0.35 | 0.26 | 0.21 | 0.29 |
| **PCR** | 92.87 | 92.87 | 92.18 | 91.02 | 92.87 | 92.87 | 92.69 | 91.85 | 91.76 | 91.86 |
|  | 1.45 | 1.45 | 1.21 | 0.93 | 0.93 | 0.93 | 0.19 | 0.22 | 0.27 | 0.29 |
| **F-Score** | 93.09 | 93.09 | 93.09 | 93.09 | 93.09 | 93.09 | 92.72 | 92.65 | 92.84 | 92.71 |
|  | 1.25 | 1.25 | 1.25 | 1.25 | 1.25 | 1.25 | 0.65 | 0.17 | 0.12 | 0.28 |
| **Gain Ratio** | 93.02 | 93.02 | 93.02 | 93.02 | 93.02 | 93.02 | 92.46 | 92.80 | 92.83 | 92.89 |
|  | 1.30 | 1.30 | 1.30 | 1.30 | 1.30 | 1.30 | 0.70 | 0.12 | 0.10 | 0.13 |
| **BSM** | 97.32 | 97.32 | 96.63 | 97.32 | 97.32 | 97.32 | 97.32 | 97.25 | 97.20 | 96.32 |
|  | 0.85 | 0.85 | 0.66 | 0.56 | 0.56 | 0.56 | 0.56 | 0.07 | 0.09 | 0.19 |
| **SVM-PBF classifier** | | | | | | | | | | |
| **SVM-MRMR** | 96.81 | 96.81 | 96.81 | 96.81 | 96.81 | 96.81 | 96.81 | 96.74 | 96.75 | 96.81 |
|  | 1.73 | 1.73 | 1.73 | 1.73 | 1.73 | 1.73 | 1.73 | 0.90 | 0.06 | 0.42 |
| **MRMR** | 95.54 | 95.54 | 95.19 | 95.54 | 95.54 | 95.54 | 95.54 | 95.47 | 95.54 | 95.54 |
|  | 1.23 | 1.23 | 1.35 | 1.23 | 1.23 | 1.23 | 1.23 | 0.73 | 0.07 | 0.47 |
| **SVM-RFE** | 95.56 | 96.36 | 96.71 | 96.21 | 96.21 | 96.21 | 96.21 | 96.21 | 96.00 | 95.21 |
|  | 1.41 | 0.99 | 0.85 | 0.52 | 0.52 | 0.52 | 0.52 | 0.52 | 0.25 | 0.66 |
| **Inf. Gain** | 92.34 | 92.34 | 91.30 | 90.49 | 89.56 | 89.56 | 91.97 | 91.39 | 91.72 | 91.71 |
|  | 2.04 | 2.04 | 1.67 | 1.26 | 0.93 | 0.93 | 0.25 | 0.22 | 0.17 | 0.25 |
| **Wilcoxon test** | 91.70 | 91.91 | 92.26 | 90.40 | 91.33 | 92.26 | 91.88 | 91.38 | 91.39 | 90.99 |
|  | 1.46 | 0.72 | 0.35 | 0.93 | 0.93 | 0.93 | 0.25 | 0.21 | 0.19 | 0.30 |
| **t-test** | 92.10 | 92.31 | 92.31 | 90.80 | 91.73 | 91.73 | 92.65 | 91.78 | 91.79 | 92.28 |
|  | 0.56 | 0.35 | 0.35 | 0.93 | 0.93 | 0.93 | 0.93 | 0.21 | 0.19 | 0.21 |
| **PCR** | 92.32 | 92.53 | 92.18 | 91.02 | 91.95 | 92.88 | 92.88 | 92.15 | 91.89 | 91.61 |
|  | 0.56 | 0.35 | 0.69 | 0.93 | 0.93 | 0.83 | 0.83 | 0.20 | 0.25 | 0.30 |
| **F-Score** | 92.88 | 92.88 | 92.88 | 92.88 | 92.88 | 92.88 | 92.32 | 92.44 | 92.57 | 92.50 |
|  | 1.23 | 1.23 | 1.23 | 0.97 | 0.97 | 0.97 | 0.30 | 0.17 | 0.13 | 0.21 |
| **Gain Ratio** | 92.65 | 92.65 | 92.65 | 92.65 | 92.65 | 92.65 | 91.91 | 92.36 | 92.28 | 92.40 |
|  | 0.98 | 0.98 | 0.98 | 0.98 | 0.98 | 0.98 | 0.82 | 0.14 | 0.14 | 0.17 |
| **BSM** | 96.76 | 97.88 | 97.18 | 97.88 | 97.88 | 97.88 | 97.88 | 97.80 | 97.88 | 97.00 |
|  | 0.68 | 0.80 | 0.62 | 0.45 | 0.45 | 0.45 | 0.45 | 0.07 | 0.31 | 0.21 |
| **SVM-SBF classifier** | | | | | | | | | | |
| **SVM-MRMR** | 31.67 | 30.56 | 32.64 | 32.41 | 34.26 | 25.00 | 30.00 | 31.43 | 29.75 | 32.07 |
|  | 2.22 | 1.15 | 1.55 | 1.85 | 4.04 | 4.24 | 1.39 | 0.69 | 0.93 | 1.06 |
| **MRMR** | 28.89 | 29.51 | 31.25 | 31.48 | 25.00 | 30.56 | 30.93 | 29.82 | 29.63 | 29.67 |
|  | 2.26 | 1.65 | 2.21 | 2.45 | 0.23 | 2.78 | 1.41 | 0.66 | 0.77 | 1.06 |
| **SVM-RFE** | 24.44 | 25.69 | 28.82 | 30.56 | 29.63 | 29.63 | 30.93 | 29.31 | 31.05 | 29.17 |
|  | 3.45 | 2.86 | 1.57 | 0.35 | 1.85 | 2.45 | 1.05 | 0.68 | 0.83 | 1.45 |
| **Inf. Gain** | 33.89 | 29.86 | 29.17 | 25.93 | 31.48 | 24.07 | 29.07 | 30.41 | 29.63 | 30.56 |
|  | 1.04 | 1.59 | 1.29 | 0.93 | 2.45 | 3.34 | 1.35 | 0.78 | 0.66 | 1.11 |
| **Wilcoxon test** | 27.78 | 30.90 | 31.25 | 29.63 | 29.63 | 35.19 | 30.93 | 30.19 | 30.06 | 31.31 |
|  | 1.76 | 1.92 | 1.87 | 1.85 | 4.63 | 3.34 | 1.84 | 0.68 | 0.75 | 0.92 |
| **t-test** | 31.11 | 30.21 | 29.51 | 33.33 | 29.63 | 32.41 | 32.78 | 29.53 | 30.99 | 30.43 |
|  | 2.39 | 1.78 | 1.48 | 1.60 | 1.85 | 2.45 | 1.16 | 0.99 | 0.72 | 1.07 |
| **PCR** | 27.22 | 30.56 | 27.78 | 25.00 | 35.19 | 26.85 | 30.37 | 29.97 | 30.25 | 31.19 |
|  | 2.69 | 2.73 | 1.57 | 1.60 | 4.04 | 1.85 | 1.50 | 0.84 | 0.75 | 1.47 |
| **F-Score** | 29.44 | 31.25 | 29.17 | 30.56 | 27.78 | 30.56 | 33.89 | 29.68 | 30.68 | 29.92 |
|  | 1.11 | 1.69 | 1.57 | 1.60 | 3.21 | 2.78 | 1.25 | 0.73 | 0.68 | 1.00 |
| **Gain Ratio** | 28.33 | 28.47 | 27.43 | 26.85 | 34.26 | 27.78 | 30.93 | 32.09 | 30.19 | 31.69 |
|  | 2.04 | 2.61 | 2.75 | 0.93 | 3.34 | 1.60 | 1.05 | 0.72 | 0.74 | 1.18 |
| **BSM** | 35.67 | 37.68 | 33.86 | 32.70 | 33.63 | 35.48 | 30.67 | 34.26 | 34.80 | 32.91 |
|  | 0.68 | 1.43 | 1.55 | 1.45 | 1.85 | 1.34 | 1.14 | 0.98 | 0.75 | 0.44 |

For each method, the first row represents the value of mean CA and second row represents the value of standard error in CA

Document S7. Comparative Performance analysis of Gene selection Methods Based on GO Based Dissimilarity Scores for Biotic Stresses in Rice.

The GO based dissimilarity scores for ten gene selection methods under Gene Ontology taxonomies Molecular Function (MF), Biological Process (BP) and Cellular Component (CC) were computed for biotic stresses, i.e. bacterial, fungal and insect, are shown in Tables S17 - S19.

**Table S17.** Comparative Performance analysis of gene selection methods based on GO_MF based dissimilarity score for biotic stresses in rice.

| **Methods** | **MRMR** | **SVM-RFE** | **SVM-MRMR** | **IG** | **GR** | **Wilcox** | **t** | **PCR** | **F** | **BSM** |
| --- | --- | --- | --- | --- | --- | --- | --- | --- | --- | --- |
| **Bacterial stress in rice** | | | | | | | | | | |
| 10 | 1.00 | 0.75 | 1.00 | 0.84 | 0.90 | 0.87 | 0.86 | 0.86 | 0.86 | **0.79** |
| 20 | 0.90 | 0.86 | 0.90 | 0.88 | 0.92 | 0.89 | 0.89 | 0.89 | 0.89 | **0.83** |
| 50 | 0.91 | 0.91 | 0.91 | 0.90 | 0.90 | 0.88 | 0.89 | 0.88 | 0.88 | **0.78** |
| 100 | 0.90 | 0.91 | 0.90 | 0.89 | 0.88 | 0.91 | 0.90 | 0.90 | 0.90 | **0.80** |
| 150 | 0.90 | 0.91 | 0.90 | 0.90 | 0.89 | 0.90 | 0.89 | 0.90 | 0.90 | **0.80** |
| 200 | 0.90 | 0.92 | 0.90 | 0.90 | 0.89 | 0.90 | 0.89 | 0.90 | 0.90 | **0.79** |
| 500 | 0.89 | 0.90 | 0.90 | 0.89 | 0.90 | 0.90 | 0.90 | 0.90 | 0.90 | **0.82** |
| **Fungal stress in rice** | | | | | | | | | | |
| 10 | 0.84 | 0.83 | 0.96 | 0.89 | 0.85 | 0.91 | 0.84 | 0.84 | 0.84 | **0.82** |
| 20 | 0.90 | 0.86 | 0.90 | 0.91 | 0.89 | 0.92 | 0.88 | 0.88 | 0.88 | **0.83** |
| 50 | 0.87 | 0.88 | 0.91 | 0.90 | 0.87 | 0.89 | 0.89 | 0.89 | 0.89 | **0.82** |
| 100 | 0.88 | 0.89 | 0.91 | 0.88 | 0.88 | 0.89 | 0.89 | 0.89 | 0.89 | **0.80** |
| 150 | 0.89 | 0.89 | 0.91 | 0.89 | 0.85 | 0.90 | 0.89 | 0.89 | 0.89 | **0.80** |
| 200 | 0.90 | 0.88 | 0.91 | 0.90 | 0.83 | 0.90 | 0.90 | 0.89 | 0.89 | **0.80** |
| 500 | 0.89 | 0.87 | 0.91 | 0.88 | 0.90 | 0.91 | 0.87 | 0.91 | 0.84 | **0.80** |
| **Insect stress in rice** | | | | | | | | | | |
| 10 | 0.91 | 0.87 | 0.91 | 0.92 | 0.77 | 0.78 | 0.78 | 0.83 | 0.83 | **0.81** |
| 20 | 0.90 | 0.93 | 0.90 | 0.92 | 0.92 | 0.78 | 0.90 | 0.87 | 0.87 | **0.81** |
| 50 | 0.89 | 0.87 | 0.89 | 0.90 | 0.90 | 0.89 | 0.88 | 0.91 | 0.91 | **0.80** |
| 100 | 0.90 | 0.91 | 0.90 | 0.90 | 0.91 | 0.89 | 0.88 | 0.91 | 0.91 | **0.81** |
| 150 | 0.90 | 0.90 | 0.90 | 0.89 | 0.92 | 0.89 | 0.88 | 0.90 | 0.90 | **0.81** |
| 200 | 0.90 | 0.91 | 0.90 | 0.90 | 0.90 | 0.90 | 0.88 | 0.90 | 0.90 | **0.81** |
| 500 | 0.91 | 0.90 | 0.89 | 0.89 | 0.88 | 0.89 | 0.89 | 0.90 | 0.90 | **0.85** |

Values marked as bolds represent dissimilarity scores obtained from proposed BSM approach

**Table S18.** Comparative Performance analysis of gene selection methods based on GO_BP based dissimilarity score for biotic stresses in rice.

|  | **MRMR** | **SVM-RFE** | **SVM-MRMR** | **IG** | **GR** | **Wilcox** | **t** | **PCR** | **F** | **BSM** |
| --- | --- | --- | --- | --- | --- | --- | --- | --- | --- | --- |
| **Bacterial stress in rice** | | | | | | | | | | |
| 10 | 0.97 | 0.72 | 0.97 | 0.87 | 0.87 | 0.86 | 0.93 | 0.93 | 0.93 | **0.76** |
| 20 | 0.85 | 0.91 | 0.85 | 0.91 | 0.89 | 0.87 | 0.87 | 0.87 | 0.87 | **0.79** |
| 50 | 0.87 | 0.91 | 0.87 | 0.91 | 0.87 | 0.86 | 0.89 | 0.88 | 0.88 | **0.78** |
| 100 | 0.88 | 0.90 | 0.88 | 0.91 | 0.85 | 0.88 | 0.87 | 0.87 | 0.87 | **0.80** |
| 150 | 0.88 | 0.89 | 0.87 | 0.91 | 0.86 | 0.87 | 0.86 | 0.86 | 0.86 | **0.80** |
| 200 | 0.87 | 0.90 | 0.87 | 0.91 | 0.86 | 0.86 | 0.87 | 0.87 | 0.87 | **0.80** |
| 500 | 0.86 | 0.88 | 0.88 | 0.88 | 0.88 | 0.87 | 0.87 | 0.87 | 0.87 | **0.81** |
| **Fungal stress in rice** | | | | | | | | | | |
| 10 | 0.86 | 0.78 | 0.88 | 0.81 | 0.79 | 0.83 | 0.86 | 0.86 | 0.86 | **0.77** |
| 20 | 0.87 | 0.85 | 0.83 | 0.87 | 0.80 | 0.84 | 0.88 | 0.88 | 0.88 | **0.77** |
| 50 | 0.87 | 0.84 | 0.85 | 0.88 | 0.84 | 0.85 | 0.86 | 0.86 | 0.86 | **0.76** |
| 100 | 0.84 | 0.83 | 0.86 | 0.84 | 0.86 | 0.87 | 0.85 | 0.85 | 0.85 | **0.77** |
| 150 | 0.84 | 0.84 | 0.86 | 0.87 | 0.85 | 0.87 | 0.85 | 0.85 | 0.85 | **0.75** |
| 200 | 0.85 | 0.84 | 0.87 | 0.87 | 0.84 | 0.88 | 0.86 | 0.85 | 0.85 | **0.75** |
| 500 | 0.82 | 0.83 | 0.88 | 0.86 | 0.88 | 0.88 | 0.85 | 0.87 | 0.83 | **0.77** |
| **Insect stress in rice** | | | | | | | | | | |
| 10 | 0.90 | 0.87 | 0.90 | 0.92 | 0.81 | 0.72 | 0.94 | 0.89 | 0.89 | **0.80** |
| 20 | 0.90 | 0.87 | 0.90 | 0.86 | 0.88 | 0.85 | 0.91 | 0.88 | 0.88 | **0.80** |
| 50 | 0.87 | 0.87 | 0.87 | 0.86 | 0.88 | 0.86 | 0.89 | 0.87 | 0.87 | **0.78** |
| 100 | 0.87 | 0.88 | 0.87 | 0.87 | 0.89 | 0.86 | 0.85 | 0.88 | 0.88 | **0.78** |
| 150 | 0.86 | 0.87 | 0.86 | 0.87 | 0.88 | 0.86 | 0.87 | 0.87 | 0.87 | **0.79** |
| 200 | 0.87 | 0.88 | 0.87 | 0.87 | 0.86 | 0.86 | 0.86 | 0.87 | 0.87 | **0.79** |
| 500 | 0.88 | 0.87 | 0.88 | 0.85 | 0.85 | 0.84 | 0.86 | 0.88 | 0.88 | **0.82** |

Values marked as bolds represent dissimilarity scores obtained from proposed BSM approach

**Table S19.** Comparative Performance analysis of gene selection methods based on GO_CC based dissimilarity score for biotic stresses in rice.

|  | **MRMR** | **SVM-RFE** | **SVM-MRMR** | **IG** | **GR** | **Wilcox** | **t** | **PCR** | **F** | **BSM** |
| --- | --- | --- | --- | --- | --- | --- | --- | --- | --- | --- |
| **Bacterial stress in rice** | | | | | | | | | | |
| 10 | 1.00 | 0.74 | 1.00 | 0.83 | 0.94 | 0.91 | 0.89 | 0.89 | 0.89 | **0.83** |
| 20 | 0.92 | 0.90 | 0.92 | 0.89 | 0.92 | 0.91 | 0.93 | 0.93 | 0.93 | **0.77** |
| 50 | 0.90 | 0.88 | 0.90 | 0.90 | 0.90 | 0.88 | 0.90 | 0.88 | 0.88 | **0.78** |
| 100 | 0.87 | 0.89 | 0.87 | 0.88 | 0.89 | 0.90 | 0.89 | 0.90 | 0.90 | **0.77** |
| 150 | 0.87 | 0.89 | 0.88 | 0.89 | 0.88 | 0.90 | 0.90 | 0.90 | 0.90 | **0.76** |
| 200 | 0.89 | 0.88 | 0.89 | 0.88 | 0.88 | 0.90 | 0.90 | 0.89 | 0.89 | **0.77** |
| 500 | 0.88 | 0.87 | 0.90 | 0.85 | 0.89 | 0.89 | 0.90 | 0.89 | 0.89 | **0.82** |
| **Fungal stress in rice** | | | | | | | | | | |
| 10 | 0.95 | 0.74 | 0.94 | 0.82 | 0.90 | 0.80 | 0.95 | 0.95 | 0.95 | **0.73** |
| 20 | 0.90 | 0.88 | 0.88 | 0.86 | 0.86 | 0.90 | 0.92 | 0.92 | 0.92 | **0.79** |
| 50 | 0.90 | 0.90 | 0.89 | 0.88 | 0.86 | 0.89 | 0.90 | 0.90 | 0.90 | **0.79** |
| 100 | 0.89 | 0.90 | 0.87 | 0.89 | 0.88 | 0.89 | 0.90 | 0.89 | 0.89 | **0.80** |
| 150 | 0.90 | 0.89 | 0.88 | 0.90 | 0.87 | 0.90 | 0.89 | 0.89 | 0.89 | **0.80** |
| 200 | 0.89 | 0.90 | 0.89 | 0.90 | 0.86 | 0.90 | 0.90 | 0.90 | 0.90 | **0.81** |
| 500 | 0.87 | 0.87 | 0.89 | 0.88 | 0.90 | 0.89 | 0.88 | 0.90 | 0.87 | **0.80** |
| **Insect stress in rice** | | | | | | | | | | |
| 10 | 0.88 | 0.90 | 0.88 | 0.93 | 0.81 | 0.64 | 0.82 | 0.82 | 0.82 | **0.81** |
| 20 | 0.89 | 0.89 | 0.89 | 0.88 | 0.84 | 0.80 | 0.85 | 0.88 | 0.88 | **0.79** |
| 50 | 0.90 | 0.87 | 0.90 | 0.88 | 0.84 | 0.86 | 0.86 | 0.89 | 0.89 | **0.80** |
| 100 | 0.90 | 0.88 | 0.90 | 0.88 | 0.87 | 0.86 | 0.84 | 0.90 | 0.90 | **0.79** |
| 150 | 0.90 | 0.88 | 0.90 | 0.88 | 0.86 | 0.86 | 0.85 | 0.89 | 0.89 | **0.80** |
| 200 | 0.90 | 0.89 | 0.90 | 0.88 | 0.85 | 0.86 | 0.85 | 0.89 | 0.89 | **0.80** |
| 500 | 0.90 | 0.89 | 0.88 | 0.87 | 0.87 | 0.84 | 0.87 | 0.89 | 0.89 | **0.83** |

Values marked as bolds represent dissimilarity scores obtained from proposed BSM approach

Document S8. Performance Evaluation of Gene Selection Methods Based on Runtime Criteria.

We used the runtime criteria to evaluate the performance of gene selection methods given in Table S9 of document S5. Here, the runtime refers to the amount of time required to get the informative gene sets of particular size through providing the gene expression data as input to the R functions of the respective methods. Here, we performed all analyses on a standard 8 GB RAM Dell PC with Intel(R) Core(TM) i3-6100U CPU @ 2.30GHz, 2301 Mhz, 2 Core(s) Processor(s) and recorded the computational time required to analyze Bacterial gene expression data of 8356 genes with 74 subjects (case: 37 and control: 37 ). Here, all the analyses are performed on R software with version 4.0.1. The result for bacterial datasets is shown in Table S20 and the results for the remaining datasets are shown in Table S21-23

**Table S20.** Runtime based analysis of gene selection methods in Bacterial stress dataset.

| **Sl. No.** | **Methods** | **Symbol** | **R package** |  | **Run time** | **Ranks** | **Rank score** |
| --- | --- | --- | --- | --- | --- | --- | --- |
| 01 | BSM | BSM | BSM |  | 25 Minutes | 8 | 0.3 |
| 02 | SVM (RFE)-MRMR | SVM-MRMR | BSM, e1071 |  | 1.30 hours | 10 | 0.1 |
| 03 | MRMR | MRMR | BSM |  | 10 Minutes | 7 | 0.4 |
| 04 | SVM with Recursive Feature Elimination | SVM-RFE | e1071 |  | 1.20 Hours | 9 | 0.2 |
| 05 | t-score | t | stats |  | 1.5 Minutes | 1 | 1 |
| 06 | F-score | F | stats |  | 2 Minutes | 2 | 0.9 |
| 07 | Pearson’s Linear Correlation | PCR | FSelector |  | 4 Minutes | 4 | 0.7 |
| 08 | Information Gain | IG | FSelector |  | 4.5 Minutes | 5 | 0.6 |
| 09 | Gain Ratio | GR | FSelector |  | 5 Minutes | 6 | 0.5 |
| 10 | Wilcoxon Statistic | Wilcox | stats |  | 2.5 Minutes | 3 | 0.8 |

The BSM method required less computational time than SVM-RFE and SVM-MRMR with much better performance in terms of detecting biologically informative genes. Moreover, it can even be used on a PC or workstation computer for analyzing large gene expression datasets.

**Table S21.** Runtime based analysis of gene selection methods in Salinity stress dataset.

| **Sl. No.** | **Methods** | **Symbol** | **R package** | **Run time** | **Ranks** | **Rank score** |
| --- | --- | --- | --- | --- | --- | --- |
| 01 | BSM | BSM | BSM | 15 Minutes | 8 | 0.3 |
| 02 | SVM (RFE)-MRMR | SVM-MRMR | BSM, e1071 | 75 Minutes | 10 | 0.1 |
| 03 | MRMR | MRMR | BSM | 10 Minutes | 7 | 0.4 |
| 04 | SVM with Recursive Feature Elimination | SVM-RFE | e1071 | 70 Minutes | 9 | 0.2 |
| 05 | t-score | t | stats | 1.5 Minutes | 1 | 1 |
| 06 | F-score | F | stats | 2 Minutes | 2 | 0.9 |
| 07 | Pearson’s Linear Correlation | PCR | FSelector | 4 Minutes | 4 | 0.7 |
| 08 | Information Gain | IG | FSelector | 4.5 Minutes | 5 | 0.6 |
| 09 | Gain Ratio | GR | FSelector | 5 Minutes | 6 | 0.5 |
| 10 | Wilcoxon Statistic | Wilcox | stats | 2.5 Minutes | 3 | 0.8 |

**Table S22.** Runtime based analysis of gene selection methods in Drought stress dataset (9078 genes over 70 samples).

| **Sl. No.** | **Methods** | **Symbol** | **Run time** | **Ranks** | **Rank score** |
| --- | --- | --- | --- | --- | --- |
| 01 | BSM | BSM | 30 Minutes | 8 | 0.3 |
| 02 | SVM (RFE)-MRMR | SVM-MRMR | 1.40 hours | 10 | 0.1 |
| 03 | MRMR | MRMR | 15 Minutes | 7 | 0.4 |
| 04 | SVM with Recursive Feature Elimination | SVM-RFE | 1.30 Hours | 9 | 0.2 |
| 05 | t-score | t | 2.5 Minutes | 1 | 1 |
| 06 | F-score | F | 3 Minutes | 2 | 0.9 |
| 07 | Pearson’s Linear Correlation | PCR | 5 Minutes | 4 | 0.7 |
| 08 | Information Gain | IG | 5.5 Minutes | 5 | 0.6 |
| 09 | Gain Ratio | GR | 6 Minutes | 6 | 0.5 |
| 10 | Wilcoxon Statistic | Wilcox | 3.5 Minutes | 3 | 0.8 |

**Table S23.** Runtime based analysis of gene selection methods in Fungal and insect stress datasets.

| **Sl. No.** | **Methods** | **Symbol** | **Run time** | **Ranks** | **Rank score** |
| --- | --- | --- | --- | --- | --- |
| 01 | BSM | BSM | 20 Min | 8 | 0.3 |
| 02 | SVM (RFE)-MRMR | SVM-MRMR | 1hr 25 Min | 10 | 0.1 |
| 03 | MRMR | MRMR | 10 Min | 7 | 0.4 |
| 04 | SVM with Recursive Feature Elimination | SVM-RFE | 1hr 20 Min | 9 | 0.2 |
| 05 | t-score | t | 1.5 Minutes | 1 | 1 |
| 06 | F-score | F | 2.5 Minutes | 2 | 0.9 |
| 07 | Pearson’s Linear Correlation | PCR | 3 Minutes | 4 | 0.7 |
| 08 | Information Gain | IG | 4 Minutes | 5 | 0.6 |
| 09 | Gain Ratio | GR | 5 Minutes | 6 | 0.5 |
| 10 | Wilcoxon Statistic | Wilcox | 3 Minutes | 3 | 0.8 |

Document S9: Guidelines and Tutorials for BSM

R Package Inputs for BSM R Package

1. Gene Expression matrix ***X*** with *N* genes and *M* samples
2. Class information *M*-dimensional vector having elements +1 (case) and -1 (control)
3. Method for integrating SVM and MRMR weights. It can be either Linear or Quadratic.

Linear method: Linear combination of weights computed through MRMR, and SVM methods and is given as:

| ${SL}_{i}={\beta w}_{i}+\left( 1-\beta\right){\vert k}_{i}\vert$ | (7) |
| --- | --- |

Quadratic method: The quadratic method for integration of weights can be expressed as:

| ${SD}_{i}=\frac{{\beta\gamma}_{i}^{MR}{w_{i}}^{norm}+(1-\beta)\gamma_{i}^{SV}\left\vert k_{i} \right\vert^{norm}}{\beta\gamma_{i}^{MR}+(1-\beta)\gamma_{i}^{SV}}$ | (8) |
| --- | --- |

where, $w_{i}$ and $k_{i}$ are MRMR and SVM weights for *i^th^* gene respectively; $\gamma_{i}^{MR}$and $\gamma_{i}^{SV}$ are ranks MRMR and SVM weights for *i^th^* gene respectively; ${w_{i}}^{norm}=\left( w_{i}-\min_{i} w_{i})/(\max_{i} w_{i}-\min_{i} w_{i} \right),$ $\left| k_{i} \right|^{norm}=(\left| k_{i} \right|-\min_{i} \left| k_{i} \right|)/(\max_{i} \left| k_{i} \right|-\min_{i} \left| k_{i} \right|)$, and $\beta\in\left( 0, 1 \right)$: integration constant.

1. A scalar representing trade-off between SVM and MRMR weights.
2. Number of bootsrap samples to be drawn from the data using simple random sampling with replacement (Bootstrap) procedure.
3. A method used for multiple hypothesis correction and computation of adjusted p-values. It can be any method out of "holm", "hochberg", "hommel", "bonferroni", "BH", "BY", "fdr".
4. Level of significance value (α) to decide the statistical significance of relevance of genes.

R package Installation from local directory:

Required e1071 R package

install.packages("…/…path location to the file…/BSM_0.1.0.tar.gz", repos = NULL, type = "source")

library(e1071)

library(BSM)

**** Data example

##Simulation of gene expression data

x <- as.data.frame(matrix(runif(1000), 50))

row.names(x) <- paste("Gene", 1:50)

colnames(x) <- paste("Samp", 1:20)

head(x) #######x is gene expression data matrix with 50 rows (genes) and 20 columns (samples)

y <- as.numeric(c(rep(1, 10), rep(-1, 10)))

y #####class labels for the samples (1: case and -1: control)

R package Installation steps from web:

#get the devtools

install.packages("devtools") ##install if not

iibrary(devtool)

#install BSM R package from web

install_github("sam-uofl/BSM")

library(BSM)

# Computation weights for gene selection through BSM method.

> bootsvmmrmrwt(x, y, method = “Linear”, beta = 0.5, nboot = 50)

# Computation statistical significance values for gene selection through Bootstrap-Support Vector Machine (SVM)-Maximum Relevance and Minimum Redundancy (MRMR) methods.

> pvalsvmmrmr(x, y, method, beta, nboot, p.adjust.method, plot)

# Selects the top ranked (differentially expressed) genes through Bootstrap-SVM-MRMR method.

> TopGenesBootSVMRMR(x, y, method = “Linear”, beta = 0.5, nboot = 50, n = 20)

# Selects the top ranked (differentially expressed) genes through MRMR method.

> TopGenesMRMR(x, y, n = 20)

# Selection of genes based on statistical significance values computed through BSM method.

> TopGenesPvalSVMRMR(x, y, method = “Linear”, beta = 0.5, nboot = 50, p.adjust.method = “BH”, n = 20)

# Selection of top ranked (differentially expressed) genes through SVM method

> TopGenesSVM(x, y, n = 20)

# Selects the top ranked (differentially expressed) genes through SVM-MRMR method.

> TopGenesSVMRMR(x, y, method = “Linear”, beta = 0.5, n =20)

#Computation of gene ranking weights through MRMR method.

> weightmr(x, y)

#Computation of weights for gene selection through SVM algorithm

> weightsvm(x, y)

#Computation weights for gene selection through SVM-MRMR method.

> weightsvmmrmr(x, y, method, beta)

**
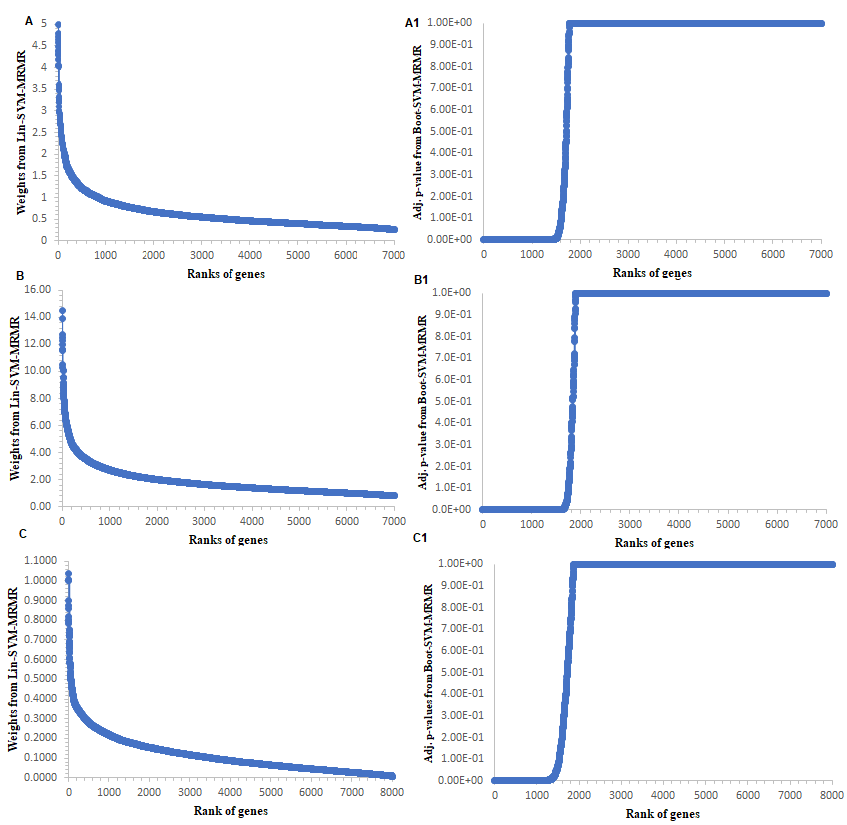
Figure Figure S3.** Graphical analysis of the proposed Boot-SVM-MRMR approach with Lin-SVM-MRMR approach for biotic stress datasets. Distribution of gene weights computed from SVM-MRMR approach for the biotic stresses. The distributions of weights from the SVM-MRMR are shown for (A): Bacterial; (B): Fungal; and (C): Insect stress datasets in rice. Distribution of adjusted gene *p-values* computed from for (A1): Bacterial; (B1): Fungal; and (C1): Insect stress datasets in rice BSM approach for the biotic. stresses. The distributions of adjusted *p-values* are shown
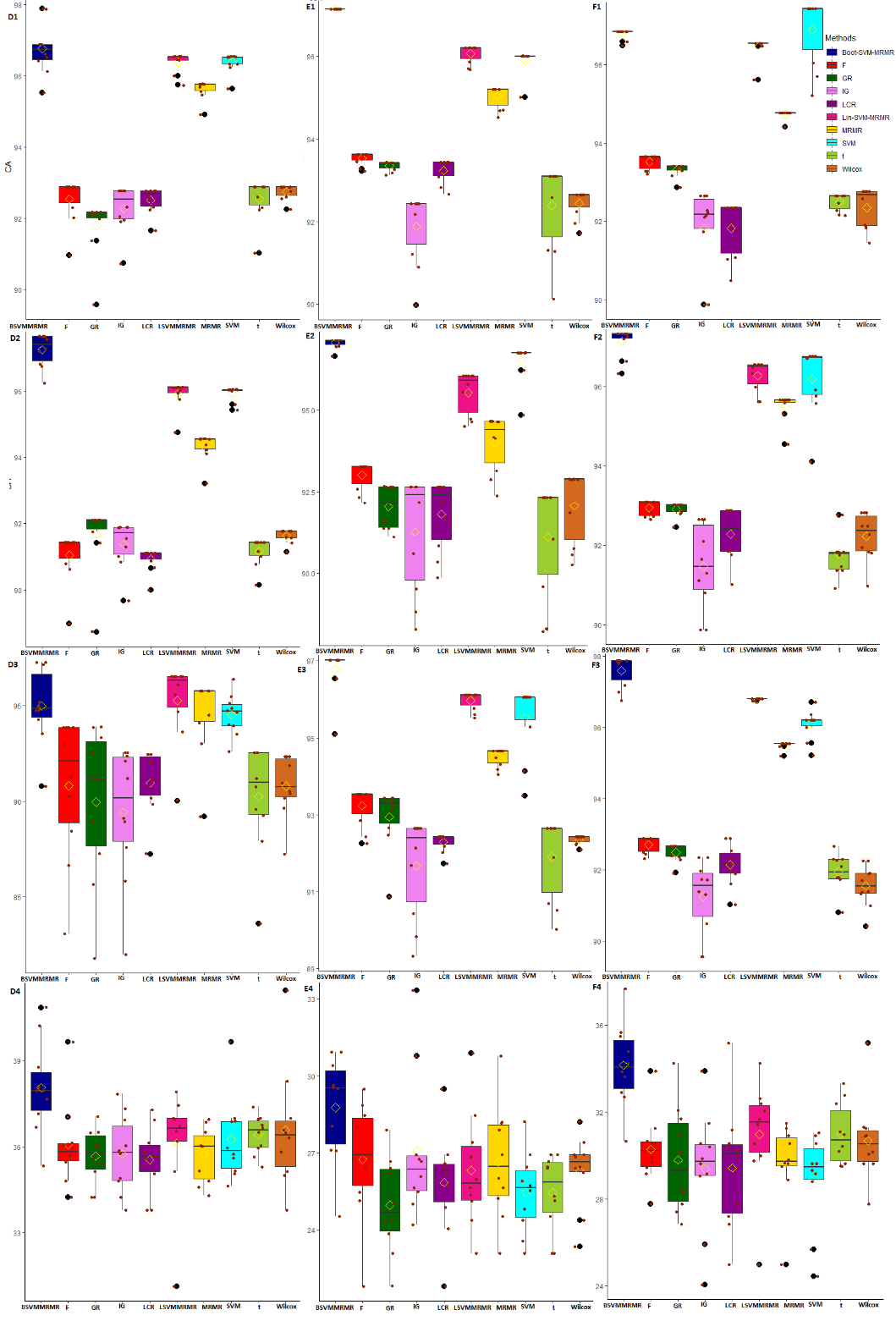
.

**Figure S4.** Classification based comparative performance analysis of gene selection methods in biotic stress datasets. The horizontal axis represents the gene selection methods. The vertical axis represents post selection classification accuracy obtained by using varying sliding window size technique. The classification accuracies over the window sizes are presented as boxes. The bars on the boxes represents the standard errors. The distributions of classification accuracies are shown for rice Bacterial stress dataset with SVM-LBF classifier (D1), SVM-PBF classifier (D2) SVM-RBF classifier (D3), and SVM-SBF classifier (D4); The distributions of classification accuracies are shown for rice Fungal stress dataset with SVM-LBF classifier (E1), SVM-PBF classifier (E2) SVM-RBF classifier (E3), and SVM-SBF classifier (E4) classifiers; The distributions of classification accuracies are shown for rice Insect stress dataset with SVM-LBF (F1), SVM-PBF (F2), SVM-RBF (F3), and SVM-SBF (F4) classifiers.
