# Supplementary material for: Statistical Approach for Biologically Relevant Gene Selection from High-Throughput Gene Expression Data
Source: Entropy (Basel). 2020 Oct 25;22(11):1205. doi: 10.3390/e22111205 (PMC7712650; doi:10.3390/e22111205)
Supplement: Supplementary file 1 [file entropy-22-01205-s001.zip › entropy-942531-supplementary/Supplemtary Information.pdf]

Document S1. Data Collection, Pre-Processing, Meta-Analysis and Preliminary Gene Selection to Prepare the Data for Performance Analysis of Gene Selection Methods.

Data Selection

The six different gene expression datasets for rice related to a balanced set of three abiotic stresses (salinity, cold and drought) and three biotic stresses (*Xanthomonas* bacteria, fungal and insect) were obtained from Gene Expression Omnibus (GEO) database of NCBI (http://www.ncbi.nlm.nih.gov) with platform GPL2025, as this platform contains as much as 220 microarray experiments (series) comprising 3480 samples/subjects of *Oryza sativa* L. as compared to other platforms. Among these 3480 samples, 550 experimental samples related to these six different biotic and abiotic stresses for rice were taken in this study. Further, the raw .CEL files for these gene expression samples are downloaded for GEO database of NCBI.

Data Preprocessing

The preprocessing of the gene expression datasets was done to remove noises, including missing and mislabeled probes (Das et al., 2017). Here, the preprocessing of data was conducted by using Bioconductor platform of R (Gentleman et al., 2004). Initially, the raw CEL files of the collected samples were processed using Robust Multichip Average (RMA) algorithm available in *affy* Bioconductor package of R (Gautier et al., 2004; Bolstad et al., 2003). This RMA procedure involves background correction, quantile normalization and summarization by median polish approach. Further, the log2 scale transformed expression data from RMA for the collected experimental samples were used for meta-analysis to remove the outlier samples. After normalization of the data, we used the z-score method, a Location-Scale approach (Lazar et al. 2013) to remove the batch effects of the genes. Here, the main idea behind the use of z-score method is to transform the data from each batch to have similar (equal) z-score (i.e. function of mean and variance) for each gene. It is assumed that these transformations, while trivially making data more comparable, do not remove any biological signal of interest

Meta-analysis of gene expression datasets

Meta-analysis was performed on the collected gene expression samples to remove the unusual or outlier samples from the data. It was performed individually for each stress. Through this, the gene expression samples with mean ≥ μ_0 and standard deviation ≤ σ_0 are retained and other samples, which do not satisfy this condition are considered as outliers and removed. Further, we validated the homogeneity of the selected samples through correlation analysis, as they were generated over varying experimental conditions. The parameters for meta-analysis in each stress condition are given in Table S2.

Table S2. Parameters for gene expression samples selection.

Sl. No.	Stress condition	Mean (μ_0)	Standard deviation (σ_0)	Samples selected
1	Salinity stress	5.7	2.6	45
2	Cold stress	6.5	2.7	28
3	Drought stress	6.2	2.6	70
4	Bacterial stress	6.8	2.5	74
5	Fungal stress	6.05	2.8	26
6	Insect stress	7.4	2.4	18

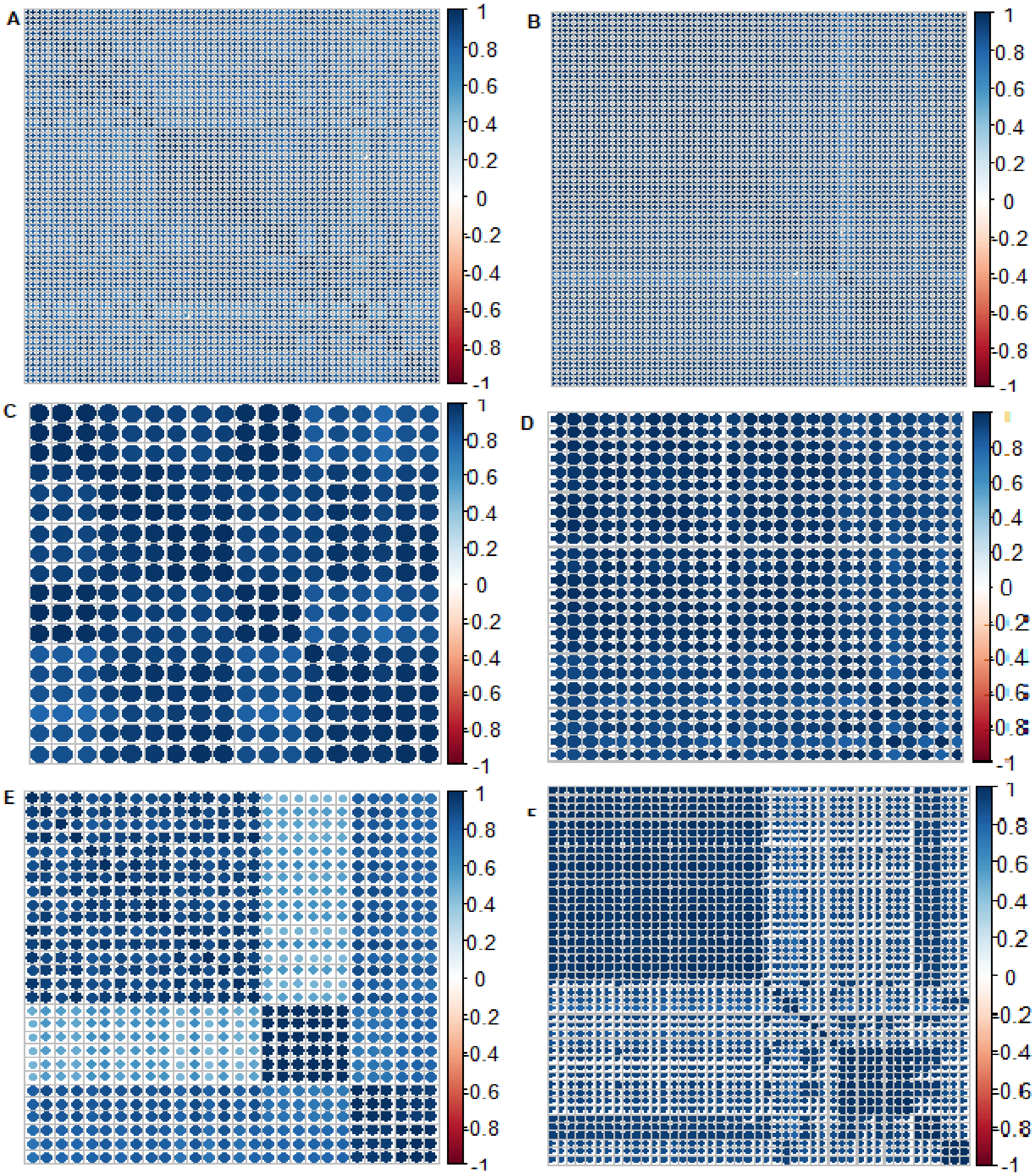

Figure S1. Correlation plot of the micro-array experimental samples under salinity stress for rice.

Through this meta-analysis, samples which satisfy the above condition are selected for each stress. These parameter combinations are chosen for each stress, as we observed uniformity of color in the correlation plot at these parameter settings, though they are generated over varying experimental conditions across the globe. For instance, in salinity stress, experimental samples whose mean (μ) ≥ 5.23 and standard deviation ≤ 2.51 were retained for further analysis as they are observed to be highly homogeneous at these parameters setting, irrespective of their experimental conditions (Figure S1). Similar interpretations can be made for cold, drought, bacterial, fungal and insect stresses in rice.

Preliminary Gene Selection for Dimension Reduction

The gene expression data generated from Affymetrix Rice Genome Array (GPL 2025 in GEO), which contains 57,381 probes and each probe is assumed to represent an individual gene. Further, there are 123 probe sets designed for control (in GPL 2025), so, we removed these probes from the analysis and the dataset on 57,258 valid probe sets were obtained for further analysis. It would be of high computational complex as well as statistically infeasible to use the gene set selection methods directly on expression data on 57,258 probes. Hence, we first employed t-test and Fold Change (FC) criteria to filter out unlikely genes to reduce the dimension of the GE datasets at the preliminary stage. In our preliminary selection, we assigned 1 and 0.05 as the |FC| and *p-value* thresholds respectively, resulting in selection of several thousands of genes (Table 1). Further, GE data on these selected genes (at the preliminary stage) were further used for performance analysis of proposed and existing gene selection techniques.

References

1. Das S, Meher PK, Rai A, Bhar LM, Mandal BN. Statistical approaches for gene selection, hub gene identification and module interaction in gene co-expression network analysis: An application to Aluminum stress in Soybean (*Glycine max* L.). PLoS ONE. 2017, 12(1): e0169605. doi:10.1371/journal.pone.0169605.
2. Gentleman RC, Carey VJ, Bates DM, Bolstad B, Dettling M, Dudoit S, Ellis B, Gautier L, Ge Y, Gentry J (2004) Bioconductor: open software development for computational biology and bioinformatics. Genome Biol 5: 80.
3. Gautier L, Cope L, Bolstad BM, Irizarry RA (2004) Affy—analysis of Affymetrix GeneChip data at the probe level. Bioinformatics 20: 307–315.
4. Irizarry RA, Hobbs B, Collin F, Beazer-Barclay,YD, Antonellis KJ, Scherf U, Speed TP (2003) Exploration, normalization, and summaries of high density oligonucleotide array probe level data. Biostatistics 4: 249–264.
5. Bolstad B, Irizarry R, Astrand M, Speed T (2003) A comparison of normalization methods for high density oligonucleotide array data based on bias and variance. Bioinformatics 19(2): 185–193.

Document S2: Stress(S) Specific Quantitative Trait Loci Information for Rice (*Oryza Sativa L.*)

The list of trait specific Quantitative Trait Loci (QTL) for the abiotic, *viz*. salinity, drought, and cold, and biotic stresses bacteria (*Xanthomonas*), fungal (blast) and insect (brown plant hopper) for rice were collected from the Gramene QTL database (http://www.gramene.org/qtl/) (Ni et al. 2009). Then, the genomic regions of these QTLs (for each stress) were mapped to rice genome using Gramene annotation of rice genome of MSU Rice Genome Annotation (Osa1) Release 6 (Ouyang et al. 2007). For a given QTL, there may be 25–30 genes per cM (~270 kbp in rice) (Khurana and Gaikwad, 2005). Further, the lists of the QTLs for each of the six different stresses are given in Table S2-S6.

Table S3. List of salinity responsive QTLs in rice (*Oryza sativa L.*).

Sl. No.	QTL ID	Chr. No.	Start	End	Note
1	AQEM001	1	33956950	37713775	
2	AQEM006	1	9820009	11232822	
3	AQGR001	1	38530957	38531467	
4	AQGR002	3	22798284	22830744	
5	AQCL001	3	484860	485333	
6	AQEM009	4	19928370	22355854	
7	AQCL002	4	33663984	33664487	
8	AQCL003	5	18874932	18875558	
9	AQCL004	6	22862400	22862821	
10	AQEM002	6	21605889	24919236	
11	AQEM003	7	4573316	7739951	
12	AQEM004	7	2633784	4575215	
13	AQEM007	9	14362062	17837010	
14	AQEM005	1	33956950	37713775	Same as 1
15	AQEM008	7	2633784	4575215	Same as 12
16	AQEM010	7	2633784	4575215	Same as 12
17	AQEM011	7	2633784	4575215	Same as 12

Sl. No.: Serial number of the QTL; QTL ID: Published qtl id; Chr. No.: Chromosome number of the QTL; Start: start position of the QTL in terms of base pairs (bp); End: end position of the QTL in terms of length of bps.

Table S4. List of cold responsive QTLs for rice (*Oryza sativa L.*).

Sl. No.	QTL ID	Chr. No.	Start	End	Note
1	CQAA8	4	688353	6574518	
2	AQDU008	4	688353	6574518	Same as 1
3	AQDU004	11	932068	932221	
4	CQP8	11	1491600	2523808	
5	AQDU009	6	5425408	5425631	
6	CQAA9	6	5425408	5425631	Same as 5
7	AQAV003	1	5558576	7445919	
8	AQDU014	12	8826555	8826855	
9	AQDU005	12	8826555	8826855	Same as 8
10	AQAV004	2	11389704	12216613	
11	AQAV001	2	11389704	12216613	Same as 10
12	AQAV002	9	17719660	18810331	
13	AQF129	8	19051713	22886866	
14	AQDU002	6	19499320	27252383	
15	AQDU003	8	20650060	21142502	
16	AQDU012	8	20650060	21142502	Same as 15
17	AQDU010	8	21142348	21142502	Same as 15
18	CQAA10	8	21142348	21142502	Same as 15
19	CQP7	7	22857717	22885543	
20	AQDU013	11	25153466	25153681	
21	AQDU015	4	26857374	29061127	
22	AQBO001	7	27159051	27159261	
23	AQAV006	5	27342022	27342124	
24	CQO3	4	29155838	30445683	
25	AQDU001	4	30772388	32650528	
26	CQO1	4	31276528	32772351	
27	CQAA6	1	32099566	33677892	
28	AQDU006	1	32099566	33677892	Same as 27
29	CQP1	1	34651088	39949610	

Sl. No.: Serial number of the QTL; QTL ID: Published qtl id; Chr. No.: Chromosome number of the QTL; Start: start position of the QTL in terms of base pairs (bp); End: end position of the QTL in terms of length of bps.

Table S5. List of drought responsive QTLs in rice (*Oryza sativa L.*).

Sl. No.	QTL ID	Chr.	Start	End	Note
1	CQAI48	4	13634515	13635012	
2	AQA046	12	26017140	27489485	
3	AQA045	4	13634515	13635012	Same as 1
4	CQAI49	12	26017140	27489485	Same as 2
5	AQHP062	8	21645663	21647445	
6	AQHP058	2	10503368	10503846	
7	AQHP059	4	31662839	31663326	
8	AQHP082	6	6718648	9537772	
9	AQHP083	7	13074864	13075056	
10	AQHP070	4	31662839	31663326	Same as 7
11	AQHP065	1	29184260	29184844	
12	AQHP081	4	8610617	8611256	
13	AQHP079	3	15469002	19412007	
14	AQAN005	8	20094533	20094695	
15	AQAN004	4	8610617	8611256	Same as 12
16	AQHP066	2	29761981	29762453	
17	AQHP080	4	31662839	31663326	Same as 7
18	AQHP084	8	21645663	21647445	Same as 5
19	AQHP069	3	22798284	35828040	
20	AQHP068	2	10503368	19866086	
21	AQAN001	5	27342022	28610866	
22	AQHP078	11	4413928	4415836	
23	AQHP067	2	27034342	27035328	
24	AQHP085	9	20481606	20482133	
25	AQHP061	6	2560318	2561213	
26	AQHP075	9	20481606	20482133	Same as 24
27	AQHP060	4	8610617	8611256	Same as 12
28	AQHP074	8	21645663	21647445	Same as 5
29	AQHP087	11	19565059	19565672	

Sl. No.: Serial number of the QTL; QTL ID: Published qtl id; Chr.: Chromosome number of the QTL; Start: start position of the QTL in terms of base pairs (bp); End: end position of the QTL in terms of length of bps.

Table S6. List of bacterial responsive QTLs in rice (*Oryza sativa L.*).

Sl. No.	QTL ID	Chr.	Start	End	Note
1	AQW001	2	10503368	10503846	
2	AQW002	3	15469002	15472481	
3	AQBT021	10	21098188	21099881	
4	AQBT020	10	21098188	21099881	Same as 3
5	AQBT022	10	21098188	21099881	Same as 3
6	AQBT019	9	22194746	22196064	
7	AQBT017	9	22194746	22196064	Same as 6
8	AQBT018	9	22194746	22196064	Same as 6
9	AQBT006	3	23088332	23088721	
10	AQBT007	3	23088332	23088721	Same as 9
11	AQBT005	3	23088332	23088721	Same as 9
12	AQW003	3	23088332	28232092	
13	AQW004	5	24261900	24262670	
14	AQW006	8	27822512	27825271	
15	AQBT023	11	28409788	28412347	
16	AQBT024	11	28409788	28412347	Same as 16
17	AQBT025	11	28409788	28412347	Same as 16
18	AQBT008	4	31662839	31663326	
19	AQBT010	4	31662839	31663326	Same as 18
20	AQBT009	4	31662839	31663326	Same as 18
21	AQBT001	2	33849901	35662199	
22	AQW005	7	6779215	6779821	
23	AQBT029	12	7729365	21041065	
24	AQBT030	12	7729365	21041065	Same as 23
25	AQBT002	3	718777	1450227	
26	AQBT003	3	718777	1450227	Same as 26
27	AQBT004	3	718777	1450227	Same as 26
28	AQBT013	4	8610617	11234543	
29	AQBT012	4	8610617	11234543	Same as 28
30	AQBT011	4	8610617	11234543	Same as 28
31	AQBT014	8	8923052	8924004	
32	AQBT016	8	8923052	8924004	Same as 31
33	AQBT015	8	8923052	8924004	Same as 31

Sl. No.: Serial number of the QTL; QTL ID: Published qtl id; Chr.: Chromosome number of the QTL; Start: start position of the QTL in terms of base pairs (bp); End: end position of the QTL in terms of length of bps.

Table S7. List of blast (fungal) responsive unique QTLs in rice (*Oryza sativa L.*).

Sl. No.	QTL ID	Chr.	Start	Stop
1	AQAF003	1	1,98,822	18,91,260
2	AQEN002	1	50,94,276	1,10,77,990
3	AQAH002	1	2,41,86,290	2,91,84,844
4	AQEN079	1	50,94,276	50,95,699
5	AQGJ001	1	2,94,46,995	2,94,47,853
6	AQAF011	1	3,10,46,003	3,10,47,458
7	AQAF013	1	3,30,53,493	4,00,65,325
8	AQAF015	1	3,44,70,620	4,00,65,325
9	AQAF017	1	3,44,70,620	3,77,13,775
10	AQAQ008	1	3,07,37,705	4,05,67,354
11	AQAF006	1	74,45,627	7445919
12	AQEN001	1	1,46,20,467	3,49,40,769
13	AQAF007	1	79,70,722	79,70,839
14	AQCT001	1	47,38,488	3,01,70,285
15	AQEN011	1	1,46,20,467	2,17,01,719
16	AQEN051	1	2,05,98,332	2,05,99,810
17	AQCT002	2	3,56,61,689	3,56,62,199
18	AQAQ001	2	3,56,61,689	3,56,62,199
19	AQAF026	2	3,46,52,316	3,51,36,068
20	AQEN069	2	2,74,82,581	3,11,07,173
21	AQEN070	2	2,74,82,581	27,483,257
22	AQEN039	3	4,84,860	4,85,333
23	AQEN012	3	4,84,860	1,450,227
24	AQEN003	3	4,84,860	34,96,275
25	AQAQ020	3	2,51,28,239	25,128,864
26	AQAF029	3	2,30,88,332	2,45,95,466
27	AQGJ003	4	86,10,617	86,11,256
28	AQEN063	4	3,16,62,839	3,16,63,326
29	AQAQ015	4	86,10,617	1,12,34,543
30	CQAC1	4	1,99,28,370	2,23,55,854
31	AQEN061	4	2,00,87,103	2,00,87,362
32	AQAQ024	5	2,25,79,390	2,25,80,355
33	AQCT003	5	20,91,276	2,782,394
34	AQEN041	6	23,63,670	23,63,704
35	AQEN005	6	23,63,670	67,20,901
36	AQAQ021	6	2,90,27,995	3,09,45,628
37	AQEN014	6	95,36,259	2,44,55,212
38	AQGJ023	6	25,60,318	62,84,636
39	AQEN059	6	95,36,259	95,37,772
40	AQGJ008	6	2,67,07,816	26,708,549
41	AQCT004	6	62,83,401	6,928,661
42	AQAH001	6	69,27,624	6,928,661
43	AQEN044	7	2,94,66,368	2,94,67,498
44	AQEN007	7	1,30,74,864	29,467,498
45	AQAF030	7	15,36,133	2,317,976
46	AQEN033	7	15,36,133	1,537,879
47	AQAF033	7	2,54,72,688	2,65,29,185
48	AQGJ010	7	2,67,04,922	29,467,498
49	AQAQ016	7	1,75,25,817	18,686,761

50	AQAF031	7	23,16,691	7,232,998
51	AQGJ026	7	71,24,042	7,124,718
52	AQAF035	8	41,05,519	53,27,118
53	AQEN015	8	41,05,519	1,74,38,003
54	AQAF034	8	41,05,519	41,06,001
55	AQEN037	8	41,05,519	4,106,001
56	AQAF038	9	1,46,48,372	20,174,430
57	CQAC3	9	96,29,362	10,801,158
58	AQAQ022	9	12,71,123	10,801,158
59	AQAF040	9	1,77,19,660	18,810,331
60	AQAF041	9	1,99,46,740	20,482,185
61	AQGJ027	9	1,88,10,067	1,88,10,331
62	AQAF042	9	2,11,89,110	22,196,064
63	AQCT006	10	2,09,76,812	20,978,165
64	AQEN067	11	2,03,36,572	20,337,612
65	AQEN016	11	1,81,78,768	20,337,612
66	AQAQ017	11	1,36,71,613	28,412,347
67	AQEN081	11	1,81,78,768	18,179,510
68	AQAO001	11	1,78,08,335	22,816,523
69	AQGJ013	11	66,86,166	6,687,145
70	AQCT007	11	46,24,598	46,26,888
71	AQAQ009	12	5,32,909	1,595,325
72	AQEN017	12	77,29,365	23,775,487
73	AQEN047	12	77,29,365	7,729,855
74	CQAC4	12	1,10,58,522	18,867,702
75	AQCT008	12	77,29,365	13,429,507
76	AQAQ011	12	77,29,365	77,29,855
77	AQEN072	12	15,94,823	15,95,325

Sl. No.: Serial number of the unique QTL; QTL ID: Published qtl id; Chr. No.: Chromosome number of the QTL; Start: start position of the QTL in terms of base pairs (bp); End: end position of the QTL in terms of length of bps.

Table S8. List of brown plant hopper (insect) responsive unique QTLs in rice (*Oryza sativa L.*).

Sl. No.	QTL ID	Chr.	Start	Stop
1	AQAP053	2	7,44,663	7,45,178
2	AQAP027	6	17,64,586	18,22,651
3	CQAM3	1	4,29,53,262	42,955,596
4	AQAP058	10	53,52,766	1,58,02,326
5	AQAP009	8	1,39,27,893	2,28,86,866
6	AQBA009	6	67,18,648	67,20,901
7	AQBA003	8	2,78,22,512	2,78,25,271
8	AQBA005	4	3,36,63,984	3,46,98,383
9	AQW015	12	2,74,88,270	2,74,89,485
10	AQW012	11	2,31,54,725	2,31,55,291
11	AQBA002	6	67,18,648	6,720,901
12	AQAP023	5	1,88,74,932	22,580,355
13	AQAP040	6	1,70,54,655	17,055,184
14	AQAP032	10	2,10,98,188	2,10,99,881
15	AQAP054	3	32,36,247	3,236,745
16	AQAP048	4	3,06,30,093	3,06,30,917
17	AQAP036	12	2,61,07,904	26,992,979
18	AQAP050	11	1,36,71,613	2,31,55,291
19	AQAP051	2	2,58,65,334	27,610,063
20	AQAP043	5	2,25,79,390	29,285,656
21	AQAP018	8	2,28,85,196	26,282,308
22	AQAP035	9	2,21,94,746	2,21,96,064
23	AQAP042	9	2,21,94,746	22,196,064
24	AQAP029	6	39,22,784	26,708,549
25	AQW010	6	67,18,648	19,338,095
26	AQBA020	6	67,18,648	1,93,38,095
27	AQAP015	1	2,76,25,475	2,91,84,844
28	AQAP028	8	89,23,052	8,924,004
29	CQAM2	6	41,60,454	8,066,358
30	AQW007	3	57,29,669	7,350,653
31	AQAP004	3	3,03,13,472	3,03,15,075
32	CQT1	4	35,46,753	14,707,274
33	AQAP030	11	1,36,71,613	28,412,347
34	AQAP045	1	3,96,87,395	4,05,67,354
35	AQAP005	5	52,55,880	52,56,140
36	AQBA017	1	1,46,20,467	14,626,881
37	AQAP056	6	31,68,314	54,25,631
38	AQAP057	7	1,75,25,817	2,57,75,868
39	AQAP039	1	10,39,086	10,39,868
40	AQBA007	2	1,98,65,083	2,45,66,182
41	AQAU001	2	89,84,645	18,249,617
42	CQT2	3	3,19,45,962	35,710,936
43	AQW013	12	1,96,28,443	19,628,925
44	AQAP034	11	57,06,417	57,06,935
45	AQAP041	2	45,24,663	5,263,536
46	AQAP055	4	2,01,71,917	2,01,73,040
47	AQW014	12	1,96,28,443	19,628,925
48	AQBA022	1	1,46,20,467	14,626,881
49	AQW008	4	2,41,65,104	24,165,408

50	AQAU003	12	5,32,909	5,33,313
51	AQAP007	5	52,55,880	6,700,408
52	AQAU002	10	2,14,02,080	23,031,714
53	AQAP031	8	1,39,27,893	2,06,50,257
54	AQAP052	2	52,62,891	69,16,662
55	AQBA008	4	3,16,62,839	32,449,446
56	AQAP046	10	1,77,94,267	1,98,23,295
57	AQAP001	1	97,01,793	1,04,91,821

Sl. No.: Serial number of the unique QTL; QTL ID: Published qtl id; Chr. No.: Chromosome number of the QTL; Start: start position of the QTL in terms of base pairs (bp); End: end position of the QTL in terms of length of bps.

Document S3: Objective Function of Support Vector Machine

Consider $\{\boldsymbol{x}_m, y_m\}$ be the GE data for m^{th} sample (m = 1, 2, …, M). $\{\boldsymbol{x}_m, y_m\} \in R^N \text{x} \{-1, 1\}$ given as input to Support Vector Machine (SVM). Here, we wish to find out a hyperplane that divides the GE samples/subjects, *i.e.* $\boldsymbol{x}_m$ for case class ($y_m = 1$) from the GE samples/subjects for control class ($y_m = -1$) in such a way that the distance between the hyperplane and the point, *i.e.* $\boldsymbol{x}_m$ is maximum. Then the hyperplane can be written as:

$$\boldsymbol{k}.\boldsymbol{x_m} + b = 0 \qquad \forall\, m = 1, 2, \dots, M \tag{1}$$

where, $\boldsymbol{k}$ and b are the weight vector and bias respectively.

Here, we assume that the GE samples for two classes are linearly separable. In other words, we can select two parallel hyperplanes that separate the case and control classes in such a way that the distance between them is maximum.

Now, for case, the hyperplane becomes:

$$\boldsymbol{k}.\boldsymbol{x_n} + b = 1 \qquad \forall\, n = 1, 2, \dots, M_1 \tag{2}$$

For control, the hyperplane becomes:

$$\boldsymbol{k}.\boldsymbol{x_o} + b = -1 \qquad \forall\, o = 1, 2, \dots, M_2 \tag{3}$$

Here, weight vector is chosen in such a way that the distance between two the hyperplanes, i.e. Eq. (2) – Eq (3), is maximum. Mathematically:

$$\boldsymbol{k}.(\boldsymbol{x_n} - \boldsymbol{x_o}) = 2 \tag{4}$$

Dividing $\|k\|^2$ in both sides of Eq. 4, it becomes

$$\frac{\boldsymbol{k}}{\|k\|^2}.(\boldsymbol{x_n} - \boldsymbol{x_o}) = \frac{\boldsymbol{2}}{\|k\|^2} \tag{5}$$

So, to maximize the distance between the planes in Eq. 5, we need to minimize $\frac{\|k\|^2}{2}$. Hence, the objective function, J, for this case vs. control classification problem becomes (Guyon et al., 2003):

$$J = \|k\|^2/2 \tag{6}$$

Through Taylor series expansion, the objective function, J, can be approximated as (at k=c):

$$J = \frac{1}{2}\left\{\|c\|^2 + \frac{\partial}{\partial k}\|k\|^2{}_{k=c}(k-c) + \frac{1}{2!}\frac{\partial^2}{\partial k^2}\|k\|^2{}_{k=c}(k-c)^2 + \frac{1}{3!}\|k\|^2{}_{k=c}(k-c)^3 + \cdots\right\}$$

$$= \frac{1}{2}\left\{\|c\|^2 + \frac{\partial J_2}{\partial k}_{k=c}(k-c) + \frac{1}{2!}\frac{\partial^2 J_2}{\partial k^2}_{k=c}(k-c)^2 + \frac{1}{3!}\frac{\partial^3 J_2}{\partial k^3}_{k=c}(k-c)^3 + \cdots\right\}$$

Differentiating both sides with respect to k, and ignoring the second and higher order derivatives terms from the expression, we have

$$\frac{\partial J}{\partial k} = \frac{1}{2}\left\{0 + \frac{\partial J}{\partial k}\left(\frac{\partial J}{\partial k}_{k=c}(k-c)\right)\right\}$$

$$\frac{\partial J}{\partial k} = \frac{1}{2}\left\{(k-c)\frac{\partial^2 J}{\partial k^2} + \frac{\partial J}{\partial k}.1\right)\right\}$$

$$\frac{\partial J}{\partial k} = \frac{1}{2}\frac{\partial^2 J}{\partial k^2}(k-c)$$

Replace k with Δk in above expression and ignoring the constant c. Now, the above expression becomes, as below.

$$\lim_{\Delta k}\frac{\Delta J}{\Delta k} = \frac{1}{2}\frac{\partial^2 J}{\partial k^2}(\Delta k)$$

$$\Delta J = \frac{1}{2}\frac{\partial^2 J}{\partial k^2}(\Delta k)^2$$

Further, ΔJ attributed to i^{th} gene can be expressed as:

$$\Delta J(i) = \frac{1}{2}\frac{\partial^2 J_2}{\partial {k_i}^2}(\Delta k_i)^2$$

Here, it may be noted that the J is directly proportional to ${k_i}^2$. Hence, measurement of either k_i or J provides equivalent information. Keeping this in view, ${k_i}^2$ is used as the ranking criterion for evaluating impact of ith gene on this classification.

Reference

1. Guyon I, Weston J, Barnhill S, Vapnik V. Gene selection for cancer classification using support vector machines. Mach Learn. 2002. doi:10.1023/A:1012487302797

Document S4: Determination of 'Beta' for Quadratic Integration

The value of *beta* for computing the score for quadratic integration of SVM with MRMR method was determined empirically from the set {0.1, 0.2, 0.3, 0.4,0.5,0.6, 0.7, 0.8, 0.9}. The classification accuracies were computed based on the fivefold cross-validation performance. Further, the classification accuracies were plotted against beta values to find the optimum value of beta for integrating SVM and MRMR scores through quadratic integration. Here, the above procedure is repeated to obtain optimal beta value for different gene sets, *i.e.* 10, 20, 50, 100, 150 and 200.

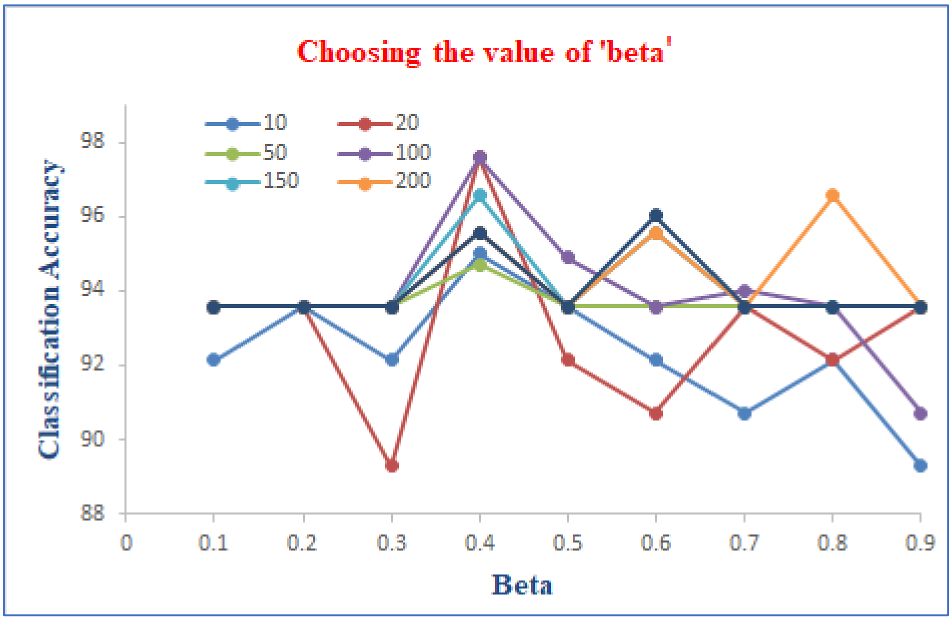

Figure S2. Choosing the optimal value of beta for quadratic integration of SVM and MRMR scores. The horizontal axis represents the value of beta. The vertical axis represents the classification accuracy computed over five-fold cross validation through Support Vector Machine-Linear Basis Function classifier. The line plots are shown for selected gene sets with size (A) 10, (B) 20, (C) 50, (D) 100, (E) 150 and (F) 200.

The classification accuracies obtained through training Support Vector Machine-Linear Basis Function classifier over fivefold cross-validations for different beta values are shown in Figure S1. It is found that for gene set of size 10, the maximum classification accuracy is achieved for the beta value of 0.4. Similar observations are found for other gene set of sizes, *e.g.* 20, 50, 100, 150 and 200. Therefore, we can conclude that the optimal value of beta for the quadratic integration of SVM and MRMR scores for the given dataset is 0.4. It may be worthy to note that, the value of beta is highly dependent and need to be estimated from the data itself prior going to final score integration.

Document S5. List of Gene Selection Methods for Performance Analysis.

The comparative performance analysis of the proposed Boot-SVM-MRMR gene selection approach was carried out with respect to nine existing gene selection techniques on multiple (six different) crop gene expression datasets based on different statistically necessary and biological relevant criteria. The list of the methods used for performance analysis are given in Table S9.

Table S9. Gene selection methods used in the comparative performance analysis.

Methods	Expression	R tools	References
BSM	Adjusted *p-value* from BSM	BSM	Proposed
SVM-MRMR	$\delta w_i + (1-\delta)\lvert k_i\rvert$	*	[11]
MRMR	$w_i = \max_{i\in\Omega}\{F(i,y)/(\frac{1}{N}\sum_{\substack{j=1 \\ j\neq i}}^{N}\lvert C(i,j)\rvert)\}$	*	[1, 2]
SVM-RFE	$k = \sum_{m=1}^{M} \varphi_m y_m x_{\cdot m}$	e1071	[3, 4]
t-score	$t = \frac{\overline{x_1} - \overline{x_2}}{\sqrt{S^2(\frac{1}{n_1}+\frac{1}{n_2})}}$	Mass	[5, 6]
F-score	$F = \frac{(\overline{x_1} - \bar{x})^2 + (\overline{x_2} - \bar{x})^2}{S_1^2 + S_2^2}$	stats	[7]
Pearson's Correlation (PCR)	$r = \frac{S_{12}}{S_1 S_2}$	FSelector	[8, 9]
Information Gain (IG)	$H(c) + H(i) - H(c,i)$	FSelector	[9, 10]
Gain Ratio (GR)	$(H(c) + H(i) - H(c,i))/H(i)$	FSelector	[9, 10]
Wilcoxon Statistic (Wilcox)	$\frac{W - \frac{n(n+1)}{4}}{\sqrt{n(n+1)(2n+1)/24}}$	Mass	[12]

Codes: Codes for the methods; ***:** R-code written; BSM: proposed Bootstrap SVM-MRMR gene selection technique; SVM-MRMR: Linear SVM-MRMR; MRMR: Maximum Relevance Minimum Redundancy; SVM-RFE: Support Vector Machine Recursive Feature Elimination; t-score: t-statistic obtained from the t-test; F-score: Fisher's score; S^2: pooled variance; S^2_1: Sample variance for case group (with class labels as 1); S^2_2: Sample variance for control group (with class labels as -1); *H(.)*: Entropy measure

References

1. Ding C, Peng H. Minimum redundancy feature selection from microarray gene expression data. J. Bioinformatics Comput Biol. 2005, 3(2):185-205.
2. Peng H, Long F, Ding C. Feature selection based on mutual information: Criteria of max-dependency, max-relevance and min-redundancy. IEEE Trans Pattern Anal Mach Intell. 2005, 27 (8) 1226–1238.
3. Guyon I, Weston J, Barnhill S, Vapnik V. Gene selection for cancer classification using support vector machines. Machine Learning. 2002, 46: 389–422.
4. Liang Y, Zhang F, Wang J, Joshi T, Wang Y, Xu D. Prediction of drought-resistant genes in *Arabidopsis thaliana* using SVM-RFE. PLoS ONE. 2011, 6(7): e21750. doi:10.1371/journal.pone.0021750.
5. Cui X, Churchill G. Statistical tests for differential expression in cDNA microarray experiments. Genome Biol. 2003; 4 (4): 210.
6. Das S, Meher PK, Pradhan UK, Paul AK. Inferring gene regulatory networks using Kendall's tau correlation coefficient and identification of salinity stress responsive genes in rice. Current Science. 2017b, 112(6): 1257-1262.
7. Lazar C, Taminau J, Meganck S, Steenhoff D, Coletta A, Molter C, et al. A survey on filter techniques for feature selection in gene expression microarray analysis. IEEE/ACM Trans on Comput Biol and Bioinfo. 2012, 9(4): 1106–1119.
8. Golub T, Slonim D, Tamayo P, Huard C, Gaasenbeek M, Mesirov J, et al. Molecular classification of cancer: class discovery and class prediction by gene expression monitoring. Science. 1999, 286: 531-537.

9. Cheng T, Wang Y, Bryant SH. FSelector: a Ruby gem for feature selection. Bioinformatics. 2012, 28(21): 2851–2852. doi: 10.1093/bioinformatics/bts528.
10. Liu X, Krishnan A, Mondry A. An entropy-based gene selection method for cancer classification using microarray data. BMC Bioinformatics. 2005, 6:76.
11. Mundra PA, Rajapakse JC. SVM-RFE With MRMR Filter for Gene Selection. IEEE Trans Nanobioscience. 2010;9: 31–37. doi:10.1109/TNB.2009.2035284
12. Hossain A, Willan AR, Beyene J. An Improved Method on Wilcoxon Rank Sum Test for Gene Selection from Microarray Experiments. Commun Stat Simul Comput. 2013, 42 (7): 1563-1577. doi:10.1080/03610918.2012.667479.
13. Lazar, C. et al. Batch effect removal methods for microarray gene expression data integration: a survey, *Briefings in Bioinformatics* 2013, 14 (4) 469–490. doi.org/10.1093/bib/bbs037

Table S10. Different parameters combination for computation of classification accuracies.

Sl. No.	Gene set size (*n*)	Window size (*S*)	Sliding length (*L*)
1	10	5	1
2	20	5	2
3	50	10	5
4	100	50	20
5	150	100	20
6	200	150	20
7	500	200	20
8	1000	250	20
9	1200	300	20
10	1500	400	50

Gene set size: size of the gene sets; Window size: number of ranked genes taken from gene set at each step; Sliding length: length of sliding of each window at each subsequent step

<u>Document S6.</u> Mean Accuracy and Standard Error in Classification Computed through Varying Sliding Windows Size Technique.

Table S11. Mean and standard error in classification accuracy computed through varying sliding windows technique for salinity stress in rice.

	Gene sets									
**Methods**	**10**	**20**	**50**	**100**	**150**	**200**	**500**	**1000**	**1200**	**1500**
					**SVM-LBF classifier**					
**SVM-MRMR**	97.06	96.94	96.67	96.02	95.28	95.28	95.28	95.34	95.28	95.28
	0.44	0.36	0.41	0.74	0.56	0.54	0.67	0.85	0.56	0.46
**MRMR**	96.56	96.17	95.89	94.78	94.78	94.78	94.78	94.89	94.78	94.78
	0.44	0.41	0.42	0.46	0.42	0.39	0.23	0.47	0.57	0.59
**SVM-RFE**	97.56	97.72	96.33	95.04	95.04	95.78	96.22	96.60	96.67	96.69
	0.44	0.28	0.56	0.74	0.74	0.23	0.24	0.18	0.16	0.23
**Inf. Gain**	95.00	95.00	94.17	92.78	92.04	92.78	92.78	92.72	92.73	92.68
	0.46	0.59	0.41	0.45	0.74	0.56	0.23	0.32	0.49	0.30
**Wilcoxon test**	92.67	93.44	92.89	91.78	91.78	91.78	91.63	91.25	91.58	91.37
	0.54	0.36	0.42	0.45	0.57	0.32	0.48	0.43	0.49	0.44
**t-test**	92.56	93.00	92.17	90.04	90.78	90.78	90.48	90.49	90.33	90.27
	0.61	0.45	0.41	0.74	0.48	0.55	0.89	0.87	0.71	0.57
**PCR**	91.67	92.72	91.89	90.78	90.78	90.78	90.78	90.78	90.78	90.78
	0.54	0.28	0.56	0.56	0.66	0.48	0.66	0.81	0.47	0.57
**F-Score**	91.67	92.72	92.17	90.78	90.78	90.78	90.78	90.78	90.78	90.78
	0.54	0.28	0.41	0.53	0.58	0.52	0.31	0.51	0.58	0.51
**Gain Ratio**	93.00	93.00	93.00	91.52	90.78	90.04	89.15	90.08	90.23	90.37
	0.77	0.74	0.74	0.74	0.74	0.74	0.26	0.17	0.14	0.19
**BSM**	98.00	98.00	98.33	95.78	95.78	97.78	95.78	95.84	97.88	95.58
	0.32	0.30	0.56	0.28	0.16	0.14	0.12	0.06	0.07	0.14
					**SVM-PBF classifier**					
**SVM-MRMR**	95.56	95.17	94.61	94.52	93.78	93.78	93.78	93.84	93.78	93.78
	0.44	0.36	0.41	0.74	0.56	0.54	0.67	0.85	0.56	0.46
**MRMR**	93.11	93.17	93.44	91.78	91.78	91.78	91.78	91.84	91.63	91.07
	0.44	0.41	0.42	0.46	0.42	0.39	0.23	0.47	0.57	0.59
**SVM-RFE**	94.56	94.72	93.61	92.04	92.04	92.78	92.78	93.30	93.32	93.69
	0.44	0.28	0.56	0.74	0.74	0.23	0.24	0.18	0.16	0.23
**Inf. Gain**	93.00	93.00	92.17	90.78	90.78	90.78	90.78	90.66	90.73	90.78
	0.46	0.59	0.41	0.45	0.74	0.56	0.23	0.32	0.49	0.30
**Wilcoxon test**	91.67	92.72	91.33	90.78	90.78	90.78	90.33	89.20	89.25	89.06
	0.54	0.36	0.42	0.45	0.57	0.32	0.48	0.43	0.49	0.44
**t-test**	92.56	92.44	91.89	90.78	89.30	90.78	90.48	90.37	90.23	89.77
	0.61	0.45	0.41	0.74	0.48	0.55	0.89	0.87	0.71	0.57
**PCR**	91.22	92.44	91.61	90.78	89.30	90.78	90.33	90.78	90.78	90.78
	0.54	0.28	0.56	0.56	0.66	0.48	0.66	0.81	0.47	0.57

**F-Score**	91.22	92.44	92.17	90.78	90.78	90.78	90.78	90.78	90.78	90.68
	0.54	0.28	0.41	0.53	0.58	0.52	0.31	0.51	0.58	0.51
**Gain Ratio**	93.00	93.00	93.00	91.52	90.78	90.04	89.30	90.08	90.09	90.37
	0.77	0.74	0.74	0.74	0.74	0.74	0.26	0.17	0.14	0.19
**BSM**	98.56	98.72	98.61	97.04	97.78	97.04	97.78	97.84	97.73	97.07
	0.32	0.30	0.56	0.28	0.16	0.14	0.12	0.06	0.07	0.14
**SVM-RBF classifier**										
**SVM-MRMR**	95.26	94.59	94.31	93.48	93.48	93.48	93.48	93.54	93.48	93.48
	0.44	0.42	0.41	0.66	0.45	0.52	0.40	0.37	0.26	0.20
**MRMR**	93.56	92.89	92.89	91.78	91.78	91.78	91.78	91.84	91.68	91.78
	0.44	0.42	0.66	0.56	0.47	0.50	0.57	0.58	0.65	0.26
**SVM-RFE**	93.11	94.39	93.28	94.30	94.30	93.56	94.48	94.72	94.63	94.68
	0.44	0.72	0.28	0.74	0.74	0.00	0.20	0.06	0.11	0.18
**Inf. Gain**	90.78	90.50	89.39	90.04	89.30	90.78	89.89	89.37	89.74	90.07
	0.00	0.50	0.58	0.74	0.74	0.00	0.29	0.23	0.18	0.23
**Wilcoxon test**	91.22	92.17	90.22	90.78	90.04	90.78	89.30	90.02	90.19	90.37
	0.44	0.41	0.56	0.00	0.74	0.00	0.35	0.17	0.15	0.19
**t-test**	92.11	92.72	91.89	90.78	90.78	90.78	89.89	89.55	89.40	89.16
	0.54	0.28	0.42	0.00	0.00	0.00	0.29	0.18	0.16	0.26
**PCR**	91.22	92.44	91.06	90.78	90.78	90.78	90.63	90.78	90.78	90.78
	0.44	0.36	0.66	0.00	0.00	0.00	0.15	0.00	0.00	0.00
**F-Score**	91.22	92.44	91.89	90.78	90.78	90.04	90.48	90.72	90.73	90.78
	0.44	0.36	0.59	0.00	0.00	0.74	0.20	0.06	0.05	0.00
**Gain Ratio**	91.22	91.89	92.17	89.30	90.04	90.78	89.44	89.84	90.04	89.87
	0.44	0.42	0.41	0.74	0.74	0.00	0.29	0.18	0.16	0.24
**BSM**	97.11	97.17	94.39	93.56	93.56	93.56	94.44	95.25	95.28	95.37
	0.54	0.58	0.41	0.00	0.00	0.00	0.29	0.16	0.14	0.19
**SVM-SBF classifier**										
**SVM-MRMR**	35.78	39.89	37.94	40.81	40.07	40.07	40.22	38.46	39.58	41.76
	2.29	1.77	1.62	1.48	1.48	1.48	1.08	1.02	0.88	1.25
**MRMR**	31.56	34.17	34.17	30.37	32.59	34.07	32.44	34.15	33.78	33.94
	0.83	2.29	1.39	1.28	1.28	4.12	1.29	0.76	0.61	0.96
**SVM**	35.11	38.33	40.00	42.22	42.22	38.52	39.56	43.45	39.95	38.59
	2.57	1.45	2.59	1.48	3.23	0.74	1.48	0.63	0.88	0.95
**Inf. Gain**	31.56	31.11	31.39	28.89	34.81	34.81	35.41	34.33	33.88	33.43
	2.37	1.51	1.75	1.28	3.92	2.96	1.00	0.87	0.87	1.51
**Wilcoxon test**	32.44	30.83	35.00	32.59	34.07	31.85	35.70	32.28	33.09	33.64
	1.51	1.29	1.38	3.92	5.79	0.74	1.66	0.91	0.99	1.02
**t-test**	30.67	33.33	36.67	35.56	35.56	32.59	34.22	33.86	33.78	33.13
	1.30	1.78	1.88	4.63	4.44	1.96	1.72	0.92	0.88	0.91
**Lin. Correlation**	30.67	30.28	35.56	31.85	32.59	33.33	36.44	34.15	34.47	34.34
	1.78	1.92	1.57	4.12	0.74	2.57	1.88	0.98	0.84	1.27
**F-Score**	33.78	35.00	35.00	28.15	34.81	37.04	33.93	33.16	35.46	35.05
	5.04	2.29	2.13	2.96	2.67	1.96	1.09	0.89	0.81	1.15
**Gain Ratio**	37.78	34.72	34.44	37.04	32.59	36.30	32.89	33.80	32.64	34.14
	2.53	1.78	1.39	1.48	3.23	3.23	1.43	0.78	0.68	0.98
**BSM**	41.83	39.17	40.28	40.83	40.09	39.35	40.09	41.83	41.62	40.43
	3.25	2.44	2.25	1.28	1.96	3.23	1.40	0.73	0.75	1.12

For each method, the first row represents the value of mean CA and second row represents the value of standard error in CA

Table S12. Mean and standard error in classification accuracy computed through varying sliding windows technique for cold stress in rice.

	Gene sets									
**Methods**	**10**	**20**	**50**	**100**	**150**	**200**	**500**	**1000**	**1200**	**1500**
					**SVM-LBF classifier**					
**SVM-MRMR**	95.29	96.00	95.55	94.81	96.00	96.00	94.10	94.59	93.78	91.94
	0.71	0.86	0.81	1.19	1.05	1.15	0.59	0.32	0.46	0.82
**MRMR**	95.00	94.55	93.66	93.81	95.00	95.00	91.90	90.96	91.98	89.32
	0.00	0.45	0.65	1.19	1.48	1.76	1.09	0.57	0.45	1.34
**SVM-RFE**	93.57	96.88	97.77	95.00	94.66	95.00	95.00	95.00	95.00	94.51
	1.34	1.05	0.94	0.72	0.96	0.90	0.93	0.63	0.72	0.49
**Inf. Gain**	93.00	93.00	93.00	93.00	91.81	93.00	87.05	85.95	85.54	86.67
	1.90	1.48	1.18	1.76	1.62	1.66	1.66	0.71	0.62	0.66
**Wilcoxon test**	93.00	91.66	90.77	85.86	88.10	86.90	85.24	83.46	82.06	80.84
	0.00	0.65	0.94	2.06	2.38	1.19	0.77	0.76	0.76	0.98
**t-test**	91.57	91.66	89.43	87.05	85.29	82.90	82.90	79.08	78.38	75.22
	0.87	0.65	1.17	3.15	2.06	1.19	0.57	0.61	0.53	1.19
**PCR**	92.29	92.11	91.66	90.62	91.81	91.81	90.14	87.83	86.89	86.67
	0.71	0.58	0.94	2.38	1.19	1.19	0.71	0.73	0.75	0.91
**F-Score**	91.57	92.11	91.66	91.81	91.81	90.62	89.90	87.92	87.84	86.34
	0.87	0.58	0.65	1.19	1.19	1.19	0.91	0.55	0.62	0.79
**Gain Ratio**	93.00	93.00	92.55	78.71	78.71	84.67	88.00	86.23	85.86	87.64
	2.71	2.49	2.45	4.12	2.06	2.38	1.25	0.61	0.72	0.99
**BSM**	95.00	98.21	97.65	97.65	97.65	97.65	97.65	97.56	96.54	96.33
	1.67	0.95	0.45	0.40	0.32	0.35	0.30	0.24	0.21	0.20
					**SVM-PBF classifier**					
**SVM-MRMR**	95.29	96.00	95.11	94.81	96.00	94.81	93.38	94.03	93.86	92.27
	0.71	1.76	0.58	1.48	1.19	1.19	0.74	0.35	0.42	0.98
**MRMR**	94.29	95.00	93.66	92.62	93.81	95.00	92.14	90.77	89.92	87.53
	0.71	1.76	0.65	1.19	1.19	1.59	0.87	0.63	0.69	1.43
**SVM-RFE**	94.29	93.98	95.32	93.00	93.00	93.62	92.00	94.91	94.92	91.00
	0.87	1.42	1.75	2.38	2.38	2.38	0.98	0.94	0.79	0.74
**Inf. Gain**	93.00	93.00	93.00	93.00	93.00	93.00	87.29	86.05	84.43	84.72
	1.86	1.86	1.86	1.86	1.86	1.86	1.43	0.76	0.77	1.01
**Wilcoxon test**	92.29	90.77	90.77	83.48	82.29	79.90	77.29	77.02	76.02	74.66
	0.71	0.94	0.65	4.29	2.06	1.19	0.68	0.60	0.66	0.98
**t-test**	89.43	89.88	89.43	87.05	79.90	79.90	81.81	78.15	77.52	75.14
	0.67	0.45	0.67	3.15	1.19	1.19	0.84	0.56	0.51	0.91
**PCR**	90.14	91.21	90.77	93.00	93.00	91.81	89.67	87.55	86.89	86.02
	0.71	0.67	0.94	1.48	1.62	1.19	0.74	0.72	0.65	0.68
**F-Score**	90.14	91.21	89.88	93.00	93.00	89.43	89.19	87.55	87.37	85.53
	0.71	0.67	1.05	1.36	1.14	3.57	0.55	0.70	0.58	1.12
**Gain Ratio**	93.00	92.55	91.21	81.10	84.67	83.48	86.81	85.95	85.54	86.83
	1.98	0.95	0.99	4.29	1.19	1.19	0.81	0.77	0.76	0.75
**BSM**	97.07	97.61	98.05	97.31	97.00	95.81	96.29	96.81	96.21	96.68
	1.29	0.58	0.45	1.19	0.75	0.90	0.38	0.13	0.30	0.22
					**SVM-RBF classifier**					
**SVM-MRMR**	96.00	96.00	94.21	94.81	93.62	96.00	94.33	94.31	93.70	92.59
	1.90	1.48	0.95	1.19	1.19	0.78	0.59	0.32	0.43	0.95

**MRMR**	95.00	94.55	94.11	92.62	92.62	93.81	91.19	91.33	89.37	89.48
	0.96	0.45	0.58	1.19	1.19	1.19	1.18	0.55	0.84	1.14
**SVM-RFE**	93.57	94.64	95.54	95.24	96.43	96.43	96.43	95.77	94.84	95.13
	0.71	1.17	1.12	1.19	1.48	1.62	0.35	0.33	0.43	0.76
**Inf. Gain**	93.00	92.11	91.21	87.05	87.05	89.43	84.43	77.40	76.57	76.28
	0.85	0.58	0.67	1.19	1.76	1.62	1.55	1.47	1.18	2.17
**Wilcoxon test**	93.00	91.66	89.88	88.24	83.48	82.29	79.67	77.30	75.30	75.31
	0.94	0.94	1.05	1.19	1.19	1.48	0.89	0.76	0.86	1.38
**t-test**	90.86	89.88	88.09	84.67	81.10	79.90	79.43	79.75	78.40	75.14
	0.87	0.45	0.94	1.19	1.19	1.19	0.94	0.40	0.59	1.29
**PCR**	91.57	90.77	91.21	91.81	93.00	91.81	89.67	87.36	87.37	86.67
	0.87	0.65	0.95	1.19	1.48	1.19	0.95	0.58	0.50	0.88
**F-Score**	90.86	90.77	91.66	91.81	93.00	91.81	89.90	87.45	87.05	85.53
	1.43	0.65	0.65	1.19	1.76	1.19	0.48	0.60	0.53	0.74
**Gain Ratio**	88.00	88.98	83.18	70.38	75.14	78.71	82.29	74.58	77.37	79.85
	0.87	1.05	1.31	2.38	2.81	2.06	1.39	1.50	1.18	1.73
**BSM**	93.57	96.88	97.77	96.43	96.43	98.81	97.38	95.96	95.32	95.45
	1.34	1.05	0.65	1.06	0.62	1.19	0.74	0.47	0.42	0.48
**SVM-SBF classifier**										
**SVM-MRMR**	35.00	44.64	43.30	45.24	33.33	40.48	39.76	38.06	38.17	41.07
	4.43	2.94	2.18	1.19	5.19	8.33	1.90	1.21	1.45	1.46
**MRMR**	35.00	43.30	41.07	46.43	46.43	39.29	42.38	41.54	40.56	43.34
	2.08	2.65	3.51	7.14	3.57	3.57	1.77	1.26	0.88	1.70
**SVM-RFE**	45.00	36.61	40.18	50.00	39.29	44.05	40.71	39.19	39.84	37.18
	4.01	3.22	3.15	3.57	3.57	5.19	1.77	1.47	1.06	2.20
**Inf. Gain**	34.29	45.98	41.96	39.29	40.48	33.33	38.81	39.29	40.24	37.82
	2.14	2.38	2.50	3.57	4.29	6.30	2.00	1.35	1.43	1.93
**Wilcoxon test**	45.71	40.18	38.39	40.48	42.86	36.90	39.76	40.13	39.44	39.45
	2.08	3.36	3.00	4.29	0.00	3.15	2.00	1.50	1.38	1.67
**t-test**	40.71	46.88	42.86	36.90	47.62	42.86	40.00	40.32	37.14	40.10
	4.16	1.96	3.17	5.19	3.15	6.19	1.40	1.59	1.34	1.77
**PCR**	40.00	39.29	38.39	39.29	38.10	32.14	36.19	41.26	41.59	38.80
	2.62	2.13	3.15	9.45	3.15	5.46	2.46	1.35	1.30	1.55
**F-Score**	40.71	38.84	37.50	40.48	39.29	46.43	36.90	39.94	40.08	42.53
	4.01	3.47	1.79	4.29	7.14	3.57	1.58	1.56	1.28	1.39
**Gain Ratio**	41.43	40.63	36.16	46.43	40.48	42.86	38.57	38.63	38.81	37.34
	3.68	2.52	2.82	3.57	4.29	6.19	1.78	1.39	1.33	1.50
**BSM**	40.86	44.52	39.61	42.29	39.90	42.29	43.24	44.07	43.24	41.47
	3.50	2.43	3.00	3.57	3.15	3.57	2.27	1.43	1.11	1.41

For each method, the first row represents the value of mean CA and second row represents the value of standard error in CA

Table S13. Mean and standard error in classification accuracy computed through varying sliding windows technique for drought stress in rice.

	**Gene sets**									
**Methods**	**10**	**20**	**50**	**100**	**150**	**200**	**500**	**1000**	**1200**	**1500**
**SVM-LBF classifier**										
**SVM-MRMR**	95.57	95.93	95.57	95.57	95.57	95.57	95.57	95.57	95.51	95.57
	0.88	0.71	0.78	0.57	0.96	0.89	0.84	0.88	0.44	0.44
**MRMR**	94.57	94.93	94.75	94.57	93.62	94.57	94.57	94.57	94.57	94.57

	1.06	0.95	1.00	0.91	0.95	0.98	0.91	0.92	0.95	0.95
**SVM-RFE**	96.00	96.00	95.46	95.52	95.52	95.52	94.76	94.61	94.60	94.64
	0.61	0.68	0.68	0.76	0.48	0.49	0.79	0.76	0.46	0.65
**Inf. Gain**	93.00	92.64	91.75	91.57	91.57	90.62	91.57	91.53	91.57	91.44
	0.98	0.96	0.86	0.81	0.88	0.81	0.95	0.52	0.95	0.90
**Wilcoxon test**	91.86	92.64	92.82	91.57	90.62	91.57	91.57	91.23	91.41	91.51
	0.86	0.80	0.79	0.95	0.92	0.90	0.81	0.70	0.68	0.65
**t-test**	92.43	92.29	91.93	91.57	90.62	91.57	91.57	91.46	91.48	91.57
	0.35	0.27	0.23	0.95	0.95	0.95	0.95	0.06	0.72	0.75
**PCR**	92.43	92.29	92.11	91.57	91.10	90.14	91.57	91.42	91.54	91.51
	0.99	0.98	0.81	0.87	0.95	0.82	0.88	0.72	0.75	0.65
**F-Score**	92.14	92.29	91.93	91.57	91.10	91.57	91.57	91.35	91.54	91.57
	0.99	1.00	0.88	0.49	0.48	0.62	0.70	0.36	0.75	0.60
**Gain Ratio**	92.14	91.93	91.75	91.57	91.10	91.57	91.57	91.53	91.48	91.57
	0.99	0.88	0.79	0.79	0.76	0.90	0.62	0.76	0.72	0.75
**BSM**	98.54	98.54	98.54	97.11	97.11	96.64	96.73	96.78	96.92	96.98
	0.49	0.48	0.42	0.40	0.38	0.19	0.17	0.13	0.11	0.13
**SVM-PBF classifier**										
**SVM-MRMR**	95.57	95.75	95.75	95.57	94.62	95.57	95.38	95.53	95.48	95.57
	0.95	0.92	0.81	0.52	0.95	0.81	0.76	0.76	0.54	0.55
**MRMR**	94.57	94.75	94.39	94.57	94.57	93.62	94.38	94.50	94.54	94.51
	0.79	0.71	0.98	0.95	0.92	0.91	0.90	0.75	0.75	0.65
**SVM-RFE**	96.71	97.00	96.64	97.00	97.00	97.00	96.33	95.87	95.73	95.64
	0.86	0.86	0.34	0.96	0.82	0.57	0.76	0.96	0.68	0.65
**Inf. Gain**	93.00	92.46	91.57	91.57	91.57	91.57	91.57	91.35	91.41	91.18
	0.81	0.85	0.87	0.78	0.86	0.79	0.64	0.78	0.68	0.66
**Wilcoxon test**	91.86	92.64	92.82	91.57	91.57	91.57	91.57	91.16	91.29	91.51
	0.86	0.80	0.79	0.77	0.77	0.79	0.79	0.65	0.97	0.65
**t-test**	91.86	91.57	91.75	91.57	91.57	91.57	91.57	91.38	91.51	91.57
	0.86	0.89	0.79	0.79	0.68	0.68	0.43	0.79	0.83	0.83
**PCR**	91.86	91.57	91.57	90.62	90.62	91.57	91.29	91.35	91.41	91.51
	0.86	0.89	0.71	0.95	0.98	0.91	0.85	0.86	0.77	0.65
**F-Score**	91.86	91.57	91.57	91.57	91.57	91.57	91.57	91.27	91.35	91.44
	0.86	0.89	0.81	0.71	0.67	0.57	0.59	0.96	0.74	0.59
**Gain Ratio**	91.86	91.93	91.75	91.57	91.57	91.57	91.48	91.46	91.41	91.38
	0.86	0.34	0.79	0.57	0.79	0.77	0.95	0.82	0.82	0.70
**BSM**	98.17	98.17	98.17	96.74	96.74	96.74	96.36	96.55	96.67	96.74
	0.17	0.12	0.14	0.16	0.17	0.17	0.18	0.08	0.04	0.03
**SVM-RBF classifier**										
**SVM-MRMR**	95.57	95.93	95.75	95.57	95.57	95.57	95.48	95.57	95.57	95.57
	0.80	0.85	0.79	0.77	0.76	0.78	0.95	0.98	0.96	0.86
**MRMR**	94.57	94.93	94.75	94.57	94.57	94.57	94.57	94.57	94.54	94.57
	0.79	0.79	0.77	0.57	0.86	0.71	0.79	0.79	0.75	0.75
**SVM-RFE**	96.57	95.86	96.93	96.57	96.57	96.57	96.57	96.57	96.57	96.57
	0.82	0.88	0.71	0.77	0.67	0.66	0.66	0.76	0.57	0.71
**Inf. Gain**	91.57	91.57	91.39	91.57	91.57	91.57	91.57	91.42	91.57	91.57
	0.86	0.79	0.71	0.57	0.57	0.43	0.88	0.79	0.91	0.82
**Wilcoxon test**	91.86	92.64	92.82	91.57	91.57	91.57	91.57	91.57	91.57	91.57
	0.86	0.80	0.79	0.77	0.57	0.71	0.79	0.68	0.86	0.86
**t-test**	92.14	91.93	91.75	91.57	91.57	91.57	91.57	91.53	91.54	91.57

	0.99	0.80	0.79	0.79	0.74	0.88	0.79	0.84	0.73	0.67
**PCR**	92.14	92.11	91.57	91.57	91.57	90.62	91.57	91.57	91.57	91.57
	0.35	0.26	0.26	0.26	0.26	0.48	0.48	0.48	0.48	0.48
**F-Score**	92.14	92.11	91.57	91.57	91.57	91.57	91.38	91.57	91.57	91.51
	0.35	0.26	0.26	0.26	0.26	0.26	0.13	0.13	0.13	0.65
**Gain Ratio**	91.86	91.93	91.39	91.57	91.57	91.57	91.57	91.57	91.48	91.57
	0.86	0.80	0.79	0.86	0.71	0.86	0.86	0.79	0.95	0.90
**BSM**	98.77	98.77	98.59	97.34	97.34	97.34	97.34	97.34	97.34	97.34
	0.18	0.16	0.14	0.11	0.13	0.17	0.11	0.16	0.18	0.11
					**SVM-SBF classifier**					
**SVM-MRMR**	34.00	34.82	34.82	36.67	32.86	34.76	34.86	34.77	34.86	34.61
	1.46	1.26	0.85	2.65	1.65	0.48	0.99	0.62	0.56	0.56
**MRMR**	36.00	35.71	36.43	34.76	35.71	35.24	34.57	35.68	34.76	34.94
	1.53	1.24	0.54	3.33	0.82	1.26	0.80	0.45	0.50	0.65
**SVM-RFE**	34.29	35.36	35.18	32.86	38.10	33.81	35.71	34.74	34.92	35.26
	1.92	1.07	0.85	2.18	0.95	2.08	0.75	0.55	0.46	0.64
**Inf. Gain**	36.86	34.11	35.89	32.86	33.81	37.62	34.10	33.87	35.90	35.84
	1.31	1.39	0.91	1.43	1.72	2.38	0.75	0.56	0.55	0.61
**Wilcoxon test**	34.00	36.07	36.07	37.14	35.24	34.76	34.95	35.38	36.16	34.87
	1.94	0.59	1.07	0.82	0.48	1.26	0.98	0.60	0.46	0.70
**t-test**	35.14	32.14	36.79	32.86	36.67	32.86	35.33	34.96	35.02	34.81
	1.16	1.66	0.84	0.82	1.72	2.18	0.94	0.53	0.50	0.68
**PCR**	35.43	34.64	35.18	37.14	34.76	33.33	34.57	35.41	35.71	35.39
	0.95	1.00	0.60	2.86	1.26	0.48	0.89	0.67	0.36	0.71
**F-Score**	34.86	31.43	35.36	31.90	38.10	33.33	33.90	35.23	36.22	36.88
	2.00	1.08	1.14	2.38	1.72	0.48	0.95	0.49	0.45	0.68
**Gain Ratio**	34.00	34.82	35.00	31.90	36.19	35.24	34.29	35.23	35.62	35.52
	0.83	1.14	1.24	1.72	3.12	1.90	0.95	0.57	0.48	1.00
**BSM**	39.86	41.79	40.89	42.62	38.33	41.67	39.67	40.00	40.75	40.39
	0.97	0.89	1.13	1.26	0.95	1.26	0.84	0.46	0.47	0.80

For each method, the first row represents the value of mean CA and second row represents the value of standard error in CA

Table S14. Mean and standard error in classification accuracy computed through varying sliding windows technique for bacterial stress in rice.

					**Gene sets**					
**Methods**	**10**	**20**	**50**	**100**	**150**	**200**	**500**	**1000**	**1200**	**1500**
					**SVM-LBF classifier**					
**SVM-MRMR**	96.54	96.54	96.54	96.54	96.54	96.54	96.45	96.44	96.00	95.74
	1.90	1.60	1.13	1.19	1.09	1.11	0.90	0.60	0.13	0.19
**MRMR**	95.77	95.77	95.77	95.77	95.77	95.77	95.68	95.55	95.47	94.91
	2.19	2.08	2.11	1.72	1.21	1.09	0.89	0.81	0.11	0.21
**SVM-RFE**	96.33	96.33	96.54	96.54	96.54	95.64	96.54	96.54	96.51	96.24
	1.89	1.46	1.30	1.03	1.00	0.90	0.57	0.46	0.03	0.20
**Inf. Gain**	92.77	92.77	92.77	92.77	92.77	92.32	91.96	92.05	91.90	90.74
	2.45	2.26	2.19	1.50	1.45	0.45	0.26	0.19	0.17	0.37
**Wilcoxon test**	92.88	92.88	92.88	92.88	92.88	92.88	92.61	92.77	92.55	92.26
	2.14	1.99	1.75	1.32	1.14	0.99	0.14	0.06	0.10	0.32
**t-test**	92.88	92.88	92.88	92.88	92.88	92.88	92.61	92.31	92.25	91.03
	2.44	1.97	1.88	1.72	1.66	0.86	0.14	0.17	0.18	0.42

**PCR**	92.77	92.77	92.77	92.77	92.77	92.32	92.59	92.45	92.25	91.66
	2.66	1.54	1.16	1.20	1.36	0.77	0.12	0.11	0.14	0.35
**F-Score**	92.88	92.88	92.88	92.88	92.88	92.88	92.88	92.31	92.01	90.97
	2.57	1.39	1.13	1.09	1.03	0.99	0.85	0.15	0.17	0.34
**Gain Ratio**	92.17	92.17	92.17	92.17	92.17	92.17	92.08	91.99	91.38	89.59
	2.12	2.59	1.59	1.32	1.12	1.07	0.90	0.22	0.25	0.44
**BSM**	97.88	97.88	96.88	96.88	96.88	96.43	96.61	96.52	96.13	95.53
	1.11	1.85	1.18	1.11	0.67	0.45	0.20	0.11	0.11	0.27
**SVM-PBF classifier**										
**SVM-MRMR**	96.11	96.11	96.11	96.11	96.11	96.11	96.02	95.93	95.75	94.75
	1.90	1.75	1.12	1.29	1.02	1.01	0.09	0.08	0.12	0.29
**MRMR**	94.55	94.55	94.55	94.55	94.10	94.55	94.55	94.37	94.22	93.20
	2.45	2.05	2.11	1.75	1.22	0.98	0.45	0.08	0.10	0.28
**SVM-RFE**	96.04	96.04	96.04	95.59	96.04	96.04	96.04	96.00	96.04	95.42
	1.25	1.05	1.15	0.95	0.84	1.04	0.94	0.74	0.04	0.21
**Inf. Gain**	91.88	91.88	91.88	91.88	91.88	91.55	91.01	91.29	90.83	89.67
	2.45	1.95	1.50	1.45	0.95	0.75	0.41	0.17	0.22	0.49
**Wilcoxon test**	91.77	91.77	91.77	91.77	91.77	91.77	91.41	91.59	91.56	91.15
	2.21	1.92	1.82	1.71	1.55	1.33	0.81	0.51	0.09	0.25
**t-test**	91.43	91.43	91.43	91.43	91.43	91.43	91.16	91.01	90.77	90.14
	2.14	1.81	1.14	1.66	0.94	0.74	0.44	0.15	0.19	0.27
**PCR**	91.11	91.11	91.11	91.11	91.11	91.11	90.66	91.11	90.87	90.00
	0.28	0.28	0.28	0.28	0.28	0.28	0.28	0.19	0.09	0.35
**F-Score**	91.43	91.43	91.43	91.43	91.43	91.43	91.43	90.79	90.62	88.98
	1.73	1.73	1.73	1.73	1.73	1.73	1.73	0.14	0.18	0.52
**Gain Ratio**	92.10	92.10	92.10	92.10	92.10	92.10	92.10	91.75	91.41	88.73
	1.76	1.76	1.76	1.76	1.76	1.76	1.76	0.13	0.14	0.56
**BSM**	97.65	97.65	97.65	97.65	97.20	96.75	97.56	97.26	96.81	96.24
	1.42	1.12	1.12	1.12	0.45	0.45	0.09	0.10	0.14	0.27
**SVM-RBF classifier**										
**SVM-MRMR**	96.54	96.54	96.54	96.54	96.54	96.09	95.55	94.69	93.63	90.03
	1.65	1.60	0.96	0.90	0.84	0.45	0.36	0.24	0.23	0.35
**MRMR**	95.77	95.77	95.77	95.77	95.77	95.77	94.50	94.09	93.00	89.19
	1.52	0.86	0.65	0.52	0.86	0.71	0.52	0.20	0.30	0.39
**SVM-RFE**	96.38	95.47	95.13	93.95	93.50	92.60	94.85	94.66	94.76	94.01
	1.27	0.70	0.83	2.06	0.90	0.80	0.22	0.22	0.16	0.29
**Inf. Gain**	92.55	92.38	92.55	92.10	91.20	88.94	89.12	87.57	85.82	81.98
	2.17	1.89	1.69	1.45	0.75	1.62	0.56	0.48	0.39	0.71
**Wilcoxon test**	92.33	92.33	92.33	91.88	90.97	90.52	90.16	90.41	89.62	87.23
	2.45	1.75	1.50	1.11	0.95	0.85	0.52	0.30	0.31	0.49
**t-test**	92.55	92.55	92.55	92.55	91.20	90.75	89.57	89.24	87.89	83.58
	1.59	1.93	1.72	1.55	1.12	0.45	0.59	0.37	0.46	0.75
**PCR**	92.46	92.46	92.46	92.01	91.11	91.11	90.84	90.15	89.84	87.24
	1.78	1.20	0.88	0.45	0.78	0.78	0.38	0.39	0.28	0.48
**F-Score**	93.87	93.87	93.87	93.87	92.96	90.26	91.25	88.42	86.63	83.05
	1.24	1.37	1.29	1.12	0.90	0.90	0.28	0.44	0.55	0.62
**Gain Ratio**	93.34	93.88	93.88	91.17	92.53	91.17	88.92	87.26	85.65	81.78
	1.96	1.60	1.35	1.94	1.35	0.78	0.41	0.41	0.33	0.64
**BSM**	97.16	97.26	97.26	95.18	94.73	94.95	94.77	94.26	93.54	90.79
	0.27	0.17	0.17	0.90	0.78	0.78	0.39	0.23	0.27	0.66

					SVM-SBF classifier					
SVM-MRMR	36.18	36.99	35.13	36.54	31.13	37.44	36.18	36.95	36.72	37.91
	1.18	1.25	1.69	2.51	1.19	0.45	0.68	0.56	0.60	0.76
MRMR	34.59	34.80	34.29	35.14	36.94	36.49	36.85	36.02	36.01	36.00
	1.01	0.91	0.99	2.81	0.45	1.35	0.83	0.48	0.48	0.91
SVM-RFE	35.95	34.63	36.99	35.14	39.64	35.59	35.05	36.88	35.74	36.86
	0.92	0.67	0.88	1.35	0.45	2.51	1.15	0.48	0.62	0.78
Inf. Gain	33.78	34.80	37.84	34.23	36.94	36.04	35.86	34.89	37.33	35.75
	1.42	1.84	1.05	2.74	1.19	1.96	0.81	0.55	0.41	0.85
Wilcoxon test	33.78	35.14	36.99	36.49	38.29	41.44	36.31	34.99	36.58	35.81
	0.60	1.25	1.02	1.56	2.38	0.45	0.76	0.59	0.47	0.77
t-test	36.76	36.66	35.30	36.94	37.39	36.49	36.04	35.95	35.65	36.98
	1.16	0.74	1.04	0.45	1.96	1.35	0.87	0.51	0.44	0.66
PCR	35.68	33.78	35.81	35.59	33.78	36.94	35.05	35.35	36.13	37.29
	1.58	1.40	1.53	1.62	0.78	3.52	0.90	0.50	0.47	0.63
F-Score	37.03	34.80	35.47	39.64	34.23	36.04	36.13	35.95	35.68	35.63
	1.46	1.19	1.71	0.45	0.45	1.62	1.02	0.71	0.46	0.77
Gain Ratio	35.41	35.98	36.49	36.49	34.23	34.23	35.14	36.06	35.59	37.04
	1.16	1.08	0.88	1.35	1.62	1.96	0.96	0.55	0.55	0.77
BSM	38.76	37.64	40.85	35.33	37.14	36.68	37.95	37.95	38.07	40.21
	0.70	1.15	0.75	1.76	1.15	2.54	0.78	0.40	0.35	0.51

For each method, the first row represents the value of mean CA and second row represents the value of standard error in CA

Table S15. Mean and standard error in classification accuracy computed through varying sliding windows technique for fungal stress in rice.

					Gene sets					
**Methods**	**10**	**20**	**50**	**100**	**150**	**200**	**500**	**1000**	**1200**	**1500**
					**SVM-LBF classifier**					
**SVM-MRMR**	96.21	96.21	96.21	96.21	96.21	96.21	96.21	95.70	95.87	95.69
	1.26	0.83	0.83	0.83	0.83	0.83	0.83	0.26	0.21	0.38
**MRMR**	95.21	95.21	95.21	95.21	95.21	95.21	95.21	94.70	94.53	94.69
	1.45	1.45	1.45	1.45	1.45	1.45	1.45	0.33	0.28	0.38
**SVM-RFE**	96.01	96.01	96.01	96.01	96.01	96.01	96.01	96.01	96.01	95.01
	1.28	1.28	1.28	1.28	1.28	1.28	1.28	1.28	1.28	0.83
**Inf. Gain**	92.43	92.43	92.43	92.43	92.43	92.43	92.18	91.22	90.89	89.98
	1.64	1.64	1.64	1.64	1.64	1.64	0.26	0.29	0.31	0.65
**Wilcoxon test**	92.65	92.65	92.65	92.65	92.65	92.65	92.65	92.25	91.71	91.96
	1.85	1.85	1.85	1.85	1.85	1.85	1.85	0.24	0.33	0.54
**t-test**	93.10	93.10	93.10	93.10	93.10	93.10	92.59	91.28	91.31	90.13
	1.94	1.94	1.94	1.94	1.94	1.94	0.35	0.40	0.31	0.67
**PCR**	93.44	93.44	93.44	93.44	93.44	93.44	92.67	92.83	93.18	93.09
	1.71	1.71	1.71	1.71	1.71	1.71	0.41	0.27	0.14	0.24
**F-Score**	93.63	93.63	93.63	93.63	93.63	93.63	93.63	93.23	93.46	93.28
	1.94	1.94	1.94	1.94	1.94	1.94	1.94	1.19	0.92	0.35
**Gain Ratio**	93.43	93.43	93.43	93.43	93.43	93.43	93.18	93.13	93.26	93.43
	1.83	1.83	1.83	1.83	1.83	1.83	1.26	0.22	0.17	0.21
**BSM**	97.14	97.14	97.14	97.14	97.14	97.14	97.14	97.14	97.14	97.14
	0.83	0.83	0.83	0.83	0.83	0.83	0.83	0.83	0.83	0.83
					**SVM-PBF classifier**					

**SVM-MRMR**	96.04	96.04	95.56	96.04	96.04	96.04	95.79	94.73	94.50	94.64
	1.48	1.48	1.48	0.86	0.86	0.86	0.26	0.33	0.28	0.40
**MRMR**	94.65	94.65	94.17	94.65	94.65	94.65	94.14	93.14	92.86	92.38
	1.81	1.81	1.48	0.81	0.81	0.81	0.35	0.34	0.29	0.70
**SVM-RFE**	96.77	96.77	94.84	96.77	96.77	96.77	96.77	96.77	96.77	96.24
	1.26	1.26	1.89	0.89	0.89	0.89	0.89	0.89	0.89	0.38
**Inf. Gain**	92.65	92.65	92.17	92.65	92.65	92.65	90.60	89.52	88.81	88.28
	1.81	1.81	0.81	0.77	0.77	0.77	0.51	0.32	0.32	0.46
**Wilcoxon test**	92.88	92.88	92.88	92.88	92.88	92.88	91.85	90.75	90.57	90.25
	1.80	1.80	1.80	1.80	1.80	1.80	0.45	0.35	0.31	0.47
**t-test**	92.33	92.33	92.33	92.33	92.33	92.33	91.04	89.59	88.22	88.31
	1.85	1.85	1.85	1.85	1.85	1.85	1.48	0.43	0.43	0.54
**PCR**	92.65	92.65	92.17	92.65	92.65	92.65	91.63	90.83	90.35	89.86
	1.48	1.48	1.77	0.85	0.85	0.85	0.45	0.32	0.35	0.52
**F-Score**	93.28	93.28	93.28	93.28	93.28	93.28	93.28	92.16	92.34	92.58
	0.99	0.99	0.99	0.99	0.99	0.99	0.99	0.29	0.28	0.32
**Gain Ratio**	92.65	92.65	92.65	92.65	91.37	91.37	91.12	91.64	91.54	92.65
	1.82	1.82	1.82	1.82	1.28	1.28	0.50	0.38	0.31	0.46
**BSM**	97.14	96.66	97.14	97.14	97.14	97.14	97.14	96.94	97.14	96.97
	0.81	0.48	0.48	0.48	0.48	0.48	0.48	0.14	0.11	0.17
**SVM-RBF classifier**										
**SVM-MRMR**	96.12	96.12	96.12	96.12	96.12	96.12	96.12	95.51	95.61	95.77
	1.56	1.56	1.56	1.56	1.56	1.56	1.56	0.23	0.20	0.24
**MRMR**	94.65	94.17	94.65	94.65	94.65	94.65	94.65	94.05	94.31	94.48
	1.48	0.48	0.27	0.27	0.27	0.27	0.27	0.27	0.17	0.17
**SVM-RFE**	96.05	96.05	94.13	96.05	93.49	96.05	95.29	96.05	96.05	96.05
	1.26	1.26	1.59	2.56	2.41	2.56	0.77	0.77	0.77	0.77
**Inf. Gain**	92.65	91.69	92.65	92.65	92.65	92.65	92.14	90.43	89.83	89.33
	1.96	0.96	0.35	0.35	0.35	0.35	0.49	0.40	0.39	0.29
**Wilcoxon test**	92.43	92.43	92.43	92.43	92.43	92.43	92.43	92.23	92.09	92.26
	1.91	1.91	1.91	1.91	1.91	1.91	1.91	1.14	0.17	0.17
**t-test**	92.65	92.65	92.65	92.65	92.65	92.65	91.89	90.53	90.69	90.03
	1.41	1.41	1.41	1.41	1.41	1.41	0.81	0.40	0.29	0.47
**PCR**	92.43	92.43	92.43	92.43	92.43	92.43	92.18	92.03	92.43	91.73
	1.64	1.64	1.64	1.64	1.64	1.64	0.83	0.19	0.94	0.32
**F-Score**	93.54	93.54	93.54	93.54	93.54	92.26	93.54	92.43	92.86	93.54
	1.82	1.82	1.82	1.82	1.82	1.28	1.82	0.35	0.25	0.00
**Gain Ratio**	93.43	92.47	93.43	90.87	93.43	93.43	92.66	93.13	93.35	93.26
	1.96	0.96	2.56	1.56	0.73	0.73	0.41	0.22	0.09	0.17
**BSM**	97.02	96.54	95.09	97.02	97.02	97.02	97.02	97.02	97.02	97.02
	0.48	0.48	1.26	0.26	0.26	0.26	0.26	0.26	0.06	0.26
**SVM-SBF classifier**										
**SVM-MRMR**	30.90	25.96	27.40	23.08	24.36	25.64	28.46	25.10	26.84	25.35
	3.08	2.27	1.69	2.22	3.39	1.28	1.44	1.04	0.89	1.18
**MRMR**	30.77	24.52	27.88	28.21	26.92	23.08	25.64	26.01	25.21	28.15
	2.72	1.91	1.89	1.28	4.44	5.88	1.66	1.02	0.89	1.02
**SVM-RFE**	26.92	26.44	23.56	28.21	23.08	24.36	25.90	24.80	25.73	25.52
	2.72	2.35	1.53	1.28	5.88	3.39	1.43	0.88	0.95	1.17
**Inf. Gain**	30.77	25.48	25.00	33.33	25.64	26.92	26.67	24.19	26.07	26.75
	1.22	2.17	1.63	1.28	1.28	0.00	0.95	1.09	0.69	0.93

**Wilcoxon test**	26.92	27.40	26.44	24.36	26.92	28.21	23.33	26.21	26.84	26.40
	1.72	0.87	2.56	2.56	2.22	4.62	1.48	0.88	0.80	0.99
**t-test**	23.08	26.44	24.52	26.92	26.92	23.08	25.13	25.30	26.67	26.40
	2.43	1.35	2.99	2.22	2.22	4.44	1.54	0.73	0.77	1.08
**PCR**	26.15	26.44	24.04	21.79	26.92	29.49	24.87	26.42	26.58	25.70
	2.24	2.23	2.02	1.28	3.85	5.13	1.24	1.01	0.78	1.20
**F-Score**	28.46	28.85	26.44	29.49	26.92	21.79	25.13	26.92	25.47	27.97
	3.96	2.18	2.11	3.39	0.12	3.39	1.50	0.98	0.80	1.11
**Gain Ratio**	23.85	27.88	25.00	21.79	24.36	23.08	26.67	26.21	26.41	24.30
	2.83	2.70	1.26	5.59	6.78	2.22	1.38	0.81	0.80	1.55
**BSM**	30.92	27.08	28.04	30.92	24.51	29.64	30.41	29.51	29.56	27.10
	2.20	1.78	2.02	1.22	1.56	1.28	1.59	0.72	0.92	0.69

For each method, the first row represents the value of mean CA and second row represents the value of standard error in CA

Table S16. Mean and standard error in classification accuracy computed through varying sliding windows technique for insect stress in rice.

	**Gene sets**									
**Methods**	**10**	**20**	**50**	**100**	**150**	**200**	**500**	**1000**	**1200**	**1500**
					**SVM-LBF classifier**					
**SVM-MRMR**	96.54	96.54	96.54	96.54	96.54	95.62	96.54	96.54	96.48	96.54
	1.26	1.26	1.26	1.26	1.26	1.59	1.17	1.06	0.62	0.73
**MRMR**	94.77	94.77	94.42	94.77	94.77	94.77	94.77	94.77	94.77	94.77
	1.17	1.17	1.22	1.35	1.35	1.35	1.35	1.35	1.35	1.35
**SVM-RFE**	95.21	95.70	96.04	97.43	97.43	97.43	97.43	97.43	97.43	97.43
	0.56	1.04	1.52	0.95	0.95	0.95	0.95	0.95	0.95	0.95
**Inf. Gain**	92.65	92.65	92.65	91.73	89.88	89.88	92.28	92.22	92.10	92.15
	1.59	1.59	1.59	0.93	0.89	0.89	0.25	0.17	0.17	0.23
**Wilcoxon test**	92.77	92.77	92.77	91.84	92.77	92.77	92.58	91.45	91.90	91.88
	1.93	1.93	1.93	1.25	1.93	1.93	0.19	0.34	0.19	0.28
**t-test**	92.65	92.65	92.65	92.65	92.65	92.65	92.47	92.14	92.16	92.28
	1.85	1.85	1.85	1.85	1.85	1.85	0.85	0.77	0.60	0.80
**PCR**	92.34	92.34	92.34	90.49	92.34	92.34	92.34	91.03	91.54	91.08
	1.93	1.93	1.93	1.26	0.93	0.93	0.93	0.31	0.19	0.30
**F-Score**	93.65	93.65	93.65	93.65	93.65	93.65	93.28	93.22	93.53	93.28
	1.65	1.65	1.65	1.65	1.65	1.65	0.52	0.46	0.19	0.80
**Gain Ratio**	93.42	93.42	93.42	93.42	93.42	93.42	92.86	93.34	93.29	93.16
	0.30	0.30	0.30	0.30	0.30	0.30	0.30	0.07	0.09	0.17
**BSM**	96.84	96.84	96.50	96.84	96.84	96.84	96.84	96.84	96.84	96.59
	0.72	0.72	0.35	0.72	0.72	0.72	0.72	0.72	0.72	0.17
					**SVM-PBF classifier**					
**SVM-MRMR**	95.99	96.54	96.54	96.54	95.62	95.62	96.54	96.32	96.48	96.54
	1.56	1.46	1.46	1.46	0.93	0.93	1.46	0.12	0.06	0.46
**MRMR**	94.54	95.31	95.65	95.65	95.65	95.65	95.65	95.65	95.59	95.65
	1.68	1.55	1.35	1.35	1.35	1.35	1.35	1.35	0.07	0.66
**SVM-RFE**	94.11	95.92	95.57	96.76	96.76	96.76	96.76	96.76	96.70	95.76
	1.42	1.14	0.94	0.73	0.73	0.73	0.73	0.73	0.06	0.51
**Inf. Gain**	92.65	92.65	92.65	90.80	89.88	89.88	92.10	91.12	91.30	91.64
	1.93	1.93	1.93	1.59	0.96	0.96	0.70	0.71	0.21	0.29
**Wilcoxon test**	92.27	92.48	92.48	90.97	92.83	92.83	92.83	91.80	91.84	91.94

	1.56	1.35	1.35	0.93	1.26	1.26	1.26	0.82	0.20	0.28
**t-test**	92.76	92.76	91.37	90.91	91.84	91.84	91.84	91.37	91.47	91.75
	1.74	1.74	1.24	0.93	0.93	0.93	0.35	0.26	0.21	0.29
**PCR**	92.87	92.87	92.18	91.02	92.87	92.87	92.69	91.85	91.76	91.86
	1.45	1.45	1.21	0.93	0.93	0.93	0.19	0.22	0.27	0.29
**F-Score**	93.09	93.09	93.09	93.09	93.09	93.09	92.72	92.65	92.84	92.71
	1.25	1.25	1.25	1.25	1.25	1.25	0.65	0.17	0.12	0.28
**Gain Ratio**	93.02	93.02	93.02	93.02	93.02	93.02	92.46	92.80	92.83	92.89
	1.30	1.30	1.30	1.30	1.30	1.30	0.70	0.12	0.10	0.13
**BSM**	97.32	97.32	96.63	97.32	97.32	97.32	97.32	97.25	97.20	96.32
	0.85	0.85	0.66	0.56	0.56	0.56	0.56	0.07	0.09	0.19
**SVM-PBF classifier**										
**SVM-MRMR**	96.81	96.81	96.81	96.81	96.81	96.81	96.81	96.74	96.75	96.81
	1.73	1.73	1.73	1.73	1.73	1.73	1.73	0.90	0.06	0.42
**MRMR**	95.54	95.54	95.19	95.54	95.54	95.54	95.54	95.47	95.54	95.54
	1.23	1.23	1.35	1.23	1.23	1.23	1.23	0.73	0.07	0.47
**SVM-RFE**	95.56	96.36	96.71	96.21	96.21	96.21	96.21	96.21	96.00	95.21
	1.41	0.99	0.85	0.52	0.52	0.52	0.52	0.52	0.25	0.66
**Inf. Gain**	92.34	92.34	91.30	90.49	89.56	89.56	91.97	91.39	91.72	91.71
	2.04	2.04	1.67	1.26	0.93	0.93	0.25	0.22	0.17	0.25
**Wilcoxon test**	91.70	91.91	92.26	90.40	91.33	92.26	91.88	91.38	91.39	90.99
	1.46	0.72	0.35	0.93	0.93	0.93	0.25	0.21	0.19	0.30
**t-test**	92.10	92.31	92.31	90.80	91.73	91.73	92.65	91.78	91.79	92.28
	0.56	0.35	0.35	0.93	0.93	0.93	0.93	0.21	0.19	0.21
**PCR**	92.32	92.53	92.18	91.02	91.95	92.88	92.88	92.15	91.89	91.61
	0.56	0.35	0.69	0.93	0.93	0.83	0.83	0.20	0.25	0.30
**F-Score**	92.88	92.88	92.88	92.88	92.88	92.88	92.32	92.44	92.57	92.50
	1.23	1.23	1.23	0.97	0.97	0.97	0.30	0.17	0.13	0.21
**Gain Ratio**	92.65	92.65	92.65	92.65	92.65	92.65	91.91	92.36	92.28	92.40
	0.98	0.98	0.98	0.98	0.98	0.98	0.82	0.14	0.14	0.17
**BSM**	96.76	97.88	97.18	97.88	97.88	97.88	97.88	97.80	97.88	97.00
	0.68	0.80	0.62	0.45	0.45	0.45	0.45	0.07	0.31	0.21
**SVM-SBF classifier**										
**SVM-MRMR**	31.67	30.56	32.64	32.41	34.26	25.00	30.00	31.43	29.75	32.07
	2.22	1.15	1.55	1.85	4.04	4.24	1.39	0.69	0.93	1.06
**MRMR**	28.89	29.51	31.25	31.48	25.00	30.56	30.93	29.82	29.63	29.67
	2.26	1.65	2.21	2.45	0.23	2.78	1.41	0.66	0.77	1.06
**SVM-RFE**	24.44	25.69	28.82	30.56	29.63	29.63	30.93	29.31	31.05	29.17
	3.45	2.86	1.57	0.35	1.85	2.45	1.05	0.68	0.83	1.45
**Inf. Gain**	33.89	29.86	29.17	25.93	31.48	24.07	29.07	30.41	29.63	30.56
	1.04	1.59	1.29	0.93	2.45	3.34	1.35	0.78	0.66	1.11
**Wilcoxon test**	27.78	30.90	31.25	29.63	29.63	35.19	30.93	30.19	30.06	31.31
	1.76	1.92	1.87	1.85	4.63	3.34	1.84	0.68	0.75	0.92
**t-test**	31.11	30.21	29.51	33.33	29.63	32.41	32.78	29.53	30.99	30.43
	2.39	1.78	1.48	1.60	1.85	2.45	1.16	0.99	0.72	1.07
**PCR**	27.22	30.56	27.78	25.00	35.19	26.85	30.37	29.97	30.25	31.19
	2.69	2.73	1.57	1.60	4.04	1.85	1.50	0.84	0.75	1.47
**F-Score**	29.44	31.25	29.17	30.56	27.78	30.56	33.89	29.68	30.68	29.92
	1.11	1.69	1.57	1.60	3.21	2.78	1.25	0.73	0.68	1.00
**Gain Ratio**	28.33	28.47	27.43	26.85	34.26	27.78	30.93	32.09	30.19	31.69

	2.04	2.61	2.75	0.93	3.34	1.60	1.05	0.72	0.74	1.18
**BSM**	35.67	37.68	33.86	32.70	33.63	35.48	30.67	34.26	34.80	32.91
	0.68	1.43	1.55	1.45	1.85	1.34	1.14	0.98	0.75	0.44

For each method, the first row represents the value of mean CA and second row represents the value of standard error in CA

Document S7. Comparative Performance analysis of Gene selection Methods Based on GO Based Dissimilarity Scores for Biotic Stresses in Rice.

The GO based dissimilarity scores for ten gene selection methods under Gene Ontology taxonomies Molecular Function (MF), Biological Process (BP) and Cellular Component (CC) were computed for biotic stresses, i.e. bacterial, fungal and insect, are shown in Tables S17 - S19.

Table S17. Comparative Performance analysis of gene selection methods based on GO_MF based dissimilarity score for biotic stresses in rice.

Methods	MRMR	SVM-RFE	SVM-MRMR	IG	GR	Wilcox	t	PCR	F	BSM
					**Bacterial stress in rice**					
10	1.00	0.75	1.00	0.84	0.90	0.87	0.86	0.86	0.86	**0.79**
20	0.90	0.86	0.90	0.88	0.92	0.89	0.89	0.89	0.89	**0.83**
50	0.91	0.91	0.91	0.90	0.90	0.88	0.89	0.88	0.88	**0.78**
100	0.90	0.91	0.90	0.89	0.88	0.91	0.90	0.90	0.90	**0.80**
150	0.90	0.91	0.90	0.90	0.89	0.90	0.89	0.90	0.90	**0.80**
200	0.90	0.92	0.90	0.90	0.89	0.90	0.89	0.90	0.90	**0.79**
500	0.89	0.90	0.90	0.89	0.90	0.90	0.90	0.90	0.90	**0.82**
					**Fungal stress in rice**					
10	0.84	0.83	0.96	0.89	0.85	0.91	0.84	0.84	0.84	**0.82**
20	0.90	0.86	0.90	0.91	0.89	0.92	0.88	0.88	0.88	**0.83**
50	0.87	0.88	0.91	0.90	0.87	0.89	0.89	0.89	0.89	**0.82**
100	0.88	0.89	0.91	0.88	0.88	0.89	0.89	0.89	0.89	**0.80**
150	0.89	0.89	0.91	0.89	0.85	0.90	0.89	0.89	0.89	**0.80**
200	0.90	0.88	0.91	0.90	0.83	0.90	0.90	0.89	0.89	**0.80**
500	0.89	0.87	0.91	0.88	0.90	0.91	0.87	0.91	0.84	**0.80**
					**Insect stress in rice**					
10	0.91	0.87	0.91	0.92	0.77	0.78	0.78	0.83	0.83	**0.81**
20	0.90	0.93	0.90	0.92	0.92	0.78	0.90	0.87	0.87	**0.81**
50	0.89	0.87	0.89	0.90	0.90	0.89	0.88	0.91	0.91	**0.80**
100	0.90	0.91	0.90	0.90	0.91	0.89	0.88	0.91	0.91	**0.81**
150	0.90	0.90	0.90	0.89	0.92	0.89	0.88	0.90	0.90	**0.81**
200	0.90	0.91	0.90	0.90	0.90	0.90	0.88	0.90	0.90	**0.81**
500	0.91	0.90	0.89	0.89	0.88	0.89	0.89	0.90	0.90	**0.85**

Values marked as bolds represent dissimilarity scores obtained from proposed BSM approach

Table S18. Comparative Performance analysis of gene selection methods based on GO_BP based dissimilarity score for biotic stresses in rice.

	MRMR	SVM-RFE	SVM-MRMR	IG	GR	Wilcox	t	PCR	F	BSM
					**Bacterial stress in rice**					
10	0.97	0.72	0.97	0.87	0.87	0.86	0.93	0.93	0.93	**0.76**
20	0.85	0.91	0.85	0.91	0.89	0.87	0.87	0.87	0.87	**0.79**
50	0.87	0.91	0.87	0.91	0.87	0.86	0.89	0.88	0.88	**0.78**
100	0.88	0.90	0.88	0.91	0.85	0.88	0.87	0.87	0.87	**0.80**
150	0.88	0.89	0.87	0.91	0.86	0.87	0.86	0.86	0.86	**0.80**
200	0.87	0.90	0.87	0.91	0.86	0.86	0.87	0.87	0.87	**0.80**
500	0.86	0.88	0.88	0.88	0.88	0.87	0.87	0.87	0.87	**0.81**
					**Fungal stress in rice**					
10	0.86	0.78	0.88	0.81	0.79	0.83	0.86	0.86	0.86	**0.77**
20	0.87	0.85	0.83	0.87	0.80	0.84	0.88	0.88	0.88	**0.77**

50	0.87	0.84	0.85	0.88	0.84	0.85	0.86	0.86	0.86	**0.76**
100	0.84	0.83	0.86	0.84	0.86	0.87	0.85	0.85	0.85	**0.77**
150	0.84	0.84	0.86	0.87	0.85	0.87	0.85	0.85	0.85	**0.75**
200	0.85	0.84	0.87	0.87	0.84	0.88	0.86	0.85	0.85	**0.75**
500	0.82	0.83	0.88	0.86	0.88	0.88	0.85	0.87	0.83	**0.77**
**Insect stress in rice**										
10	0.90	0.87	0.90	0.92	0.81	0.72	0.94	0.89	0.89	**0.80**
20	0.90	0.87	0.90	0.86	0.88	0.85	0.91	0.88	0.88	**0.80**
50	0.87	0.87	0.87	0.86	0.88	0.86	0.89	0.87	0.87	**0.78**
100	0.87	0.88	0.87	0.87	0.89	0.86	0.85	0.88	0.88	**0.78**
150	0.86	0.87	0.86	0.87	0.88	0.86	0.87	0.87	0.87	**0.79**
200	0.87	0.88	0.87	0.87	0.86	0.86	0.86	0.87	0.87	**0.79**
500	0.88	0.87	0.88	0.85	0.85	0.84	0.86	0.88	0.88	**0.82**

Values marked as bolds represent dissimilarity scores obtained from proposed BSM approach

Table S19. Comparative Performance analysis of gene selection methods based on GO_CC based dissimilarity score for biotic stresses in rice.

	MRMR	SVM-RFE	SVM-MRMR	IG	GR	Wilcox	t	PCR	F	BSM
**Bacterial stress in rice**										
10	1.00	0.74	1.00	0.83	0.94	0.91	0.89	0.89	0.89	**0.83**
20	0.92	0.90	0.92	0.89	0.92	0.91	0.93	0.93	0.93	**0.77**
50	0.90	0.88	0.90	0.90	0.90	0.88	0.90	0.88	0.88	**0.78**
100	0.87	0.89	0.87	0.88	0.89	0.90	0.89	0.90	0.90	**0.77**
150	0.87	0.89	0.88	0.89	0.88	0.90	0.90	0.90	0.90	**0.76**
200	0.89	0.88	0.89	0.88	0.88	0.90	0.90	0.89	0.89	**0.77**
500	0.88	0.87	0.90	0.85	0.89	0.89	0.90	0.89	0.89	**0.82**
**Fungal stress in rice**										
10	0.95	0.74	0.94	0.82	0.90	0.80	0.95	0.95	0.95	**0.73**
20	0.90	0.88	0.88	0.86	0.86	0.90	0.92	0.92	0.92	**0.79**
50	0.90	0.90	0.89	0.88	0.86	0.89	0.90	0.90	0.90	**0.79**
100	0.89	0.90	0.87	0.89	0.88	0.89	0.90	0.89	0.89	**0.80**
150	0.90	0.89	0.88	0.90	0.87	0.90	0.89	0.89	0.89	**0.80**
200	0.89	0.90	0.89	0.90	0.86	0.90	0.90	0.90	0.90	**0.81**
500	0.87	0.87	0.89	0.88	0.90	0.89	0.88	0.90	0.87	**0.80**
**Insect stress in rice**										
10	0.88	0.90	0.88	0.93	0.81	0.64	0.82	0.82	0.82	**0.81**
20	0.89	0.89	0.89	0.88	0.84	0.80	0.85	0.88	0.88	**0.79**
50	0.90	0.87	0.90	0.88	0.84	0.86	0.86	0.89	0.89	**0.80**
100	0.90	0.88	0.90	0.88	0.87	0.86	0.84	0.90	0.90	**0.79**
150	0.90	0.88	0.90	0.88	0.86	0.86	0.85	0.89	0.89	**0.80**
200	0.90	0.89	0.90	0.88	0.85	0.86	0.85	0.89	0.89	**0.80**
500	0.90	0.89	0.88	0.87	0.87	0.84	0.87	0.89	0.89	**0.83**

Values marked as bolds represent dissimilarity scores obtained from proposed BSM approach

Document S8. Performance Evaluation of Gene Selection Methods Based on Runtime Criteria.

We used the runtime criteria to evaluate the performance of gene selection methods given in Table S9 of document S5. Here, the runtime refers to the amount of time required to get the informative gene sets of particular size through providing the gene expression data as input to the R functions of the respective methods. Here, we performed all analyses on a standard 8 GB RAM Dell PC with Intel(R) Core(TM) i3-6100U CPU @ 2.30GHz, 2301 Mhz, 2 Core(s) Processor(s) and recorded the computational time required to analyze Bacterial gene expression data of 8356 genes with 74 subjects (case: 37 and control: 37). Here, all the analyses are performed on R software with version 4.0.1. The result for bacterial datasets is shown in Table S20 and the results for the remaining datasets are shown in Table S21-23

Table S20. Runtime based analysis of gene selection methods in Bacterial stress dataset.

Sl. No.	Methods	Symbol	R package	Run time	Ranks	Rank score
01	BSM	BSM	BSM	25 Minutes	8	0.3
02	SVM (RFE)-MRMR	SVM-MRMR	BSM, e1071	1.30 hours	10	0.1
03	MRMR	MRMR	BSM	10 Minutes	7	0.4
04	SVM with Recursive Feature Elimination	SVM-RFE	e1071	1.20 Hours	9	0.2
05	t-score	t	stats	1.5 Minutes	1	1
06	F-score	F	stats	2 Minutes	2	0.9
07	Pearson's Linear Correlation	PCR	FSelector	4 Minutes	4	0.7
08	Information Gain	IG	FSelector	4.5 Minutes	5	0.6
09	Gain Ratio	GR	FSelector	5 Minutes	6	0.5
10	Wilcoxon Statistic	Wilcox	stats	2.5 Minutes	3	0.8

The BSM method required less computational time than SVM-RFE and SVM-MRMR with much better performance in terms of detecting biologically informative genes. Moreover, it can even be used on a PC or workstation computer for analyzing large gene expression datasets.

Table S21. Runtime based analysis of gene selection methods in Salinity stress dataset.

Sl. No.	Methods	Symbol	R package	Run time	Ranks	Rank score
01	BSM	BSM	BSM	15 Minutes	8	0.3
02	SVM (RFE)-MRMR	SVM-MRMR	BSM, e1071	75 Minutes	10	0.1
03	MRMR	MRMR	BSM	10 Minutes	7	0.4
04	SVM with Recursive Feature Elimination	SVM-RFE	e1071	70 Minutes	9	0.2
05	t-score	t	stats	1.5 Minutes	1	1
06	F-score	F	stats	2 Minutes	2	0.9
07	Pearson's Linear Correlation	PCR	FSelector	4 Minutes	4	0.7
08	Information Gain	IG	FSelector	4.5 Minutes	5	0.6
09	Gain Ratio	GR	FSelector	5 Minutes	6	0.5
10	Wilcoxon Statistic	Wilcox	stats	2.5 Minutes	3	0.8

Table S22. Runtime based analysis of gene selection methods in Drought stress dataset (9078 genes over 70 samples).

Sl. No.	Methods	Symbol	Run time	Ranks	Rank score
01	BSM	BSM	30 Minutes	8	0.3
02	SVM (RFE)-MRMR	SVM-MRMR	1.40 hours	10	0.1
03	MRMR	MRMR	15 Minutes	7	0.4
04	SVM with Recursive Feature Elimination	SVM-RFE	1.30 Hours	9	0.2
05	t-score	t	2.5 Minutes	1	1
06	F-score	F	3 Minutes	2	0.9
07	Pearson's Linear Correlation	PCR	5 Minutes	4	0.7
08	Information Gain	IG	5.5 Minutes	5	0.6
09	Gain Ratio	GR	6 Minutes	6	0.5
10	Wilcoxon Statistic	Wilcox	3.5 Minutes	3	0.8

Table S23. Runtime based analysis of gene selection methods in Fungal and insect stress datasets.

Sl. No.	Methods	Symbol	Run time	Ranks	Rank score
01	BSM	BSM	20 Min	8	0.3
02	SVM (RFE)-MRMR	SVM-MRMR	1hr 25 Min	10	0.1
03	MRMR	MRMR	10 Min	7	0.4
04	SVM with Recursive Feature Elimination	SVM-RFE	1hr 20 Min	9	0.2
05	t-score	t	1.5 Minutes	1	1
06	F-score	F	2.5 Minutes	2	0.9
07	Pearson's Linear Correlation	PCR	3 Minutes	4	0.7
08	Information Gain	IG	4 Minutes	5	0.6
09	Gain Ratio	GR	5 Minutes	6	0.5
10	Wilcoxon Statistic	Wilcox	3 Minutes	3	0.8

Document S9: Guidelines and Tutorials for BSM

R Package Inputs for BSM R Package

1. Gene Expression matrix $\boldsymbol{X}$ with N genes and M samples
2. Class information M-dimensional vector having elements +1 (case) and -1 (control)
3. Method for integrating SVM and MRMR weights. It can be either Linear or Quadratic.
   Linear method: Linear combination of weights computed through MRMR, and SVM methods and is given as:

$$SL_i = \beta w_i + (1-\beta)|k_i| \tag{7}$$

Quadratic method: The quadratic method for integration of weights can be expressed as:

$$SD_i = \frac{\beta \gamma_i^{MR} {w_i}^{norm} + (1-\beta)\gamma_i^{SV}|k_i|^{norm}}{\beta \gamma_i^{MR} + (1-\beta)\gamma_i^{SV}} \tag{8}$$

where, w_i and k_i are MRMR and SVM weights for i^{th} gene respectively; $\gamma_i^{MR}$and γ_i^{SV} are ranks MRMR and SVM weights for i^{th} gene respectively; ${w_i}^{norm} = \left(w_i - \min_i w_i\right)/(\max_i w_i - \min_i w_i\right)$, $|k_i|^{norm} = (|k_i| - \min_i |k_i|)/(\max_i |k_i| - \min_i |k_i|)$, and $\beta \in (0,1)$: integration constant.

4. A scalar representing trade-off between SVM and MRMR weights.
5. Number of bootsrap samples to be drawn from the data using simple random sampling with replacement (Bootstrap) procedure.
6. A method used for multiple hypothesis correction and computation of adjusted p-values. It can be any method out of "holm", "hochberg", "hommel", "bonferroni", "BH", "BY", "fdr".
7. Level of significance value (α) to decide the statistical significance of relevance of genes.

R package Installation from local directory:

Required e1071 R package
install.packages("…/…path location to the file…/BSM_0.1.0.tar.gz", repos = NULL, type = "source")
library(e1071)
library(BSM)

**** Data example
##Simulation of gene expression data
x <- as.data.frame(matrix(runif(1000), 50))
row.names(x) <- paste("Gene", 1:50)
colnames(x) <- paste("Samp", 1:20)
head(x) #######x is gene expression data matrix with 50 rows (genes) and 20 columns (samples)
y <- as.numeric(c(rep(1, 10), rep(-1, 10)))
y #####class labels for the samples (1: case and -1: control)

R package Installation steps from web:

#get the devtools
install.packages("devtools") ##install if not
iibrary(devtool)
#install BSM R package from web
install_github("sam-uofl/BSM")
library(BSM)

Computation weights for gene selection through BSM method.

> bootsvmmrmrwt(x, y, method = "Linear", beta = 0.5, nboot = 50)

Computation statistical significance values for gene selection through Bootstrap-Support Vector Machine (SVM)-Maximum Relevance and Minimum Redundancy (MRMR) methods.

> pvalsvmmrmr(x, y, method, beta, nboot, p.adjust.method, plot)

Selects the top ranked (differentially expressed) genes through Bootstrap-SVM-MRMR method.

> TopGenesBootSVMRMR(x, y, method = "Linear", beta = 0.5, nboot = 50, n = 20)

Selects the top ranked (differentially expressed) genes through MRMR method.

> TopGenesMRMR(x, y, n = 20)

Selection of genes based on statistical significance values computed through BSM method.

> TopGenesPvalSVMRMR(x, y, method = "Linear", beta = 0.5, nboot = 50, p.adjust.method = "BH", n = 20)

Selection of top ranked (differentially expressed) genes through SVM method

> TopGenesSVM(x, y, n = 20)

Selects the top ranked (differentially expressed) genes through SVM-MRMR method.

> TopGenesSVMRMR(x, y, method = "Linear", beta = 0.5, n =20)

#**Computation of gene ranking weights through MRMR method.**

> weightmr(x, y)

#**Computation of weights for gene selection through SVM algorithm**

> weightsvm(x, y)

#**Computation weights for gene selection through SVM-MRMR method.**

> weightsvmmrmr(x, y, method, beta)

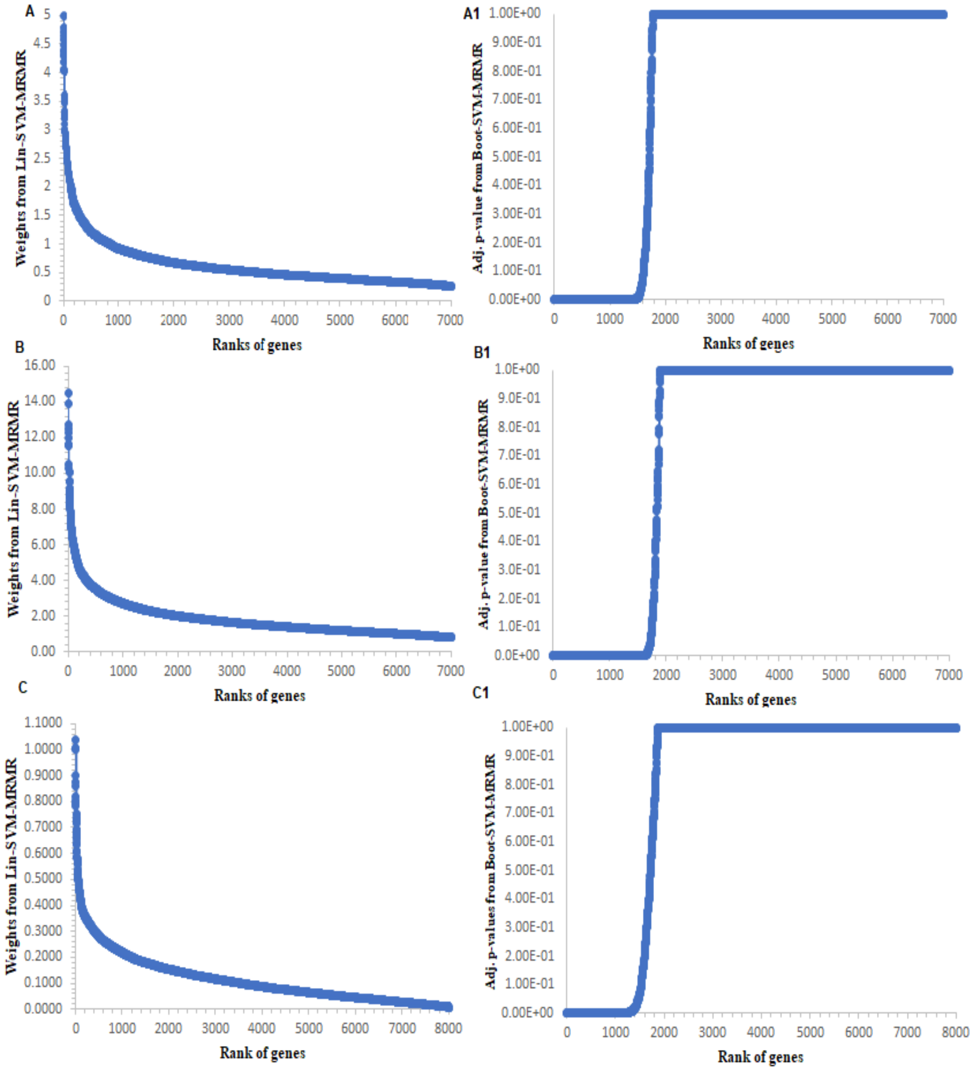

Figure

Figure S3. Graphical analysis of the proposed Boot-SVM-MRMR approach with Lin-SVM-MRMR approach for biotic stress datasets. Distribution of gene weights computed from SVM-MRMR approach for the biotic stresses. The distributions of weights from the SVM-MRMR are shown for (A): Bacterial; (B): Fungal; and (C): Insect stress datasets in rice. Distribution of adjusted gene *p-values* computed from for (A1): Bacterial; (B1): Fungal; and (C1): Insect stress datasets in rice BSM approach

for the biotic. stresses. The distributions of adjusted *p-values* are shown

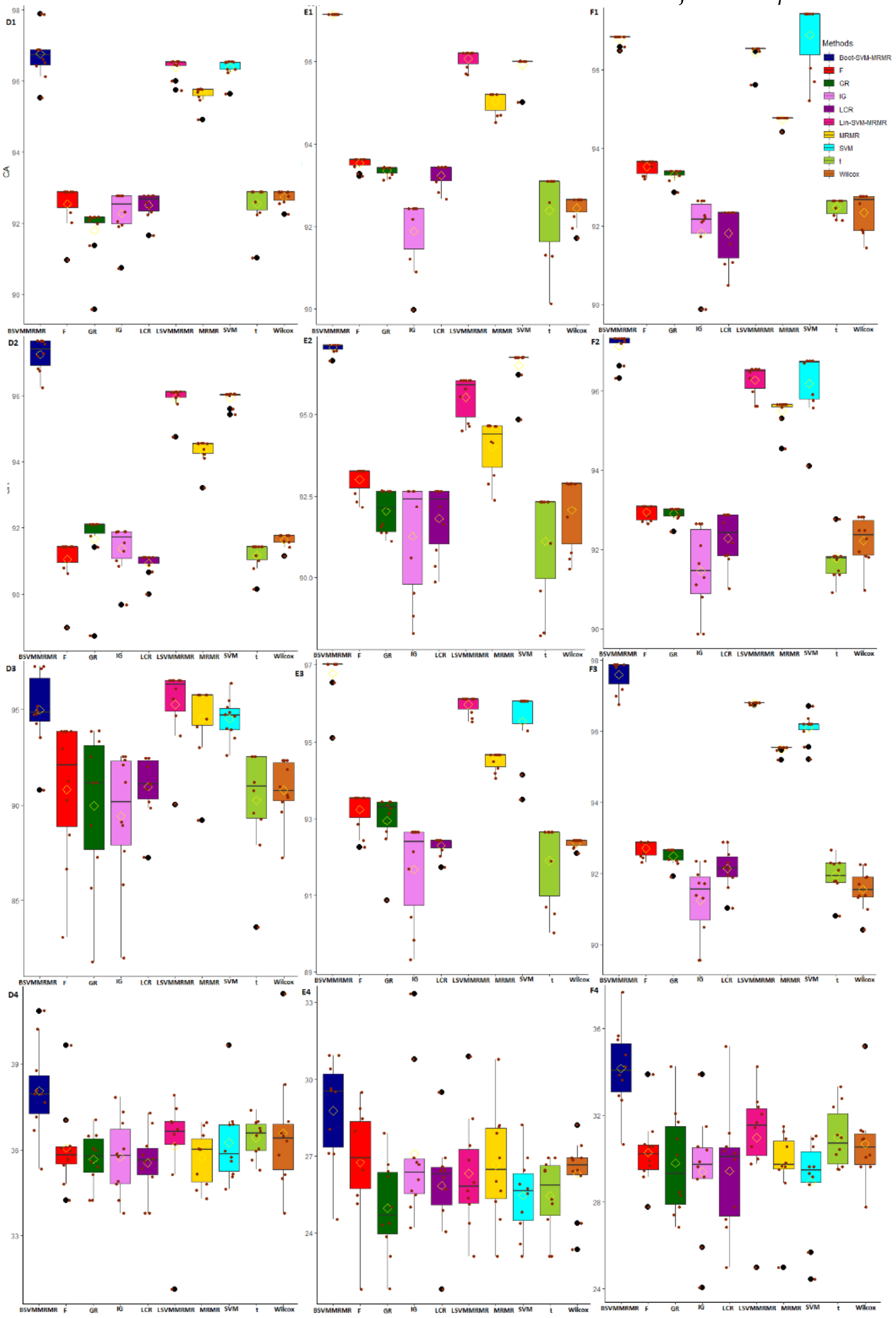

Figure S4. Classification based comparative performance analysis of gene selection methods in biotic stress datasets. The horizontal axis represents the gene selection methods. The vertical axis represents post selection classification accuracy obtained by using varying sliding window size technique. The classification accuracies over the window sizes are presented as boxes. The bars on the boxes represents the standard errors. The distributions of classification accuracies are shown for rice Bacterial stress dataset with SVM-LBF classifier (D1), SVM-PBF classifier (D2) SVM-RBF classifier (D3), and SVM-SBF classifier (D4); The distributions of classification accuracies are shown for rice Fungal stress dataset with SVM-LBF classifier (E1), SVM-PBF classifier (E2) SVM-RBF classifier (E3), and SVM-SBF classifier (E4) classifiers; The distributions of classification accuracies are shown for rice Insect stress dataset with SVM-LBF (F1), SVM-PBF (F2), SVM-RBF (F3), and SVM-SBF (F4) classifiers.
